# Network pharmacology of olive stem extract, UPLC-HR-QTOF-MS profiling and antiviral activities aligned with UN sustainable development goals

**DOI:** 10.1038/s41598-025-07452-1

**Published:** 2025-07-02

**Authors:** Yasmin Mounir Mohamaden, Seham S. El-Hawary, Esmail M. El‑Fakharany, Yousra A. El‑Maradny, Mohamed El Raey, Amira Safwat El Senousy, Samar M. Bassam

**Affiliations:** 1 Department of Pharmacognosy, Faculty of Pharmacy, Cairo University, Kasr El Aini 11562, Cairo, Egypt; 2 Protein Research Department, Genetic Engineering and Biotechnology Research Institute (GEBRI), City of Scientific Research and Technological Applications (SRTA-City), New Borg El-Arab City 21934, Alexandria, Egypt; 3 Pharmaceutical and Fermentation Industries Development Centre (PFIDC, City of Scientific Research and Technological Applications (SRTA-City), New Borg Al-Arab, Alexandria, Egypt; 4 Microbiology and Immunology, Faculty of Pharmacy, Arab Academy for Science, Technology and Maritime Transport (AASTMT), Alamein, 51718 Egypt; 5 Department of Phytochemistry and Plant Systematics, Pharmaceutical Division, National Research Centre, Dokki, Cairo, Egypt; 6 Department of Pharmacognosy and Natural Products, Faculty of Pharmacy, Pharos University in Alexandria, Beside Green Plaza Complex, Canal El Mahmoudia street, 21648 Alexandria, Egypt

**Keywords:** Olive stem, Phenolics, Iridoids, LC-MS/MS, Antiviral activity, Phytomedicine, Computational biology and bioinformatics, Drug discovery, Plant sciences

## Abstract

*Olea europaea* tree has long been a cornerstone of traditional medicine. Its different parts have been used as a remedy for viral accompanied diseases as asthma, sore throat, fever, and others. Despite its historical significance, olive stem’s chemical diversity and pharmacological potential remain underexplored. This study aims to bridge that gap by identifying the phytochemical compounds in stem ethanolic extract using ultra-performance liquid chromatography coupled to high-resolution quadrupole time of flight mass spectrometer (UPLC-HR-QTOF-MS). Additionally, it assesses the extract’s cytotoxic effects using MTT assay and antiviral activities against HSV-1, coxsackievirus-B4, and adenovirus-7. Network pharmacology was employed to understand the potential compound-target pathways. The analysis revealed 119 compounds across 11 classes, including 9 new, such as ligstrosidic acid, nuzhenal A, and syringaresinol derivatives. Significant antiviral activity was demonstrated, with selectivity indices (SI) of 30.78 and 27 for HSV-1 and adenovirus, respectively. Eight anti-HSV-1 targets and 18 pathways were predicted, including NFkB, CHUK, and PIK3R1. The study highlights the value of olive stem waste products as antiviral agents, particularly against HSV-1, supporting their traditional use. It provides an approach to developing low-cost pharmaceutical products and animal feed for combating zoonotic agents, aligning with the 2030 UN Sustainable Development Goals (SDG-3 and SDG-12) for promoting health and sustainable resource use.

**Supplementary Information:**

The online version contains supplementary material available at 10.1038/s41598-025-07452-1.

## Introduction

Infectious diseases remain a major health issue, making up 41% of the global disease burden measured in Disability-Adjusted Life Years (DALYs)^1^. As recently reported, viruses cause significant health issues in humans and animals. These include herpes simplex virus type 1 (HSV-1), known as cold sores, with potential complications in the eye, skin, visceral organs, and central nervous system encephalitis in adults^2,3^. Additionally, coxsackievirus, can cause fatal complications like encephalitis, sepsis, myocarditis, heart and breathing problems like apnea and arrhythmias^4^. Adenoviruses (Adeno-7) primarily affect the respiratory, conjunctiva, and gastrointestinal systems, with complications including acute respiratory distress syndrome and viral meningitis^5^. These viruses can infect animals and humans. HSV-1 has been studied on animal models, including mice as described in^6^, while coxsackievirus affects non-human primates and livestock^7^. Also, adenoviruses infect whole categories of vertebrates, including birds, mammals, fish, and reptiles (Adenovirus | Cornell Wildlife Health Lab); depending on the type/species of virus, it can infect various hosts.

Olive trees, *Olea europaea*, belong to the Oleaceae family and symbolize peace, wisdom, joy, and longevity. Historically, their leafy branches were used to crown victors^8^. This family comprises 24–28 genera of 700–750 species of evergreen trees, flowering shrubs, and lianas^9^. Cultivated for around 6000 years, olives are primarily grown in the Mediterranean Basin and North Africa^10^.

The olive tree has been a vital part of traditional phytomedicine, with its leaves, root, bark, stem, seeds, fruit, and oil used for a variety of ailments. Leaf-based products are used in different forms and routes for a range of treatments. In Arabic countries, dried plant fumigation is used nasally for abortifacient purposes and for cystitis and sore throat. In Brazil, fresh leaves herbal tea induces diuresis and treats hypertension. Canary Islands use leaf infusions orally or rectally, to manage diabetes, hypertension, and hemorrhoids. In France, oral capsule form of leaves promote urinary and digestive emptying. Germany uses ethanol extracts to treat atherosclerosis and hypertension. Italians employ for hypertension, inflammation and wound healing. In Morocco, leaves and oil are used both orally and topically to treat stomach and intestinal diseases, and as a hair tonic. Ukraine uses hot water extracts to treat bronchial asthma^11^. Other traditional uses include treatment and prevention of atherosclerosis, anaplasmosis, helminthiasis, rheumatism, and lumbago, ophthalmic remedies, and antiperspirant^11–14^. Olive oil is exploited to promote blood circulation and treat rheumatism in Italy and as a laxative in Oman^15^. Evidence-based medicine has sufficiently reported these uses in literature^16^.

These activities are the merit of olive powerful secondary metabolites, mainly phenolic derivatives such as hydroxytyrosol, elenolic acid, oleuropein, and ligstroside. Additionally, cinnamic acid derivatives with the predominantly verbascoside, and flavonoids^17,18^. The most abundant biophenol in olive leaves is oleuropein, claimed by a U.S. patent to have potent antiviral activities against herpes mononucleosis, rotavirus, hepatitis virus, canine parvovirus, bovine rhinovirus, and feline leukemia virus^19^.

Research has focused on olive leaves, oil, and byproducts; olive mill wastewaters and olive pomace and their medicinal value. However, few have dealt with olive stems, considered agro-industrial residues, even though present in large quantities due to annual pruning^20^, , and represent an environmental problem if not processed correctly^21^. These by-products (stems) are rich in phenolic compounds that are generally one of the most important groups of natural antioxidants^22^.

For a deeper insight into the chemical composition of olive stem extracts, liquid chromatography coupled to a high-resolution QTOF mass detector was chosen for analysis. Kabbash et al. have reported that this technique is the most used for metabolomics analysis of olive leaves^23^. LC-MS was also employed for phytochemical investigation of olive roots^24^, seeds and stones^25^, and others. GC-MS is usually a technique for the investigation of volatiles, which narrows its application. Despite this, terpenes and flavor components were investigated in olive oils using GC-MS by^26^. Comparing LC-MS with GC-MS, sample preparation and analysis sensitivity are better in LC-MS, however, GC-MS sometimes requires derivatization. LC-MS can detect almost all types of compounds, though repeatability was a concern as reported by^27^. For metabolomics analysis, LC-NMR is usually used as a complement to other techniques, as it is less sensitive, requires a larger sample size, in addition special solvents. Its advantage is the specific characterization of the compounds^28^. The major challenge in LC-MS is the matrix effect, which threatens its sensitivity and selectivity by affecting the efficiency of ionization because of either endogenous or exogenous suppressors^29^.

Network pharmacology uses biological networks to illustrate how various bioactive compounds affect multiple targets. This approach, known as a compound-target-pathway network, combines system biology, bioinformatics, and network science to unravel the complex interactions between drugs and their targets. It helps guide drug discovery and development by analyzing how these compounds interact with related diseases through multiple pathways. This analysis accurately predicts targets and establishes a model for “drug bioactive disease”^30,31^.

The objective of the current study is to identify the phytochemical composition of the olive stem extract (OSE) using UPLC-HR-QTOF-MS in negative ionization mode. Aligned with the traditional anti-viral use of olive, this study investigated the in vitro antiviral activities against herpes simplex virus type-1 (HSV-1), coxsackievirus type-B4 (CB-4), and adenovirus type-7 (Adeno-7), both anti-replicative and direct virucidal effects. Subsequently, the most intense ions from LC-MS/MS were selected based on peak area for exploring the pharmacological effects using network pharmacology on the most susceptible virus, providing a basis for additional experimental investigation. This article provides an insight to implementation of the 2030 UN Sustainable Development Goals.

## Materials and methods

### Chemicals and standard

Ethanol and ultrapure water for extraction (Sigma-Aldrich, Germany). Formic acid 98%, methanol, sodium hydroxide, for pH adjustment (Fisher Scientific, UK), ammonium formate, acetonitrile (Sigma-Aldrich), and water (Milli-Q) (Millipore, USA) were of LC grade.

### Plant material and extraction

#### Plant material

Olive stems (*Olea europaea* L. *cv* Soury), separated from the leaves, were collected from the Agriculture Research Station, Faculty of Agriculture, Cairo University, Egypt, in October 2022 during harvest. The plant was authenticated by Dr. Abdou Mohamed Abdallatif, a botanist at the Department of Pomology, Faculty of Agriculture, Cairo University. Olive stems were washed thrice with tap water, dried in the shade, finely ground, and stored in tightly sealed dark glass containers.

#### Extraction protocol

Dried powdered stems (450 g) were extracted with ethanol 70% three times at room temperature. The extract was filtered through filter paper (Whatman no.1), then concentrated under reduced pressure using a rotary evaporator at 40^°^C to yield a dark brown residue (∼32.70 g).

### Analysis by UPLC-HR-QTOF-MS

#### Sample Preparation

OSE was analyzed by ultra-performance liquid chromatography coupled to a high-resolution quadrupole time of flight mass spectrometer (UPLC-HR-QTOF-MS) operated in negative ionization mode as described in^32^. A stock solution was prepared by dissolving 50 mg of lyophilized extract in 1 mL of a solvent mix (methanol, acetonitrile, and water in a 25:25:50 ratio) (v/v), vortexing **for 2 min** and ultrasonication **at 30 kHz for 10 min**. Subsequently, 50 µL of stock solution was diluted with 1000 µL of another solvent mix (H_2_O: MeOH: ACN in a 50:25:25 ratio), centrifuged **at 10**,**000 rpm for 10 min**, and 10 µL (2.5 µg/µL) were used for injection.

#### Instruments and acquisition method

Separation utilized an ExionLC system with an autosampler, pre-column filter (0.5 μm ×3.0 mm), and an X select HSS T3 (2.5 μm, 2.1 × 50 mm) column at 40 °C and 0.3 mL/min flow rate. Solution A was 5 mM ammonium formate in 1% methanol (pH 8), and solution B was 100% acetonitrile. The gradient elution program was: 0–20 min, 10% B; 21–25 min, 90% B; 25.01–28 min, 10% B; followed by column equilibration at 90% B.

A Duo-Spray source in ESI mode was used. The negative mode settings included − 4500 V for the sprayer capillary and − 80 V for de-clustering potential. The operating conditions were 500 °C source temperature, 25 psi curtain gas, and 45 psi for gases 1 and 2. The collision energy was − 35 V with a CE spread of 15 V and ion tolerance of 10,000 ppm. The TripleTOF5600 + used a data-independent acquisition (DIA) protocol for data collection. High-resolution survey spectra ranged from 50 to 1000 *m/z*, with a 50-ms survey scan determined^32,33^.

#### LC-MS/MS data processing

The LC-MS/MS run file underwent conversion into .mzML via msConvertGUI software. The converted file was submitted to MZmine 2.53 software and PeakView 1.2.0.3 for peak detection, filtering and deconvolution. Metabolites were identified through their mass spectra, which were compared with databases and pertinent reference literature^34^.

#### Databases

For the retrieval of chemical structure information, the following databases were consulted available by PubChem (PubChem (nih.gov)), FooDB (FooDB, accessed between June 2023 and December 2024), PlantaeDB (https://plantaedb.com, accessed on February 15, 2024), Phenol-Explorer version 3.6 (Database on Polyphenol Content in Foods - Phenol-Explorer), ChemSpider (ChemSpider | Search and share chemistry), J-GLOBAL (https://jglobal.jst.go.jp/en), and using ChemCalc (https://chemcalc.org/mf-finder) followed by Dictionary of Natural Products (DNP) (https://dnp.chemnetbase.com, accessed between January 2024 and May 2024) to find the mass formula and predict the structure of certain unidentified compounds, respectively. Moreover, the exact masses were calculated using the Exact Mass Calculator (https://www.sisweb.com/referenc/tools/exactmass.htm). Structures drawing was accomplished using ChemDraw Professional version 15.0.0.106.

### Antiviral assay

#### Cell line and virus

African Green Monkey kidney epithelial cell lines (*Vero* cells) were obtained from the American Type Culture Collection (ATCC) *via* VACSERA (Cairo, Egypt). Herpes simplex virus type 1 (HSV-1), coxsackievirus type B4 (CB-4), and adenovirus type 7 (Adeno-7) were kindly provided from the antimicrobial activity unit in The Regional Center for Mycology and Biotechnology, Al Azhar University.

#### Cell viability and cytotoxicity

The cytotoxic effects of the OSE were tested on *Vero* cells using the MTT assay. Acyclovir was used as the reference standard antiviral compound for comparison. Cells were incubated with various extract concentrations, and cell viability was measured by optical density after 72 h which were thoroughly detailed in^35^. The percentage of cell viability in relation to untreated cells was computed utilizing the subsequent formula:

$${\text{Cell}}\;{\text{viability}}\;\left( \% \right)=\frac{{\left( {{\text{Mean}}\;{\text{OD}}\;{\text{of}}\;{\text{treated}}\;{\text{cells}}\;--\;{\text{blank}}\;{\text{Control}}} \right)}}{{{\text{Control}}\;\left( {{\text{untreated}}\;{\text{cells}}} \right)\;{\text{OD}}--{\text{blank}}}}$$

#### Virus stock solution Preparation

Stock solutions were formulated for three cytopathic viruses: HSV-1, coxsackievirus type B4 (CB-4), and Adeno-7. These preparations were utilized to quantify the 50% tissue culture infectious dose (TCID50) and stored at -80 °C until needed^35^.

#### In vitro antiviral activity

The antiviral activity of the compound was tested for both treatment (anti-replicative) and neutralization (direct virucidal) effects on virus stocks using *Vero* cells. Various concentrations of the OSE were applied, and the 50% inhibitory concentration (IC_50_) was calculated using GraphPad Prism 8. The experimental methods were comprehensively explained according to^35^.

#### Antiviral activity against viral antigens

The levels of HSV-1 and human adenovirus antigen (HAdV-Ag) were assessed in the supernatant and cell lines and measured using ELISA kits after treating infected *Vero* cells with the extract. The processes and data are elaborately explained by^35^. The percentage of viral inhibition was calculated based on antigen reduction.

#### Statistics

Data were analyzed using GraphPad Prism 8 for Windows version 10 to determine the effective concentration for 100% viability (EC_100_) and the half-maximal cytotoxic concentration (CC_50_)^36^.

### Network Pharmacology

#### Collection of active compounds

The bioactive compounds of olive OSE were collected by selecting the most intense ions, prioritizing those with the highest peak area obtained from LC-MS/MS, and identified using different databases, as described previously.

#### Predicting the targets of the compounds and screening for disease-related genes

To identify potential targets of the most abundant compounds, the SuperPred online platform (prediction.charite.de/subpages/target_prediction.php, accessed on June 22, 2024) was utilized. Target predictions were obtained by inputting the canonical SMILES of the compounds. Strong binding genes and predicted targets with a probability of 75% were selected. The corresponding gene names for the proteins were retrieved from STRING by entering the protein names and extracting the UniProt ID or official gene symbol^37,38^.

For the screening of genes-related disease, genes were obtained from (GeneCards - Human Genes | Gene Database | Gene Search, accessed on June 22, 2024), using “HSV-1” as the term in the search query, choosing > 55 GeneCards Inferred Functionality Scores (GIFs).

The online tool Venny (Venny 2.1.0 (csic.es), accessed on June 22, 2024) was used to generate a Venn diagram comparing the targets of OSE and HSV-1. The overlapping section of the diagram highlights the anti-HSV-1 targets associated with olive stem^30^.

#### Network creation of protein-protein interactions^39^

The protein-protein interactions network for anti-HSV-1 targets of the olive stem was constructed utilizing the STRING database (STRING: functional protein association networks (string-db.org), accessed on June 23, 2024), selecting ‘Homo sapiens’ as the organism. A confidence score of 0.150 was applied to ensure a broader inclusion of potential interactions, allowing the identification of weaker or less-studied connections that may contribute to understanding anti-HSV-1 targets. Disconnected nodes were excluded from the network through advanced settings. Additionally, KEGG pathway analysis was conducted to hypothesize biological roles associated with these targets. Cytoscape software version 3.7.2 has been used to create and visualize the network between three components: active compounds, matching target genes (anti-HSV-1 targets), and pathways related to the disease (HSV-1). This visualization is shown as a node (compounds, targets, and pathways) and an edge (interaction between different nodes)^40^.

## Results and discussions

### LC-MS/MS analysis and identification of Olive stem extract components

**Fig. 1:**
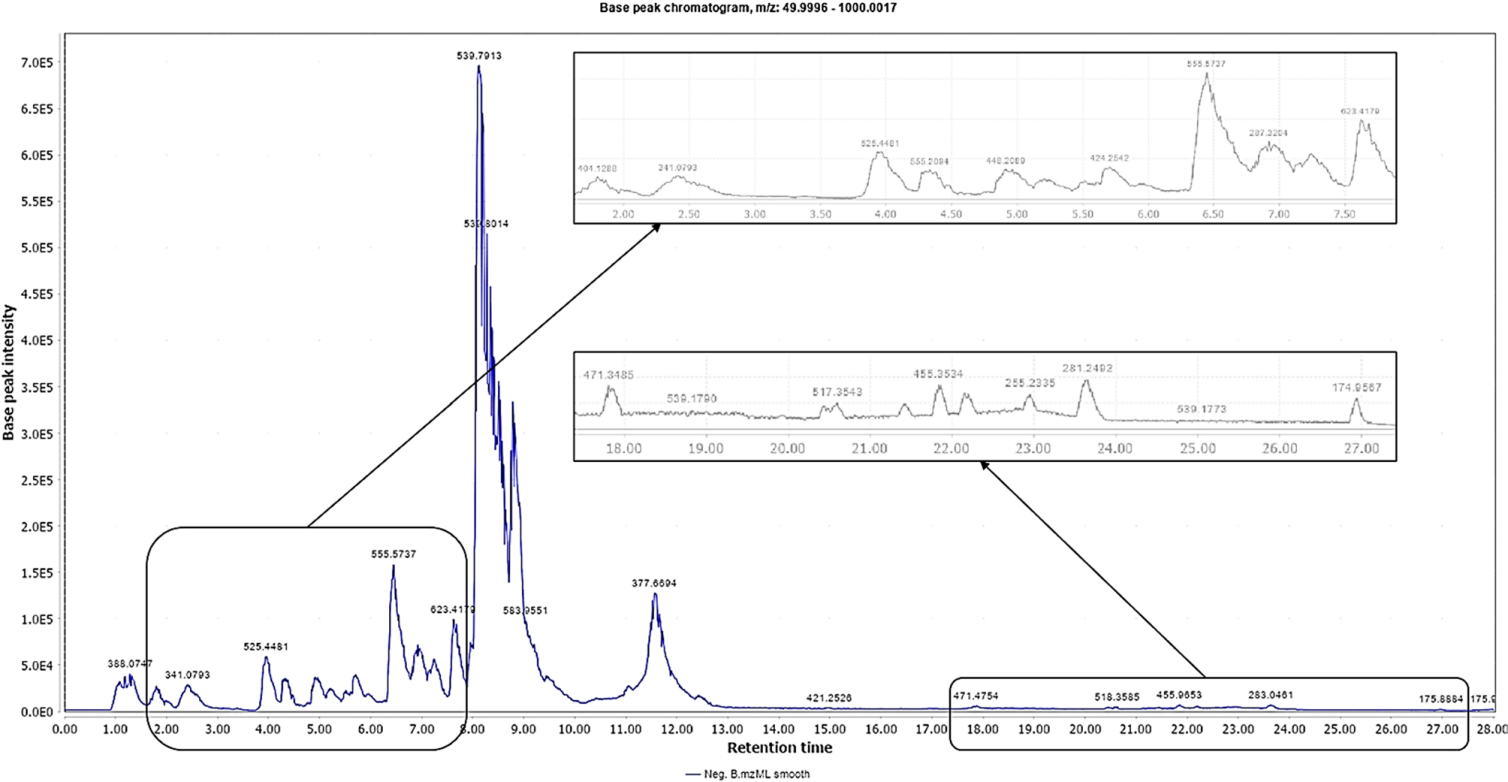
Base Peak Chromatogram (BPC) of ethanolic extract of olive stems using UPLC-HR-QTOF-MS detection in the negative ionization mode.

Metabolite profiling of OSE using UPLC-HR-QTOF-MS in negative ionization mode is demonstrated in. Figure 1. A total of (119) compounds are listed in Table S1 belonging to various phytochemical classes, Table S1 sums up compound name, molecular formula, molecular weight/ exact mass, the retention time (Rt, min), *m/z* of theoretical, experimental, and adduct ions, error (ppm), MS/MS fragment ions (*m/z*) along with the relevant references.

The identified compounds and their isomers represented (11) structurally distinct classes: (I) sugar derivatives (5), phenolic acids hydroxybenzoic acid derivatives (6), (III) phenylethanoids derivatives (9), (IV) iridoid glycosides (3), (V) secoiridoids (39) that were divided into different subclasses according to the basic structure skeleton; oleoside and secologanoside derivatives (5), oleuropein-type secoiridoids (23), ligstroside-type secoiridoids (6), nuzhenide-type secoiridoids (2 compounds), and other secoiridoids (3), (VI) lignans and derivatives (16), (VII) hydroxycoumarins and derivatives (4), (VIII) flavonoids (16 compounds) including flavanones, flavanonols, flavones, and flavonols, (IX) terpenes (8), (X) fatty acids (6) involving saturated and unsaturated fatty acids (mono- and polyunsaturated fatty acids), and (XI) other compounds (7). All tentatively identified through their exact masses and MS^2^ fragmentation, and by comparison with the available literature. Furthermore, adduct ions as [M-H + HCOOH]^−^, [M-H + FA]^−,^ and [M + Cl]^−^ were observed and supported the confirmation of EIC for the [M-H]^−^ ion.

Based on peak area semiquantitative calculations, oleuropein was the most abundant metabolite, representing 11.003%, followed by lucidumoside C (2.779%), oleuropein aglycone (2.472%), acetoxypinoresinol hexoside (1.234%), and hydroxyoleuropein (1.209%). This value is significantly higher than those reported by^41^ for *Olea europaea* stems, where oleuropein content ranged between 1.097% and 1.462%. Ortega-García and Peragón reported the increasing stem concentration of oleuropein during ripening, ranging from 2.23% in August to 5.8% in November^42^.

#### Sugars

Five monosaccharide compounds were detected (Fig. S1A). Gluconic/galactonic acid and threonic acid were detected at *m/z* 195.0511 and 135.03 [M-H]^−^, respectively, exhibiting the major peak *m/z* 75.01 for gluconic acid. Water loss led to fragments ions at 177.04 [M-H-H_2_O]^−^ followed by double loss of H_2_O at *m/z* 159.03, *m/z* 129.02 [M-H-2H_2_O-CH_2_O]^−^ subsequently the loss of CO_2_ at *m/z* 101.02, 111.01 [M-H-3H_2_O-CH_2_O]^−^, and base peak at *m/z* at 75.01^11,43–45^. Sucrose adduct [M-H + FA]^−^ was detected at *m/z* 387.16, with a base peak at *m/z* 341.11 [M-H]^−^ (Table S1**)**. Mannitol/sorbitol appeared at *m/z* 181.07 [M-H]^−^.

**Fig. 2:**
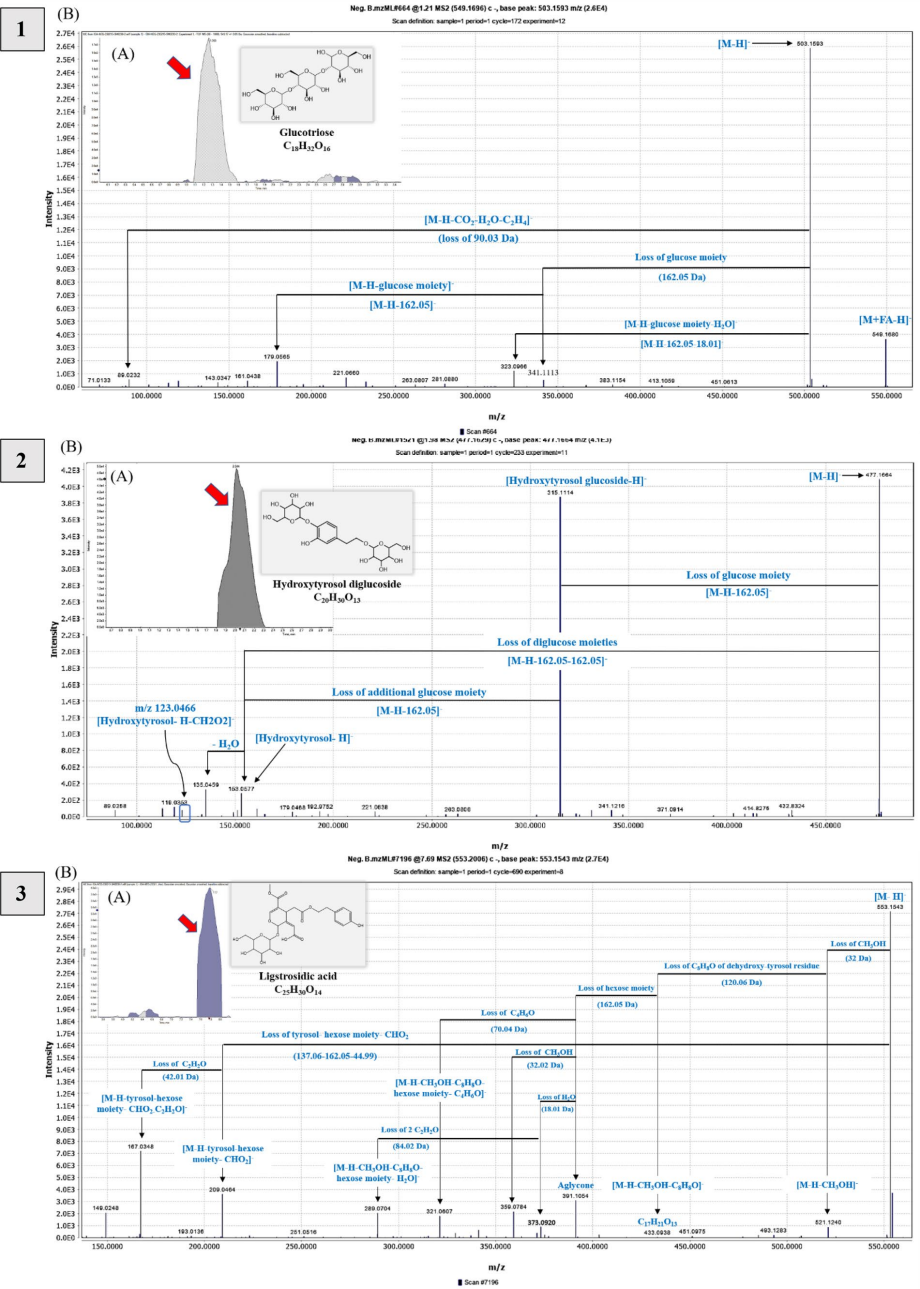

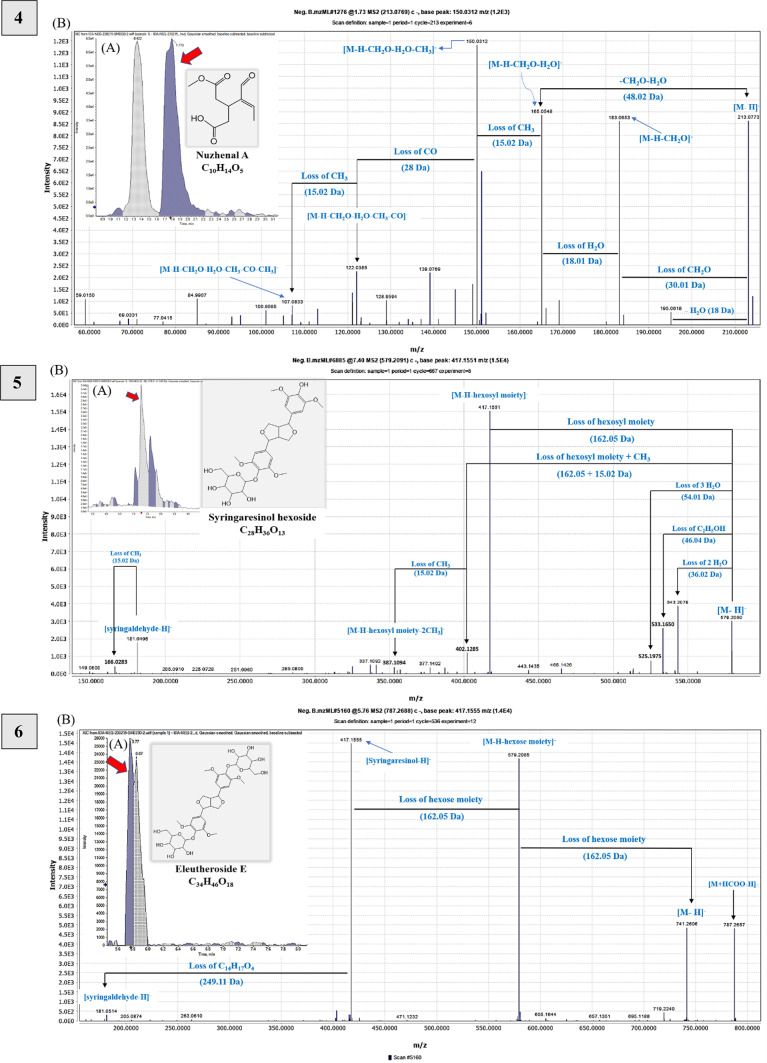

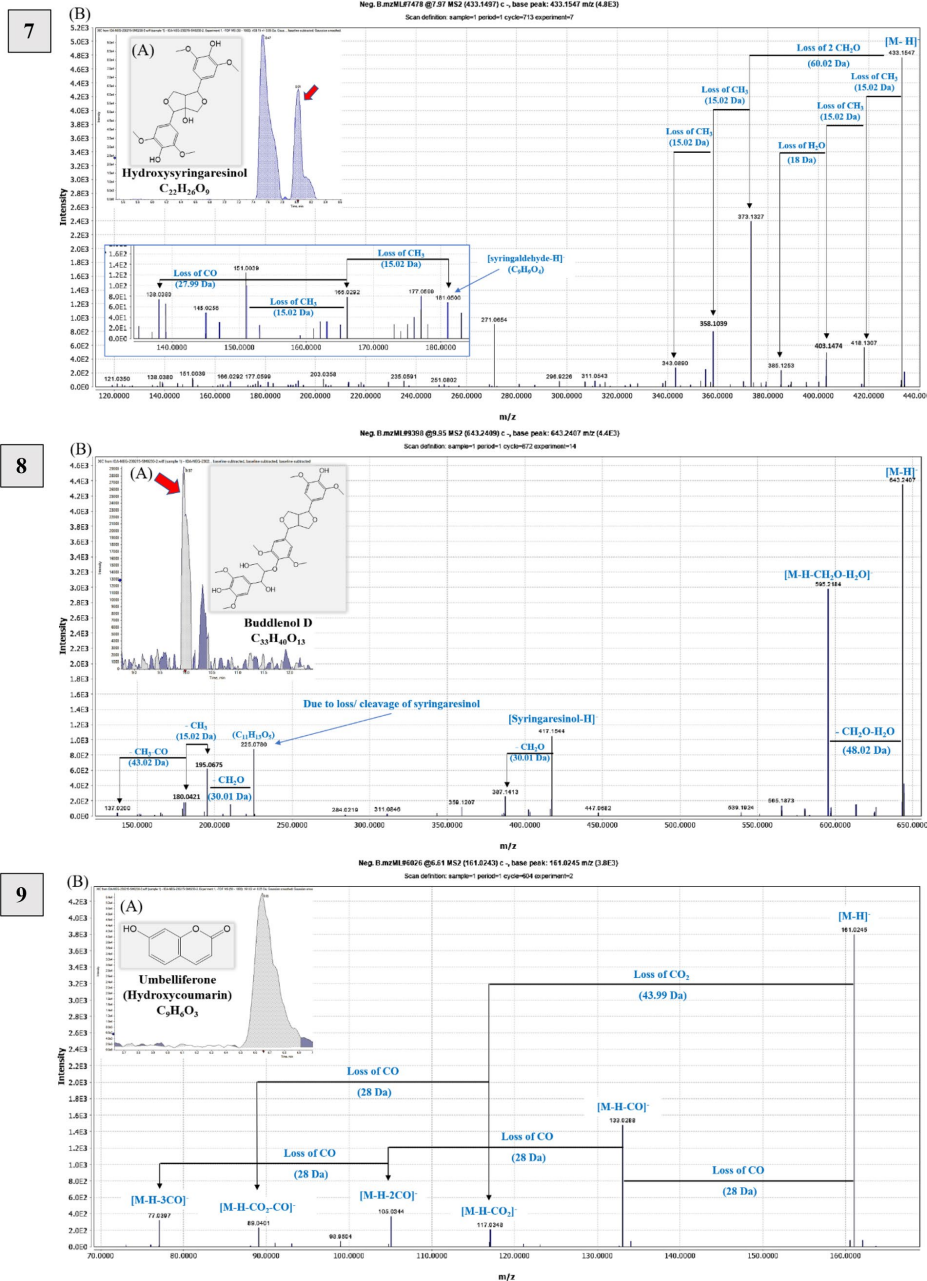
Novel compounds tentatively identified in the olive (*Olea europaea* L. *cv*. Soury) stem ethanol extracts detected by UPLC-HR-QTOF-MS in negative ionization mode showing (A) extracted ion chromatograms (EICs), representative chemical structures, and molecular formula; (B) MS/MS spectra and fragmentation pathway of compounds: Glucotriose—1) at *m/z* 549.17 as [M + FA-H]^-^; Hydroxytyrosol dihexoside—2) at *m/z* 477.16 as [M-H]^-^; Ligstrosidic acid—3) at *m/z* 553.15 as [M-H]^-^; Nuzhenal A—4) at *m/z* 213.08 as [M-H]^-^; Syringaresinol hexoside—5) at *m/z* 579.20 as [M-H]^-^; Eleutheroside E—6) at *m/z* 787.27 as [M-H + FA]^-^; Hydroxysyringaresinol—7) at *m/z* 433.15 as [M-H]^-^; Buddlenol D—8) at *m/z* 643.24 as [M-H]^-^; Umbelliferone—9) at *m/z* 161.02 as [M-H]^-^.

Glucotriose was identified for the first time as [M + FA-H]^−^ at *m/z* 549.17. The parent ion represents the base peak [M-H]^−^ at *m/z* 503.16 of C_18_H_31_O_16_, confirmed by fragment ions due to hexose, then water loss at *m/z* 341.11 323.09 and 179.05, 89.02 [M-H-CO_2_-H_2_O-C_2_H_4_]^−^, as described in Table S1 and Fig. 2.^46,47^.

#### Phenolic acids and their derivatives

Six phenolic acids were identified (Fig. S1**B**); hydroxybenzoic acid compounds, such as vanillic acid (*m/z* 167.03) and (*m/z* 329.08) its glucoside (Table S1). Protocatechuic acid and its glucoside were detected at *m/z* 153.02 and 315.07, respectively. Besides, dihydroxybenzoic acid hexoside pentoside (*m/z* 447.11) and *ρ*-hydroxybenzoic acid (*m/z* 137.02) were also detected. The explanation of the MS2 spectrum is found in Table S1.

#### Phenylethanoid derivatives

Phenylethanoid compounds like tyrosol (Tyr/3-HPEA) and hydroxytyrosol (HTyr/3,4-DHPEA) (Fig. S2 A) are found in *Olea europaea* and are precursors to secoiridoids such as oleuropein and ligstroside^48^. The fragmentation patterns of the following identified compounds were described in detail in Table S1. 3,4-Dihydroxyphenylglycol (DHPG) was detected at m/z 169.05, with base peak at m/z 151.04. HTyr revealed a precursor ion at m/z 153.06, showing a major peak at *m/z* 123.04. Hydroxytyrosol hexoside was found at *m/z* 315.11[M-H]^-^ and *m/z* 361.11 [M + FA-H]^-^ adduct, showing a fragment at *m/z* 153.05 (hexosyl loss), while tyrosol hexoside was detected at *m/z* 299.11. A peak at *m/z* 481.20 [M-H]^-^ had the same fragmentation pattern so it was deduced to be Hydroxytyrosol hexoside derivative^49^.

Additionally, hydroxytyrosol dihexoside was observed at *m/z* 477.16 [M-H]^-^, losing hexose to give *m/z* 315.11 and 153.05 [HTyr-H]^-^, as mentioned in Table S1 and Fig. 2. Previous research has identified this compound in olive oil byproducts^11^.

Compounds derived from both HTyr and hydroxycinnamic acid (also known as caffeoyl phenylethanoid) are linked by a pyranose unit via a glycosidic bond to which a rhamnosyl moiety is attached (Fig. S2 A)^50^. Hydroxyacteoside/hydroxyverbascoside was observed at *m/z* 639.20 [M-H]^-^. Likewise, verbascoside/acteoside was detected at *m/z* 659.17 [M + Cl]^-^ adduct and isoverbascoside/isoacteoside, eluted after verbascoside (Table S1) as mentioned by^46^.

#### Iridoid glycosides

Iridoids in *Olea europaea* are monoterpenes with a cyclopentapyran structure (Fig. S3 A)^51^. Loganic acid was identified at Rt of 1.12 min with a molecular ion [M-H]^−^ at *m/z* 375.13. Loganin (Fig. S2 B) was identified in two isomer from: isomer 1 with [M-H]^−^ at *m/z* 389.14 and isomer 2 with [M + FA-H]^−^ at *m/z* 435.15, showing common MS/MS peak ions (Table S1).

#### Secoiridoids, glycosides, and derivatives

Secoiridoids, a class of monoterpenes derived from iridoids by breaking the C7-C8 bond, are characteristic to the olive tree, as revealed in the general structure in Fig. S3 B^52^. These compounds can be classified into different subgroups based on the nucleus of the parent skeleton and showed fragments in detail in Table S1, which include:

##### Oleoside and secologanoside derivatives

Elenolic acid, characterized by dicarboxylic acids at positions C-7 and C-11, can be found in both free (Fig. S3 C) and ester forms^47^. It serves as a precursor for health-beneficial secoiridoid compounds^53^. Elenolic acid glucoside/oleoside methyl ester is a degradation product of ligstroside and oleuropein. Various oleoside and secologanoside derivatives were identified in OSE as shown in Fig. S4 A.

Elenolic acid dihexoside exhibited [M + FA-H]^−^ adduct at *m/z* 611.18, showing [M-H]^−^ *m/z* 565.18, and hexopyranosyl dimethyloleoside [M-H]^−^ at *m/z* 951.29. Both compounds have the same major peak *m/z* 403.12, corresponding to the deprotonated ion of elenolic acid hexoside. Additionally, the isomers oleoside and secologanoside were observed at *m/z* 389.11 [M-H]^−^ and *m/z* 425.08 [M + Cl]^−^ adduct, respectively, differing in the double bond positions (olefin/exocyclic double bond): oleoside between C-9 and C-8, and secologanoside between C-8 and C-10 (Fig. S4 A). Secologanoside eluted after oleoside according to previous reports^49,54,55^. Meanwhile, secoxyloganin was revealed at *m/z* 403.12. The MS/MS fragmentation patterns of these compounds are elucidated in Table S1.

##### Oleuropein-type secoiridoid and derivatives

Among the 39 compounds in the secoiridoid class, 23 have been tentatively identified as oleuropein-type secoiridoids (Fig. S4 B), showcasing the crucial role of these compounds in olive extract, which influences human health. Among these compounds is oleuropein, a key component in olives, showing the most prevalent peak (Fig. 1). Oleuropein consists of hydroxytyrosol (precursor) linked to elenolic acid glucoside by an ester bond, so termed “hydroxytyrosol-related secoiridoids”^56^.

Oleuropein was identified at *m/z* 539.18 at 8.07 min, showing fragmentation corresponding to characteristic losses such as methyl ester moiety (32.02 Da), C_4_H_6_O (70.04 Da), and hexosyl residue (162.05 Da) at *m/z* 507.15, 469.13, and 377.12, respectively. Additionally, from ion *m/z* 403.12 [elenolic acid hexoside-H]^−^, daughter ions were obtained at *m/z* 371.10, 223.06, 179.06, and 149.02. From ion *m/z* 377.12 [M-H-Glc]^−^, fragment ions were generated at *m/z* 359.12, 345.10, and 307.08, followed by ions at *m/z* 327.09 and 275.09. Besides, the fragment ions at *m/z* 153.06 and 123.04 were detected due to hydroxytyrosol’s cleavage of oleuropein (Refer to Table S1 for fragment details)^57,58^.

Whereas, methoxyoleuropein was detected at *m/z* 569.19, which eluted before oleuropein based on literature^49^. Lucidumoside C was also present at *m/z* 583.20 as deprotonated formyl adduct [M-H + HCOOH]^−^ at *m/z* 629.19.

Besides, oleuropeinic acid (*m/z* 569.15), oleuropein hexoside (*m/z* 701.23 at 7.12 min), and oleuropein aglycone 3,4-DHPEA-EA) isomers (*m/z* 377.12 at 9.92 and 11.57 min) were detected, the latter also observed as a trihydrate adduct [M-H + 3H_2_O]^−^ at *m/z* 431.11. Hydroxyoleuropein showed deprotonated molecular ions at *m/z* 555.17, its aglycone was observed (*m/z* 393.12) (**Table****S1**).

Moreover, hydro-oleuropein, dihydro-oleuropein, jaspolyoside, demethyl oleuropein, methyl oleuropein aglycone, and hydroxy-*O*-decarboxymethyl oleuropein aglycone were also identified in OSE (**Table****S1**). The molecular ion *m/z* 405.16 was identified as dimethyl oleuropein aglycone, and finally, at *m/z* 705.28 molecular ion was assigned as oleuropein derivative 2^46,49^.

In addition, fraxamoside, previously detected in olive wood and olive oil, was exhibited as [M-H + FA]^−^ at *m/z* 583.17 and [M + Cl]^−^ at *m/z* 573.14, showing a base peak at *m/z* 537.16 [M-H]^−^. Their MS^2^ spectra revealed 6 fragments’ ions at *m/z* 403.12 [M-H-C_8_H_6_O_2_]^−^ (C_17_H_23_O_11_) attributed to the fragment of elenolic acid glucoside, followed by carbonyl loss (CO; 28 Da) at *m/z* 375.11, with two fragments representing loss of hexosyl moiety plus water (180.07 Da) and subsequent decarboxylation (43.99 Da) from *m/z* 403.12 at *m/z* 223.0613 (C_11_H_11_O_5_) and 179.0572 (C_10_H_11_O_3_), as well *m/z* 151.0400, and 123.0456 attributed to fragments related to hydroxytyrosol^11,44,49^.

##### Ligstroside-type secoiridoid derivatives

Ligstroside, a secoiridoid compound in *O. europaea*^59^, is structurally similar to oleuropein with tyrosol linked to oleoside 11-methyl ester instead of hydroxytyrosol by an ester bond and a sugar unit attached to C-1 position^11^. Tyrosol is considered a precursor for ligstroside, classifying it as a “tyrosol-related secoiridoid” (**Fig. S5 A**).

Several tyrosol-related secoiridoid compounds have been tentatively identified in OSE. These are ligstroside (*m/z* 523.18), ligstroside glucoside (*m/z* 685.23; base peak), ligstroside aglycone (*m/z* 361.13) exhibited as [M + HCOOH-H]^−^ at *m/z* 407.14, demethyl ligstroside (*m/z* 509.17), and jaspolyoside (*m/z* 909.30) Table S1.

The study opted to depict potentially a new compound in OSE by examining MS and MS^2^ spectra, showing a precursor ion at *m/z* 553.15 of C_25_H_30_O_14_ that was tentatively identified as ligstrosidic acid. The peak at *m/z* 521.12 is due to demethoxylation, and fragment ions included *m/z* 433.09 (loss of dehydroxytyrosol), 209.04 (loss of tyrosol, hexosyl moiety, and CHO_2_), and 167.04 (successive loss of C_2_H_2_O). The fragment at *m/z* 391.10 indicated the loss of a glucosyl moiety, followed by *m/z* 373.09 (loss of H_2_O), *m/z* 313.07 (loss of 2CH_2_O), *m/z* 289.07 (loss of 2C_2_H_2_O), and other fragments mentioned in Table S1 that were compared to those described in^21,60^ and shown in Fig. 2.

##### Nuzhenide-type secoiridoids

Nuzhenide and neo-nuzhenide, secoiridoid in olive organs, feature a sugar unit between elenolic acid glucoside and either tyrosol or hydroxytyrosol molecule, respectively (Fig. S5 B). They were identified at *m/z* 685.23 and 701.23, respectively, exhibiting similar losses in the MS^2^ spectrum with additional hydroxyl group in neo-nuzhenide Table S1.

##### Other secoiridoids

In OSE, acyclic secoiridoids (Fig. S5 C) were identified by negative ionization mode and named “other secoiridoids”.

Acyclodihydroelenolic acid hexoside was identified at *m/z* 407.16, while ethyl-hydroxy-propionyl cyclohexyl) acetic acid hexoside at *m/z* 403.20, showing different fragments (Table S1).

Interestingly, an additional ion at *m/z* 213.08 (nuzhenal A, C_10_H_14_O_5_) was detected, potentially a new molecule in olive organs, structurally close to elenolic acid. Nuzhenal A is an acyclic mono-aldehydic form of elenolic acid decarbonylation. Its fragmentation pattern showed ions at *m/z* 195.06[M-H-H_2_O]^−^, 183.07 [M-H-CH_2_O]^−^, 165.05 [M-H-CH_2_O-H_2_O]^−^, 150.03 (base peak) [M-H-CH2O-H_2_O-CH_3_]^−^, 122.04 [M-H-CH_2_O-H_2_O-CH_3_-CO]^−^, and 107.08 [M-H-CH_2_O-H_2_O-2CH_3_-CO]^−^, differing from elenolic acid by having one less carbonyl group (CO; 27.99 Da) (Fig. 2). These fragments are comparable to those described in^60^.

#### Lignans, glycosides, and derivatives

Lignan compounds, an important class in olive trees, are mainly found in the stem and roots, separated from the bark and wood. Lignans are dimeric phenylpropanoid structures joined by an 8,8’- or *β*, *β*’-link, forming two (C6-C3)_2_ units of phenylpropanoid as shown in Fig. S6^61,62^. Based on the ring linked to the dimeric structures of phenylpropanoid molecules, they are classified into 3 subclasses: furan ring (olivil-based lignan subclass), cyclohexane (cyclolivil-based), and furofuran (pinoresinol-based). Lignans offer numerous health benefits, including antiviral, anti-inflammatory, antioxidant, and anti-estrogenic properties. They may also help prevent osteoporosis, reduce cancer risk, manage menopausal symptoms, and support cardiovascular health^63–65^.

(−)-Olivil/(+)-cycloolivil, a compound with a furan ring Fig. S6 A, as its deprotonated ion at *m/z* 375.14. Additionally, olivil/cycloolivil hexosides were detected at *m/z* 537.20, with a base peak at *m/z* 375.14, followed by a similar fragmentation pattern of olivil/cycloolivil (Table S1**)**. Cycloolivil hexoside (Rt 5.18 min) eluted before olivil hexoside (Rt 6.33 min) as in^49^.

Pinoresinol-based lignan compounds Fig. S6 C, the major group in virgin olive oil, are associated with remarkable health benefits due to high oxidative stability^66^. Two isomers of Hydroxypinoresinol hexoside were detected *m/z* 535.18, and one of them in adduct form [M + FA-H]^−^ at *m/z* 581.19, base peak at *m/z* 357.13. Alongside, pinoresinol hexoside was detected at *m/z* 519.19, base peak at *m/z* 357.13. Besides, hydroxypinoresinol (*m/z* 373.13) showing one more hydroxyl group than detected pinoresinol. Their MS/MS spectra showed base peak fragment ions at *m/z* 343.12 and 327.12, indicating CH_2_O losses^67^ Table S1**.**

LC-MS/MS identified two acetoxypinoresinol hexoside isomers at *m/z* 577.19 [M-H]^−^; isomer 1 as [M + FA-H]^−^ at *m/z* 623.20, with major fragment ion at *m/z* 415.14 (-hexosyl). While fraxiresinol hexoside was determined at [M-H]^−^ 565.19 *m/z* and syringaresinol 417.15 *m/z*, Table S1.

This study identified four potentially new furofuranoid lignans in OSE derived from syringaresinol. Firstly, syringaresinol hexoside (C_28_H_35_O_13_) was found with a precursor ion at *m/z* 579.20. Its MS^2^ spectrum revealed various fragment ions indicating losses of 2 and 3 water molecules (543.21 and 525.20 *m/z*), C_2_H_5_OH (533.16 *m/z*), and hexose (417.16 *m/z*) (Fig. 2)^68^. Secondly, eleutheroside E (C_34_H_46_O_18_) exhibited a deprotonated formyl adduct at *m/z* 787.27 with a molecular ion at *m/z* 741.26, hexosyl losses at *m/z* 579.21 and 417.16, and C_14_H_17_O_4_ loss at *m/z* 181.05 giving [syringaldehyde-H]^−^ in MS/MS spectrum (Fig. 2)^69^. Thirdly, hydroxysyringaresinol (C_22_H_26_O_9_) showed a precursor ion at *m/z* 433.15, with a fragmentation pattern similar to syringaresinol aglycone but with an additional hydroxyl group (Fig. 2). These findings enhance the understanding of the chemical composition of OSE. Finally, buddlenol D (C_33_H_40_O_13_), a trimeric phenylpropanoid, showed a precursor ion at *m/z* 643.24 [M-H]^−^ (Fig. 2). Based on previous literature, the MS/MS fragmentation pattern showed ions *m/z* 595.22 [M-H-CH_2_O-H_2_O]^−^, 417.15 [syringaresinol-H]^−^, 387.14 [syringaresinol-H-CH_2_O]^−^, 225.08 [M-H-C_22_H_26_O_8_]^−^, indicating the cleavage of syringaresinol (Fig. S6 C), Table S1.

#### Hydroxycoumarins and derivatives

Hydroxycoumarin has two adjacent hydrogen atoms on a benzene ring replaced by an unsaturated lactone ring, forming a six-membered heterocycle attached to a carbonyl group. Its general formula is C_9_H_6_O_3_ (162.03); as shown in Fig. S7^70^.

Aesculetin deprotpnated ion peak (*m/z* 177.02) and its glycoside aesculin (*m/z* 339.07) (Fig. S7) were identified in OSE. Additionally, scopoletin was detected at *m/z* 191.03, exhibiting major fragment ion at *m/z* 176.01 Table S1.

We elected to depict umbelliferone (Hydroxycoumarin; C_9_H_6_O_3_) as a new compound in OSE in this study, which was tentatively identified at Rt 6.61 min *m/z* 161.02 [M-H]^−^. The MS^2^ spectrum revealed the fragment ions at 133.03 [M-H-CO]^−^ followed by the subsequent losses of double and triple carbonyl groups (CO; 28 Da) at *m/z* 105.03 and 77.04, respectively. In addition, the peak ions at *m/z* 117.03 and 89.04 indicated the loss of CO_2_ followed by a subsequent loss of CO, respectively (Fig. 2 and Table S1); in agreement with those reported by^71^.

#### Flavonoid derivatives

Different subclasses were detected in OSE based on their basic skeleton and the type of group attached to one of the three rings (Fig. S3 D). Several flavonoid compounds were detected and tentatively identified by comparing their fragmentation patterns with those in databases and literature (**Fig. S8** and Table S1)^49^.

##### Flavanones

Three flavanone metabolites were identified in OSE (Fig. S8 A): naringenin (271.06 *m/z*) and its glycoside (433.11 *m/z*), and dihydroxy flavanone (pinocembrin) (255.07 *m/z*), Table S1.

##### Flavanonols

Dihydrokaempferol was identified at 287.06 *m/z*, eluting at 6.79 min. In addition, taxifolin (303.05 *m/z*) and its glycoside (465.11 *m/z*) were detected at Rt 6.03 min and 4.22 min, respectively (Fig. S8 B).

##### Flavones

Six flavone compounds were identified in OSE using - (Fig. S8 C) including; luteolin-7-*O*-hexoside with *m/z* 447.10 at Rt 6.75 min and fragment ion at *m/z* 285.04 upon hexosyl moiety loss. Luteolin was annotated at 285.04 *m/z*, and chrysoeriol-*O*-hexoside at 461.11 *m/z*. Additionally, apigenin-*O*-hexoside and its aglycone were found at *m/z* 431.10 and 269.05, respectively, while isorhoifolin was detected at 577.16 *m/z* exhibiting the major fragment ion at *m/z* 269.05 derived from the loss of hexosyl residue Table S1.

##### Flavonols

Using UPLC-HR-QTOF-MS, various flavonols were identified in OSE (Fig. S8 D). Quercetin was detected at 301.03 *m/z* (major peak) and quercetin hexoside (isoquercetrin) at 463.09 *m/z* (major peak), along with quercetin-3-*O*-rutinoside (rutin) exhibiting in two isomers with same molecular ion *m/z* 609.15 at Rt 5.97 and 6.04 min. Additionally, kaempferol-7-*O*-hexoside was revealedat *m/z* 447.09, showing a base peak at *m/z* 285.04 from the loss of hexosyl moiety. Full details are provided in Table S1.

#### Triterpenic acids

Triterpenes, an additional class found in OSE, were identified mainly by their precursor ions using Fig. S9.1. Dihydroxy-oxo-oleanolic acid (485.33 *m/z*) and hydroxy-oxo-oleanolic acid (469.33 *m/z*) were detected, along with asiatic acid (487.34 *m/z*). Additionally, 2-*α*-hydroxyursolic acid and maslinic acid (both 471.35 *m/z*) eluted at Rt 15.48 and 17.02 min, respectively, differ in the position of the CH_3_ group. Maslinic acid methyl ester (485.36 *m/z*) and ursolic acid and its isomer oleanolic acid (both 455.35 *m/z*) were also identified. Full details are provided in Table S1.

#### Fatty acids

Using UPLC-HR-QTOF-MS, several fatty acids were identified in OSE (Fig. S9.2), including palmitic acid (Fig. S9.2 A), which is SFA exhibited at 255.23 *m/z*. As well as, 2,3-dinor-8-iso-prostaglandin F1 alpha, 9,10,18-trihydroxyoctadecenoic acid, oleic acid, and hydroxy-octadecatrienoic acid (293.21 *m/z*) are examples of MUSFA (Fig. S9.2B), which were detected at *m/z* 327.22, 329.23, and 281.25, respectively. Also, hydroxy-octadecadienoic acid is PUSFA and was determined at *m/z* 295.23 (Fig. S9.2 C)Table S1.

#### Other compounds

The UPLC-HR-QTOF-MS analysis of olive extract identified aldehydic compounds, including vanillin hexoside in two isomers with similar molecular ions (313.09 *m/z*), vanillin (151.04 *m/z*), syringaldehyde (181.05 *m/z*), and sinapaldehyde (207.07 *m/z*) (Fig. S10 A). While 2-phenylethyl *β*-primeveroside (Fig. S10 B) is phenylethyl diglycosidic compound, it was detected at *m/z* 415.16. In addition, malic acid and quinic acid (Fig. S10 C) were identified at *m/z* 133.01 and 191.06, respectively. Full fragment details are described in Table S1.

### Antiviral activity in vitro against viral antigens

Olive stem extracts were screened for antiviral activity against three viral human pathogens, HSV-1, CB-4, and adeno-7, and tested for anti-replicative (treatment) and direct virucidal (neutralization).

**Fig. 3:**
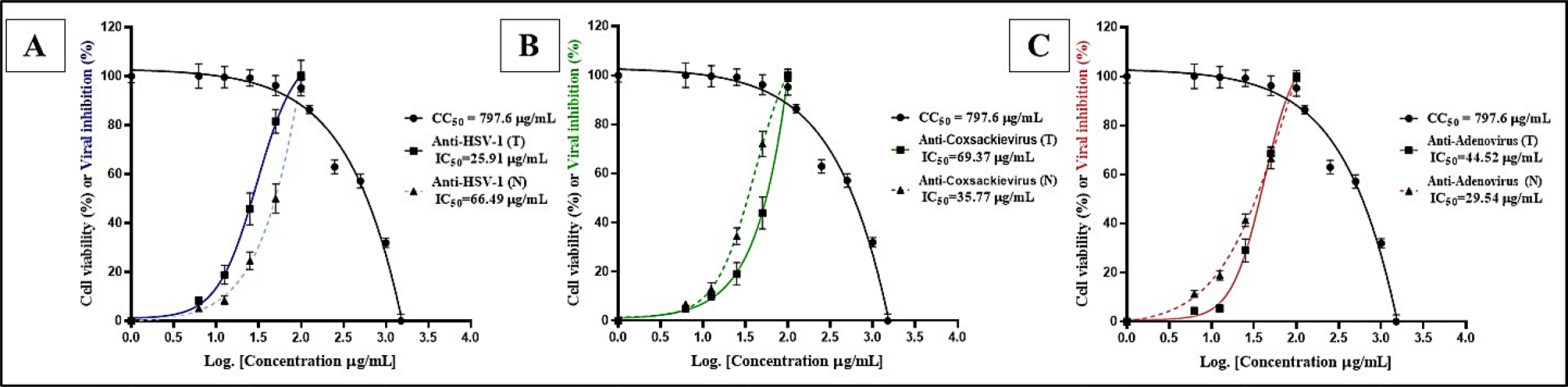
Graphical presentation of the cell viability and antiviral activity of the Olive stem extract by using the *Vero* cell line, against HSV-1 (A), coxsackievirus (B), and adenovirus (C).

**Fig. 4:**
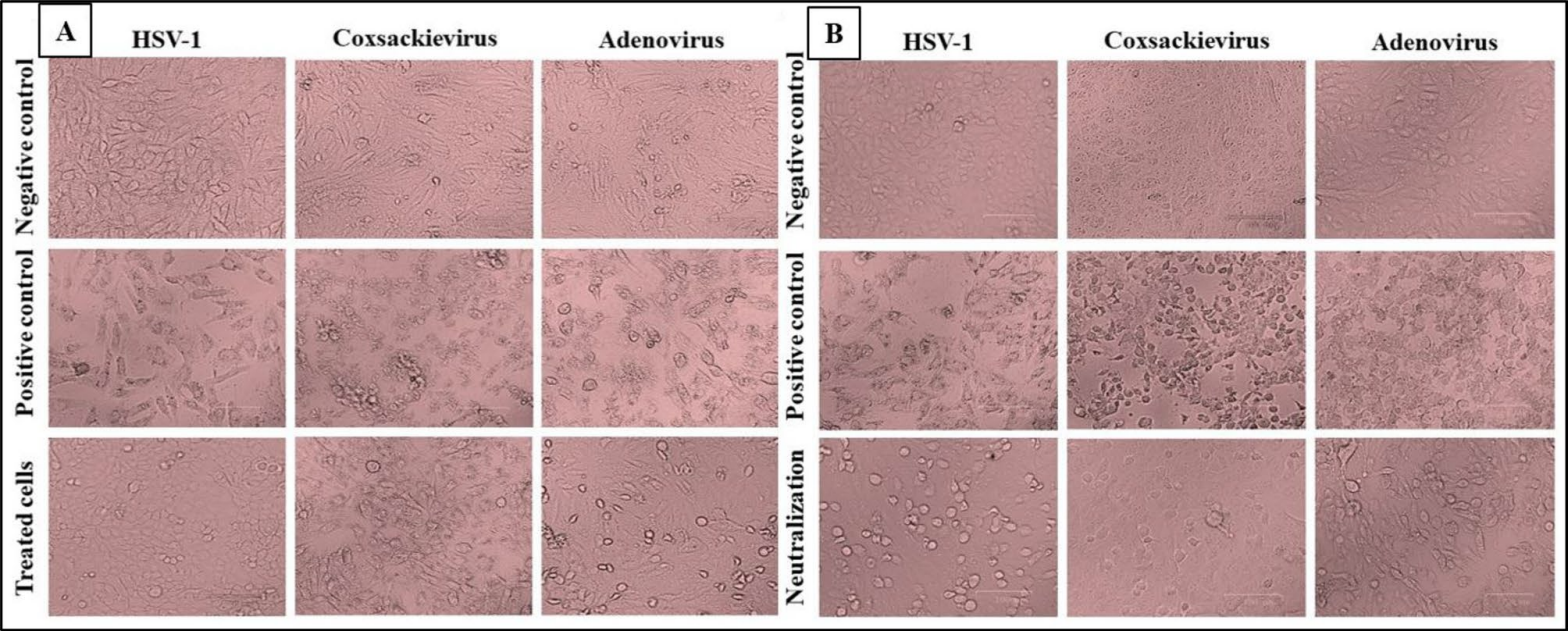
In vitro anti-cytopathic effect of extract against HSV-1, coxsackievirus, and adenovirus infection in the *Vero* cell lines. showing the treatment mechanism (A) and the neutralization mechanism (B).

The in vitro antiviral potential of olive stem extract (OSE) was assessed against HSV-1, coxsackievirus, and adenovirus using *Vero* cell lines, with acyclovir as a reference antiviral drug. Cytotoxicity and antiviral efficacy were evaluated by determining the half-maximal cytotoxic concentration (CC_50_) and inhibitory concentration (IC_50_), demonstrating that OSE is safe, with a CC_50_ of 797.6 ± 1.24 µg/mL and an EC_100_ of 131.71 ± 2.12 µg/mL (Table 1; Fig. 3). Different mechanisms were tested to assess the extract’s antiviral activity, revealing that the treatment mechanism exhibited the highest efficacy against HSV-1, with a selectivity index (SI) of 30.78, followed by the neutralization mechanism against adenovirus (SI = 27) and coxsackievirus (SI = 22.3). The absence of a blocking effect suggests that OSE does not act through receptor binding but instead inhibits viral replication via neutralization or enzymatic inhibition (Fig. 4).

**Table 1:** Cell viability and antiviral activity of the Olive stem extract against HSV-1, coxsackievirus, and adenovirus with their cytotoxic concentration (CC_50_), half-maximal inhibitory concentration (IC_50_), and selectivity index (SI) under both treatment and neutralization effect.

	Conc.	Anti-HSV-1		Anti-Coxsackievirus		Anti-Adenovirus	
		**Treatment**	**Neutralization**	**Treatment**	**Neutralization**	**Treatment**	**Neutralization**
olive stem extract	IC_50_ (µg/mL)	25.91 ± 1.02	66.49 ± 1.01	69.37 ± 2.64	35.77 ± 0.05	44.52 ± 2.06	29.54 ± 3.27
	SI*	30.78	11.99	11.5	22.3	17.91	27
Acyclovir	IC_50_ (µg/mL)	3.01 ± 0.99	–	–	–	15.88 ± 1.63	–
	SI*	24.05	–	–	–	4.55	–

IC_50_ Half maximal inhibitory concentration; SI*= selectivity index (CC_50_/IC_50_), CC_50_ of acyclovir on Vero cell line = 72.381 µg/mL.

Compared to previous studies, our findings highlight the superior selectivity and efficacy of OSE against HSV-1. The ethyl acetate fraction from olive leaves previously exhibited a CC_50_ of 610 µg/mL and an IC_50_ of 40 µg/mL, resulting in an SI of 15.2, which improved to 16.9 when formulated as a microemulsion^72^. This SI is significantly lower than that observed in our study SI of 30.78, indicating OSE possesses enhanced antiviral potency relative to previously investigated olive leaf extracts. Additionally, other studies assessing olive-derived extracts *O.europaea* var. *sativa* (OESA) and *O.europaea* var. *sylvestris* (OESY) reported SI values of 17.7 and 4.98, respectively, further reinforcing the superior efficacy demonstrated by OSE in our research^73^. In pre- and post-infection assays, OESA exhibited SI values ranging from 1.3 to 1.6, whereas OESY showed SI values of 4.1 to 7.4, all considerably lower than the selectivity index recorded for OSE in this study^74^.

These comparative findings emphasize the promising potential of OSE as a potent antiviral candidate, displaying higher efficacy and selectivity than previously studied olive-derived extracts in preventing the replication of both HSV-1 and adenovirus. This antiviral activity was evaluated using the MTT assay and ELISA kits to measure the inhibition of viral antigen levels. Results from the former test, Table 2, indicate that the OSE reduced viral antigen levels in the supernatant by 94.80% and 82.32% and in the cells by 84.23% and 72.98% for HSV-1 and adenovirus, respectively, compared to cells infected with these viruses alone and without treatment. This suggests the compound can inhibit viral release from infected cells and reduce virus transmission via cell-to-cell contact. Further detailed studies and in vivo experimental models are necessary to confirm and elucidate the definitive antiviral mechanism of this compound.

**Table 2:** Effect of the Olive stem extract on the viral antigen level.

% Inhibition of viral antigen	HSV-1	HAdV-Ag
Supernatant	94.80% ± 3.73	82.32% ± 2.43
Cells	84.23% ± 2.08	72.98% ± 1.92

HAdV-Ag (Human adenovirus antigen).

### Network Pharmacology analysis

Network pharmacology is a powerful approach for the investigation of drug targets. In current research, compounds from olive stems were found to interact with multiple targets, often showing a synergistic effect. The pharmacology network was established on the five major compounds, as shown in the chromatogram Fig. S11, selected primarily by peak area. Notably, these compounds also exhibited high peak intensity, reflecting a natural correlation between both parameters. In this study, the olive stem extract demonstrated promising results against herpes simplex 1 virus, outperforming other viruses tested.

#### Construction of compound targets and gene targets

The selected 5 bioactive components of the olive stem: oleuropein, lucidumoside C, hydroxyoleuropein, oleuropein aglycone, and acetoxypinoresinol hexoside were searched for the target proteins and their corresponding gene names in the UniProt database. HSV-1 gene targets were retrieved from GeneCards database. Initially, 1677 genes were obtained, reduced to 302 genes after applying GIFs filter of < 55. By overlapping the targets of the compounds with the gene targets using a Venn diagram, they identified eight key anti-HSV-1 targets from the olive stem (Fig. S12).

By inputting 8 anti-HSV-1 targets into the STRING database, resulting in a protein interaction network with eight nodes: NFKB1, CHUK, PIK3R1, TLR4, NFE2L2, TOP2A, CAPN1, and NOS2 This network showed 21 edges and a protein-protein interaction^39^ enrichment *p*-value of 0.0176, indicating significant interactions, particularly among NFKB1, CHUK, and TLR4. Each node represented a gene, and the connections between nodes indicated their interactions (Fig. S13). A node’s degree reflected the influence of the target within the network, affecting the ranking of targets or PPI.

**Fig. 5:**
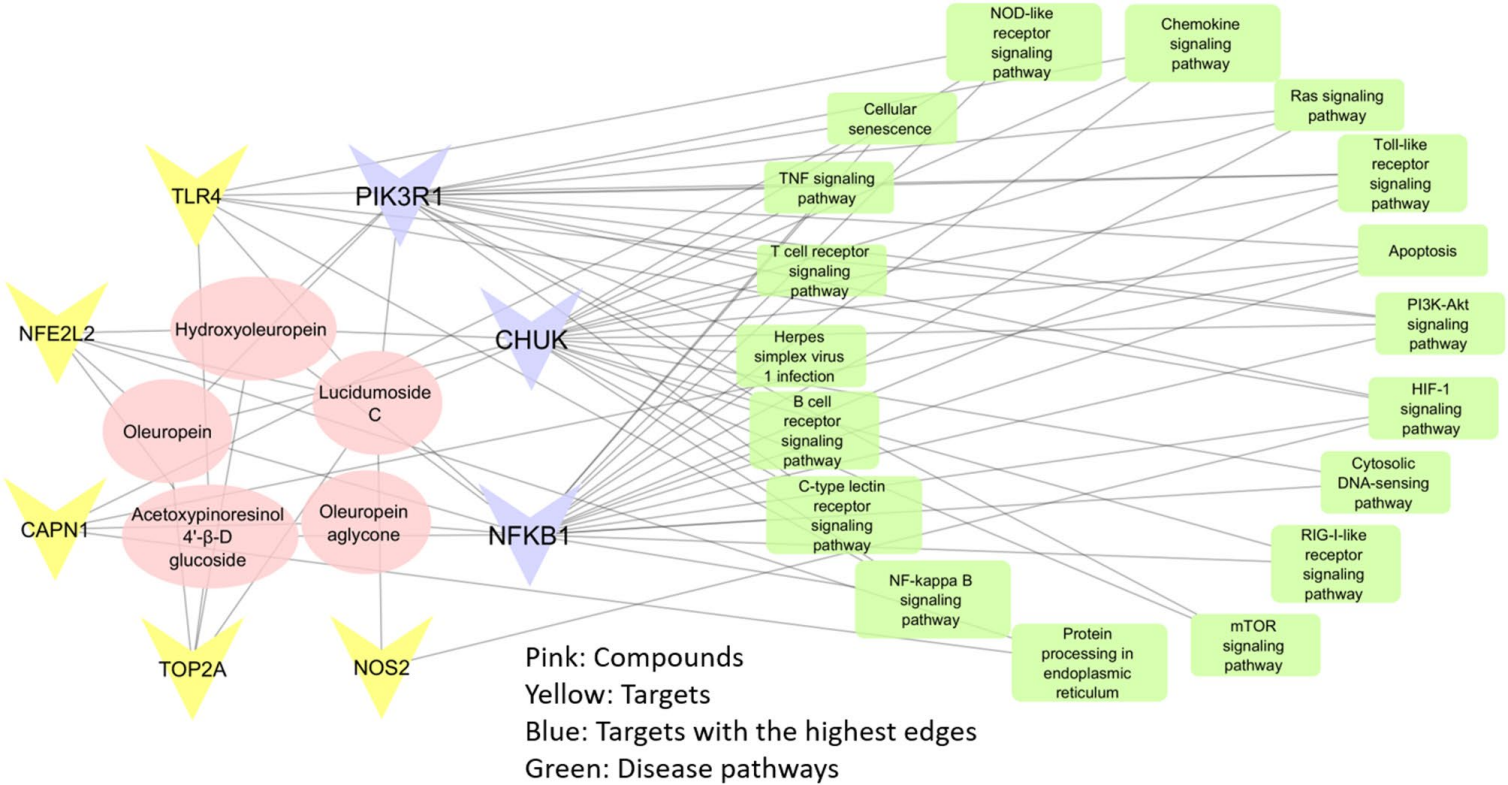
Compound-target-pathway network analysis of olive stem.

KEGG pathway analysis was performed on the 8 key targets to clarify how olive stem combats HSV-1. A total of 73 KEGG pathways were obtained, filtered down to 18 KEGG pathways that were specific to HSV-1. These include Toll-like receptor signaling, apoptosis, PI3K-Akt, NF-kappa B signaling, and several others (see Fig. 5; Table 3). These signaling pathways’ interactions may synergize to contribute to the medicinal effects of olive stem extract against HSV-1.

**Table 3:** The 5 core compounds of the Olive stem, 8 core targets, and 18 core pathways in HSV-1 disease.

Classification	No.	Name	Degree	Closeness Centrality	Betweenness Centrality
Core components	1	Lucidumoside C	7	0.55555556	0.09695607
	2	Oleuropein	5	0.51724138	0.03881092
	3	Hydroxyoleuropein	5	0.51724138	0.03881092
	4	Acetoxypinoresinol 4’-*β*-D hexoside	4	0.48387097	0.0238519
	5	Oleuropein aglycone	2	0.45454545	0.01224555
Core targets	1	NFKB1	21	0.73170732	0.37456637
	2	CHUK	18	0.63829787	0.21250002
	3	PIK3R1	16	0.58823529	0.17261438
	4	TLR4	7	0.42253521	0.02979197
	5	NFE2L2	5	0.4109589	0.05082137
	6	TOP2A	4	0.38961039	0.00576873
	7	CAPN1	4	0.37037037	0.02625051
	8	NOS2	2	0.37037037	0.00124987
Core pathways	1	Toll-like receptor signaling pathway	4	0.48387097	0.01048489
	2	Apoptosis	4	0.5	0.03812703
	3	PI3K-Akt signaling pathway	4	0.48387097	0.01048489
	4	HIF-1 signaling pathway	4	0.48387097	0.02874942
	5	NF-kappa B signaling pathway	3	0.46875	0.00620885
	6	Herpes simplex virus 1 infection	3	0.46875	0.00211514
	7	B cell receptor signaling pathway	3	0.46875	0.00211514
	8	C-type lectin receptor signaling pathway	3	0.46875	0.00211514
	9	T cell receptor signaling pathway	3	0.46875	0.00211514
	10	TNF signaling pathway	3	0.46875	0.00211514
	11	Cellular senescence	3	0.48387097	0.0231587
	12	NOD-like receptor signaling pathway	3	0.46875	0.00620885
	13	Chemokine signaling pathway	3	0.46875	0.00211514
	14	Ras signaling pathway	3	0.46875	0.00211514
	15	Cytosolic DNA-sensing pathway	2	0.45454545	6.60E-04
	16	RIG-I-like receptor signaling pathway	2	0.45454545	6.60E-04
	17	mTOR signaling pathway	2	0.42857143	5.96E-04
	18	Protein processing in endoplasmic reticulum	2	0.31914894	0.005502

#### Analyses of the PPI network

Using Cytoscape 3.7.2, the results were constructed as a network to illustrate the interactions between anti-HSV-1 bioactive compounds and their targets. This network included 31 nodes: 5 bioactive compounds, 8 gene/protein targets, and 18 pathways, connected by 154 edges, as depicted in Fig. 5; Table 3. Key proteins like NFkB, CHUK, and PIK3R1 were highlighted by blue color due to their high degree of interaction (21, 18, and 16, respectively) as shown in Table 3, suggesting their pivotal roles in cellular responses to pharmacological interventions. The network demonstrated how multiple compounds from the olive stem interact with various targets and pathways, emphasizing the complex mechanism behind its anti-HSV-1 properties. For example, lucidumoside C interacted with 7 targets (NFKB1, NFE2L2, TOP2A, NOS2, TLR4, PIK3R1, and CHUK), oleuropein with 5 (NFKB1, NFE2L2, TOP2A, CHUK, and PIK3R1), and acetoxypinoresinol- hexoside with 4, (NFKB1, NFE2L2, TOP2A, and TLR4). Among them, they commonly acted on 3 targets (NFKB1, NFE2L2, and TOP2A), while both lucidumoside C and acetoxypinoresinol 4’-β-D hexoside acted on 1 target (TLR4); however, lucidumoside C could be distinguished by 1 target (NOS2) from the rest of the bioactive compounds.

## Conclusion

This article focused on olive stems, abundant due to the annual pruning of olive trees. Using a robust analytical method, 119 compounds in olive stem extract (OSE), including new compounds across 11 structural classes, were identified. These included sugar derivatives (5), phenolic acids (6), phenylethanoids (9), iridoid glycosides (3), secoiridoids (with oleuropein as the major compound) (39), lignans (16), hydroxycoumarins (4), flavonoids (16), terpenes (8), fatty acids (6), and other compounds (7). The study utilized UPLC-HR-QTOF-MS in negative ionization mode to analyze the 70% ethanol extract of the olive stem. This comprehensive analysis provides a valuable reference for further research on olive stem extract (OSE).

Moreover, this article highlights the cytotoxic effects of OSE on the *Vero* cell and its promising potential as a potent antiviral candidate, demonstrating higher efficacy and selectivity than previously studied olive-derived extracts against herpes simplex virus type-1 (HSV-1), coxsackievirus type-B4 (CB-4), and adenovirus type-7 (Adeno-7). Utilizing the MTT assay, the study confirmed OSE’s ability to inhibit HSV-1 replication through treatment and neutralize adenovirus via direct virucidal effect, with IC_50_ values of 25.91 ± 1.02 µg/mL and 29.54 ± 3.27 µg/mL, respectively. The selectivity indices (SI) for HSV-1 and adenovirus were recorded at 30.78 and 27, indicating strong antiviral potential. Additionally, OSE exhibited significant safety, with a CC_50_ (cytotoxic concentration) of 797.6 ± 1.24 µg/mL and an EC_100_ (effective concentration) of 131.71 ± 2.12 µg/mL in the *Vero* cell.

In addition, various tools played a crucial role in the network pharmacology assessment of OSE for HSV-1. Analyzing the top 5 intense peak ions, prioritizing those with the highest peak area, in relation to HSV-1 disease, yielded a promising result in the in vitro assay, 8 anti-HSV-1 prediction targets across 18 pathways, leading to the construction of a compound-target-pathway network. Among the key targets, NFkB, CHUK, and PIK3R1 were highlighted for their significant roles in cellular responses to pharmacological interventions, owing to their high degree of interaction within the network. Furthermore, the study pinpointed five important pathways: Toll-like receptor signaling, apoptosis, PI3K-Akt signaling, HIF-1 signaling, and B cell receptor signaling. These pathways likely contribute to the olive stem’s potential treatment against HSV-1.

These findings shed light on the potential of using olive stem waste from tree pruning as a valuable antiviral agent, especially for preventing HSV-1 replication (treatment) and neutralizing adenovirus (direct virucidal) that support the traditional use of olive in treating viral accompanied diseases like asthma, sore throat, fever, and infectious diseases. Additionally, this research suggests that olive stem waste could be feasible for animal feed as zoonotic agents and beneficial for human health as a cost-effective pharmaceutical product. These efforts align with the 2030 UN Sustainable Development Goals, particularly SDG-12 for responsible consumption and production, and SDG-3 for health and well-being. To ensure safe use in both human and animal feed, it is essential to establish formulations and standardized doses.

## Electronic supplementary material

Below is the link to the electronic supplementary material.

Supplementary Material 1

## Data Availability

“Data is provided within the manuscript or supplementary information files”.

## Abbreviations

ACN: Acetonitrile
Adeno-7: Adenovirus type 7
AI: Artificial Intelligence
ATCC: American Type Culture Collection
BPC: Base Peak Chromatogram
CB-4: Coxsackievirus type B4
CC_50_: Half-maximal cytotoxic concentration
CHUK: Conserved Helix-Loop-Helix Ubiquitous Kinase
DALYS: Disability– Adjusted Life Years
DHBA: Dihydroxybenzoic acid
DHPEA: Dihydroxyphenyl ethanol (hydroxytyrosol)
DHPEA-EA: Dihydroxyphenylethanol elenolic acid (oleuropein aglycone)
DHPG: Dihydroxyphenylglycol
DHPG: Dihydroxyphenylglycol
DIA: Data-independent acquisition
DMEM: Dulbecco’s Modified Eagle’s Medium
DMSO: Dimethyl sulfoxide
EC_100_: Effective concentration for 100% viability
EIC: Extracted ion chromatogram
ELISA: Enzyme-linked immunosorbent assay
ESI mode: Electrospray ionization mode
FA: Formic acid
FooDB: Food Database
Glc.: Glucosyl
HAdV-Ag: Human adenovirus antigen
HIV: Human Immunodeficiency Virus
HPEA: Hydroxyphenyl ethanol (tyrosol)
HPE-EA: Hydroxyphenylethanol elenolic acid (ligstroside aglycone)
HSV-1: Herpes simplex virus type 1
HTyr: Hydroxytyrosol
IC_50_: Half maximal inhibitory concentration
KEGG pathway: Kyoto Encyclopedia of Genes and Genomes pathway
LC/MS: Liquid chromatography-mass spectrometer
LMWPs: Low molecular weight phenolics
MeOH: Methanol
MS: Mass spectrometry
MTT: 3-(4,5-dimethylthiazol-2-yl)-2,5-diphenyltetrazolium bromide
MUSFA: Monounsaturated fatty acids
NaOH: Sodium hydroxide
NFkB: Nuclear Factor kappa-light-chain-enhancer of activated B cells
O. europaea: Olea europaea
O.D.: Optical density
OSE: Olive stem extract
PBS: Phosphate-buffered saline
PDB: Protein data bank
Pent.: Pentoside
PIK3R1: Phosphoinositide-3-Kinase Regulatory Subunit 1
PPI: Protein-protein interaction
ppm: Part per million
psi: Pound per square inch
PUFA: Polyunsaturated fatty acids
Rham.: Rhamnosyl
rpm: Rotation per minute
Rt: Retention time
SDG: Sustainable Development Goals
SFA: Saturated fatty acids
SI: Selectivity index
TCID50: 50% tissue culture infectious dose
TIC: Total ion chromatogram
TriHOME: Trihydroxyoctadecenoic acid
UNSDGs: United Nations Sustainable Development Goals
U.S.: United State
UPLC-HR-QTOF-MS: Ultra-performance liquid chromatography coupled to a high-resolution quadrupole time of flight mass spectrometer
