# Supplementary material for: Network pharmacology of olive stem extract, UPLC-HR-QTOF-MS profiling and antiviral activities aligned with UN sustainable development goals
Source: Sci Rep. 2025 Jul 2;15:23665. doi: 10.1038/s41598-025-07452-1 (PMC12222765; doi:10.1038/s41598-025-07452-1)
Supplement: Supplementary file 1 — Supplementary Material 1 [file 41598_2025_7452_MOESM1_ESM.pdf]

The supplementary file of **"Network Pharmacology of Olive Stem Extract, UPLC-HR-QTOF-MS Profiling and Antiviral Activities Aligned with UN Sustainable Development Goals"**

Yasmin Mounir Mohamaden [a], Seham S. El-Hawary [a, *], Esmail M. El-Fakharany [b, c], Yousra A. El-Maradny [c, d], Mohamed El Raey [e], Amira Safwat El Senousy [a, #], Samar M. Bassam[*, f, #]

Table S1. List of tentative identification of (119) compounds extracted from ethanolic extract of olive stems using UPLC-HR-QTOF-MS/MS detection in negative ionization mode

NO.	Identification	Molecular formula	Exact mass	RT (min)	$[M-H]^-$ (Theo.) *m/z*	$[M-H]^-$ (Exp.) *m/z*	Error * (ppm)	MS/MS fragments (*m/z*) negative mode	References of negative mode
				**1- Sugars and derivatives**					
**1**	Gluconic acid / galactonic acid	$C_6H_{12}O_7$	196.05831	1.11	195.0505	195.0511	3.42	177.0407 $[M-H-H_2O]^-$, 159.0294 $[M-H-2H_2O]^-$, 129.0193 $[M-H-2H_2O-CH_2O]^-$, 111.0082 $[M-H-3H_2O-CH_2O]^-$, 101.0240 $[M-H-2H_2O-CH_2O-CO_2]^-$, 99.0087, 75.0086, 59.0139	[1-3]
**2**	Threonic acid (threonate)	$C_4H_8O_5$	136.03718	1.15	135.0294	135.0294	0.62	117.0096 $[M-H-H_2O]^-$, 89.0236 $[M-H-HCOOH]^-$, 75.0094 $[M-H-HCOOH-CH_2]^-$	[4]
**3**	Sucrose / disaccharide	$C_{12}H_{22}O_{11}$	342.11622	1.20	341.1084	341.1087	0.91	179.0561 $[M-H- Glc]^-$, 119.0351 $[M-H-Glc-H_2O-C_2H_2O]^-$, 89.0248 $[M-H-C_9H_{16}O_8]^-$, 71.0146 $[M-H-C_9H_{16}O_8-H_2O]^-$	[5-8]
**4**	Mannitol / sorbitol	$C_6H_{14}O_6$	182.07904	1.26	181.0712	181.0712	0.03	163.0619 $[M-H-H_2O]^-$, 149.0463, 131.0359, 119.0354, 101.0245 $[M-H-H_2O-C_2H_6O_2]^-$, 89.0246 $[M-H-H_2O-C_3H_6O_2]^-$, 85.0296, 71.0141 $[M-H-2H_2O-C_3H_6O_2]^-$, 59.0141 $[M-H-H_2O-C_4H_8O_3]^-$	[4,7], [2,9]
**5**	Glucotriose	$C_{18}H_{32}O_{16}$	504.16904	1.21	503.1612	503.1593	-3.81	341.1113 $[M-H-Glc]^-$, 323.0966 $[M-H-Glc-H_2O]^-$, 179.0565 (fragment of hexosyl residue) OR $[M-H-2Glc-H_2O]^-$, 89.0232 $[M-H-CO_2-H_2O-C_2H_4]^-$	[2,4,10]
				**2- Phenolic acids and derivatives**					
**1**	Vanilloyl-glucoside (vanillic acid glucoside)	$C_{14}H_{18}O_9$	330.09509	1.11	329.0873	329.0872	-0.18	195.0512 $[M-H-C_8H_6O_2]^-$, 167.0362 $[M-H-Glc]^-$, 152.0111 $[M-H-Glc-CH_3]^-$, 133.0146, 123.0455 $[M-H-Glc-CO_2]^-$, 108.0214 $[M-H-Glc-CO_2-CH_3]^-$	[1]
**2**	Vanillic acid	$C_8H_8O_4$	168.04226	1.36	167.0344	167.0343	-0.81	152.0115 $[M-H-CH_3]^-$, 123.0444 $[M-H-CHO_2]^-$, 108.0234 $[M-H-CO_2-CH_3]^-$	[1,11,12]
**3**	Protocatechuic acid glucoside (DHBA glucoside)	$C_{13}H_{16}O_9$	316.07944	1.24	315.0716	315.0727	3.53	195.0508, 153.0189 $[M-H-Glc]^-$, 152.0118 $[M-H-C_6H_{11}O_5]^-$, 133.0144 $[M-H-Glc-H_2O]^-$, 115.0036, 109.0286 $[M-H-Glc-CO_2]^-$, 108.0221 $[M-H-C_6H_{11}O_5-CO_2]^-$	[1,2,5]

NO.	Identification	Molecular formula	Exact mass	RT (min)	$[M-H]^-$ (Theo.) *m/z*	$[M-H]^-$ (Exp.) *m/z*	Error * (ppm)	MS/MS fragments (*m/z*) negative mode	References of negative mode
**4**	Dihydroxybenzoic acid hexoside pentoside	$C_{18}H_{24}O_{13}$	448.1217	1.27	447.1139	447.1143	0.87	429.0982 $[M-H-H_2O]^-$, 403.1225 $[M-H-CO_2]^-$, 315.0728 $[M-H-Pent.]^-$, 271.0857 $[M-H-Pent-CO_2]^-$, 153.0190 $[dihydroxybenzoic\ acid-H]^-$, 152.0115 $[M-H-Pent.-C_6H_{11}O_5]^-$, 109.0294 $[dihydroxybenzoic\ acid-H-CO_2]^-$, 108.0218 $[M-H-Pent.-C_6H_{11}O_5-CO_2]^-$	[2,13]
**5**	Protocatechuic acid (Dihydroxy-benzoic acid/DHBA)	$C_7H_6O_4$	154.02661	1.33	153.0188	153.0187	-0.56	109.0294 $[M-H-CO_2]^-$	[1,14-16]
**6**	Hydroxybenzoic acid/salicylic acid	$C_7H_6O_3$	138.0317	2.64	137.0239	137.0243	3.08	93.0352 $[M-H-CO_2]^-$, 75.0222 $[M-H-CO_2-H_2O]^-$, 65.0411 $[M-H-CO_2-CO]^-$	[1-3,17,18]
					**3- Phenylethanoids and derivatives**				
**1**	Dihydroxyphenyl-glycol (DHPG) / (dihydroxyphenyl-ethanediol)	$C_8H_{10}O_4$	170.05791	1.75	169.0501	169.0506	3.13	151.0400 $[M-H-H_2O]^-$, 123.0444 $[M-H-CH_2O_2]^-$	[1,19]
**2**	Hydroxytyrosol dihexoside	$C_{20}H_{30}O_{13}$	478.16864	1.98	477.1608	477.1614	1.23	315.1101 $[M-H-Glc]^-$, 153.0577 $[M-H-Glc-Glc]^-$ / $[hydroxytyrosol\ aglycone-H]^-$, 135.0459 $[hydroxytyrosol\ aglycone-H-H_2O]^-$, 123.0466 $[hydroxytyrosol\ aglycone-H-CH_2O_2]^-$	[1,16]
**3**	Hydroxytyrosol hexoside	$C_{14}H_{20}O_8$	316.11582	2.39	315.1080	315.1083	0.97	153.0556 $[M-H-hexoside]^-$, 135.0454 $[M-H-hexoside-H_2O]^-$, 123.0463 $[M-H-hexoside-CH_2O_2]^-$, 101.0245, 95.0127	[6,20], [1,2,8,21,22]
**4**	Hydroxytyrosol/ dihydroxyphenyl ethanol (HTyr/DHPEA)	$C_8H_{10}O_3$	154.063	3.10	153.0552	153.0556	2.81	135.0415 $[M-H-H_2O]^-$, 23.0451 $[M-H-CH_2O_2]^-$, 121.0308 $[M-H-CH_3OH]^-$, 109.0310 $[M-H-C_2H_4O]^-$, 108.0219, 95.0501	[1-3,11,13,16,22,23]
**5**	Tyrosol hexoside (Salidroside)	$C_{14}H_{20}O_7$	300.12091	3.41	299.1131	299.1141	3.33	137.0241 $[M-H-Glc]^-$, 119.0478 $[M-H-Glc-H_2O]^-$	[1,11,18,24,25]
**6**	hydroxyacteoside (Hydroxyverbascoside)	$C_{29}H_{36}O_{16}$	640.20034	5.90	639.1925	639.1937	1.85	621.1810 ($C_{29}H_{33}O_{15}$)/$[M-H-H_2O]^-$, 529.1555 $[M-H-C_6H_5O_2]^-$, 487.1475 $[M-H-C_8H_8O_3]^-$, 477.1317 $[M-H-caffeoyl]^-$, 459.1567 $[M-H-caffeoyl-H_2O]^-$, 323.0757 ($C_{12}H_{20}O_{10}$), 179.0356 $[caffeic\ acid-H]^-$, 161.0254, 135.0482 $[caffeic\ acid-H-CO_2]^-$	[2,5,8,26,27]
**7**	Verbascoside (acteoside/cinna-myl-caffeoyl phenylethanol glucoside)	$C_{29}H_{36}O_{15}$	624.20543	6.67	623.1976	623.1970	-0.96	555.1724, 461.1605 $[M-H-caffeoyl]^-$, 161.0252 $[caffeic\ acid-H-H_2O]^-$/$[glucose-H-H_2O]^-$, 151.0412 $[caffeic\ acid-H-CO]^-$ ($C_8H_7O_3$), 133.0294 $[caffeic\ acid-H-H_2O-CO]^-$	[2,4,27,28]

NO.	Identification	Molecular formula	Exact mass	RT (min)	[M-H]- (Theo.) *m/z*	[M-H]- (Exp.) *m/z*	Error * (ppm)	MS/MS fragments (*m/z*) negative mode	References of negative mode
**8**	Isoverbascoside	$C_{29}H_{36}O_{15}$	624.20543	6.80	623.1976	623.1983	1.12	555.1722, 461.1662 [M-H-caffeoyl]-, 161.0252 [caffeic acid-H-H_2O]-, 151.0413 [caffeic acid-H-CO]-, 133.0300 [caffeic acid-H-H_2O-CO]-	[2,4,27,28]
**9**	Hydroxytyrosol hexoside derivative	$C_{24}H_{34}O_{10}$	482.2152	7.69	481.2074	481.2083	2.01	315.1091 [hydroxytyrosol hexoside-H]-, 297.0977 ($C_{14}H_{17}O_7$), 153.0558 [free hydroxytyrosol-H]-, 135.0449 [hydroxytyrosol-H-H_2O]-, 123.0453 [M-H-hexoside-CH_2O_2]-	[8]
					**4- Iridoid glycosides**				
**1**	Loganic acid	$C_{16}H_{24}O_{10}$	376.13695	1.12	375.1291	375.1299	2.07	331.1433 [M-H-CO_2]-, 329.0898 [M-H-C_2H_5OH]-, 213.0768 [M-H-Glc]-, 195.0512 [M-H-Glc-H_2O]-, 169.0868 [M-H-Glc-CO_2]-, 151.0756 [M-H-Glc-CO_2-H_2O]-, 125.0610 [M-H-Glc-CO_2-C_2H_4O]-, 113.0258 (loss of $C_5H_8O_3$; 116.05Da), 107.0519	[1,2,5,6,16,29,30]
**2**	Loganin iosmer 1 (loganoside)	$C_{17}H_{26}O_{10}$	390.1526	1.69	389.1448	389.1437	-2.70	345.1560 [M-H-CO_2]-, 301.1658 [M-H-2CO_2]-, 227.0942 [M-H-Glc]-, 209.0538 [M-H-Glc-H_2O]-, 195.0658 [M-$_2$H-Glc-OCH_3]-, 183.0612 [M-H-Glc-C_2H_4O]-, 175.0609, 165.0594 [M-H-Glc-C_2H_4O-H_2O]- / [M-H-Glc-CO_2-H_2O]-, 161.0497, 151.07779 ($C_9H_{11}O_2$), 127.0485, 125.0975, 121.0677 [M-H-Glc-C_2H_4O-H_2O-CO_2]- / [M-H-Glc-$_2CO_2$-H_2O]-, 119.0359, 115.0415, 113.0247, 101.0264, 83.0139	[2,8,26], [3,16]
**3**	Loganin isomer2	$C_{17}H_{26}O_{10}$	390.1526	5.17	389.1448	389.1454	1.61	357.1176 [M-H-CH_3OH]-, 345.1552 [M-H-CO_2]-, 313.1274 [M-H-CH_3OH-CO_2]-, 227.1004 [M-H-Glc]-, 183.0686 [M-H-Glc-C_2H_4O]-, 161.0451, 151.0772 ($C_9H_{11}O_2$), 125.1006, 119.0356, 115.0415, 113.0246,101.0254, 83.0173	[2,8,26], [3,16]
					**5- Secoiridoids, glycosides and derivatives**				
					a. Oleoside and secologanoside derivatives				
**1**	Oleoside	$C_{16}H_{22}O_{11}$	390.11622	1.05	389.1084	389.1099	3.88	345.1213 [M-H-CO_2]-, 209.0475 [M-H-Glc-H_2O]-, 183.0677 [M-H-Glc-CO_2]-, 165.0569 [M-H-Glc-H_2O-CO_2]-, 139.0042 [M-H-glu-2CO_2]-, 121.0663 [M-H-Glc-H_2O-2CO_2]-, 119.0360, 113.0254, 89.0249	[1,6,8,29,31]
**2**	Secologanoside	$C_{16}H_{22}O_{11}$	390.11622	1.19	389.1084	389.1096	3.11	345.1255 [M-H-CO_2]-, 227.0579 [M-H-Glc]-, 209.0466 [M-H-Glc-H_2O]-, 183.0670 [M-H-Glc-CO_2]-, 165.0566 [M-H-Glc-H_2O-CO_2]-, 121.0657 [M-H-Glc-H_2O-2CO_2]-, 113.0277, 119.0359, 101.0219, 89.0238	[1-4,6,13,14]
**3**	Secoxyloganin	$C_{17}H_{24}O_{11}$	404.13187	1.76	403.1240	403.1252	2.76	371.1016 ($C_{16}H_{19}O_{10}$), 359.1345 [M-H-CO_2]-, 241.0711 [M-H-Glc]-, 223.0612 [M-H-Glc-H_2O]-, 197.0828 [M-H-Glc-CO_2]-, 179.0567 ($C_6H_{11}O_6$), 161.0460 (loss of water from 179.05), 119.0347, 113.0246, 101.0240, 89.0240	[1-4,6,16,22]

NO.	Identification	Molecular formula	Exact mass	RT (min)	[M-H]⁻ (Theo.) *m/z*	[M-H]⁻ (Exp.) *m/z*	Error * (ppm)	MS/MS fragments (*m/z*) negative mode	References of negative mode
**4**	Elenolic acid dighexoside (hexopyranosyl methyloleoside)	$C_{23}H_{34}O_{16}$	566.18469	4.16	565.1769	565.1768	-0.12	403.1241 [M-H-Glc]$^-$/($C_{17}H_{23}O_{11}$), 385.1215 [M-H-Glc-H_2O]$^-$/($C_{17}H_{21}O_{10}$), 241.0756 [M-H-2Glc]$^-$, 223.0619 [M-H-2Glc-H_2O]$^-$ ($C_{11}H_{11}O_5$), 179.0564 [M-H-2Glc-H_2O-CO_2]$^-$/($C_6H_{11}O_6$), 119.0349, 113.0242, 101.0249, 89.0248	[1,3,5,8,29,32]
**5**	Hexopyranosyl dimethyloleoside	$C_{40}H_{56}O_{26}$	952.30599	7.04	951.2982	951.2967	-1.54	879.2963, 807.2488, 789.2482 [M-H-Glc]$^-$, 547.1652 ($C_{17}H_{24}O_{11}$), 403.1243 [elenolic acid glucoside-H]$^-$, 371.0986 [elenolic acid glucoside-H-CH_4O]$^-$, 359.1312 [elenolic acid glucoside-H-CO_2]$^-$, 223.0608 [elenolic acid glucoside-H-Glc-H_2O]$^-$, 179.0576 ($C_6H_{11}O_6$)/[elenolic acid glucoside-H-Glc-H_2O-CO_2]$^-$	[24,33]
b. Oleuropein-type secoiridoid and derivatives									
**1**	Demethyl oleuropein	$C_{24}H_{30}O_{13}$	526.16865	3.92	525.1608	525.1619	2.05	481.1704 [M-H-CO_2]$^-$ ($C_{23}H_{29}O_{11}$), 389.1084 ($C_{16}H_{21}O_{11}$), 363.1087 [M-H-Glc]$^-$, 345.1072 [M-H-Glc-H_2O]$^-$, 319.1188 [demethyl oleuropein aglycone-H-CO_2]$^-$, 301.1092 [demethyl oleuropein aglycone-H-CO_2-H_2O]$^-$, 293.0680 [M-H-Glc-C_4H_6O]$^-$, 275.0934 [demethyl oleuropein aglycone-H-CO_2-C_2H_4O]$^-$, 249.0770 [demethyl oleuropein aglycone-H-CO_2-C_4H_6O]$^-$, 209.0455 [demethyl oleuropein aglycone-H-CO_2-C_4H_6O-C_3H_4]$^-$, 195.0663 [demethyl oleuropein aglycone-H-CO_2-H_2O-C_7H_6O]$^-$ ($C_{10}H_{11}O_4$), 183.0664, 165.0560 [demethyl oleuropein aglycone-H-CO_2 C_4H_6O-C_3H_4-CO_2]$^-$, 139.0039, 121.0655 [demethyl oleuropein aglycone-H-CO_2 C_4H_6O-C_3H_4-$2CO_2$]$^-$, 119.0357, 113.0244, 89.0246	[1,2,8,18,26,29,34]
**2**	Hydroxyoleuroside	$C_{26}H_{36}O_{13}$	556.2156	4.32	555.2078	555.2064	-2.47	537.2034 [M-H-H_2O]$^-$, 511.2170 [M-H-CO_2]$^-$ ($C_{25}H_{35}O_{11}$), 437.1059, 403.1378 ($C_{17}H_{23}O_{11}$) [M-H-H_2O-$C_8H_6O_2$]$^-$, 393.1579 [M-H-Glc]$^-$, 389.1108, 345.1201 ($C_{15}H_{21}O_9$), 225.1131, 183.1015, 165.0566, 151.0434, 149.0229, 139.0768, 123.0442, 121.0656	[8]
**3**	Hydroxy-*O*-decarboxymethyl oleuropein aglycone	$C_{17}H_{20}O_7$	336.12091	4.95	335.1131	335.1140	2.88	199.0621 [M-H-$C_8H_8O_2$]$^-$, 181.0506 [M-H-$C_8H_8O_2$-H_2O]$^-$, 155.0704 [M-H- $C_8H_8O_2$-CO_2]$^-$, 111.0820 [M-H-$C_8H_8O_2$-H-$2CO_2$]$^-$	[8], [28]
**4**	Methyl oleuropein aglycone	$C_{20}H_{24}O_8$	392.14712	5.41	391.1604	391.1599	-1.34	345.1582 [M-H-CH_2O_2]$^-$, 285.13 [M-H-CH_2O_2-$2CH_2O$]$^-$, 255.11 [M-H-CH_2O_2-$3CH_2O$]$^-$, 225.1139 [M-H-CH_2O_2-$4CH_2O$]$^-$, 183.1019 [M-H-CH_2O_2-$4CH_2O$-C_2H_2O]$^-$, 167.0717, 89.0202	[1,5]

NO.	Identification	Molecular formula	Exact mass	RT (min)	$[M-H]^-$ (Theo.) *m/z*	$[M-H]^-$ (Exp.) *m/z*	Error * (ppm)	MS/MS fragments (*m/z*) negative mode	References of negative mode
**5**	Oleuropein derivative 1 (dihydro-oleuropein)	$C_{26}H_{40}O_{12}$	544.25198	6.14	543.2442	543.2439	-0.48	497.2050 $[M-H-C_2H_5OH]^-$, 225.1145, 197.0828 ($C_{10}H_{13}O_4$), 153.0927 $[C_{10}H_{13}O_4-CO_2]^-$	[1,2,5,6,8,16]
**6**	Hydroxyoleuropein	$C_{25}H_{32}O_{14}$	556.17921	6.43	555.1714	555.1720	1.10	537.1613 $[M-H-H_2O]^-$ ($C_{25}H_{29}O_{13}$), 511.1179 $[M-H-CO_2]^-$, 403.1246 ($C_{17}H_{23}O_{11}$) $[M-H-H_2O-C_8H_6O_2]^-$, 393.1198 ($C_{19}H_{21}O_9$) $[M-H-Glc]^-$, 375.1115 $[M-H-Glc-H_2O]^-$, 361.0952 $[M-H-Glc-CH_3OH]^-$, 343.0869 $[M-H-Glc-CH_3OH-H_2O]^-$, 323.0791 $[M-H-C_4H_6O]^-$ ($C_{15}H_{15}O_8$) $[M-H-Glc-C_4H_6O]^-$, 291.0866 $[M-H-Glc-C_4H_6O-CH_3OH]^-$, 223.0623, 151.0404 (base peak), 149.0256, 123.0453	[1-4,8,14,16]
**7**	Dihydro-oleuropein	$C_{25}H_{36}O_{13}$	544.2156	7.06	543.2078	543.2078	0.06	525.1981 $[M-H-H_2O]^-$, 513.1967 $[M-H-CH_2O]^-$, 407.1531 $[M-H-CH_2O-C_7H_6O]^-$, 389.1464 $[M-H-CH_2O-C_7H_6O-H_2O]^-$, 377.1462 (frag. of oleuropein aglycone), 345.1235 [oleuropein aglycone-H-$CH_3OH]^-$, 313.1297, 197.0828 ($C_{10}H_{13}O_4$), 153.0932 (cleavage of hydroxytyrosol), 151.0773, 101.0256	[1,6,16]
**8**	Oleuropein hexocside (oleuricine A)	$C_{31}H_{42}O_{18}$	702.23712	7.12	701.2293	701.2313	2.85	539.1744 $[M-H-Glc]^-$ ($C_{25}H_{31}O_{13}$), 437.1403, 377.1222 [M-H-diglucoside]$^-$ ($C_{19}H_{21}O_8$), 345.0980 [M-H-diglucoside-$CH_3OH]^-$, 307.0813 ($C_{15}H_{15}O_7$), 275.0896 ($C_{15}H_{15}O_5$), 223.0620, 179.0560, 149.0243, 139.0396, 113.0230	[1,2,4-6,20]
**9**	Lucidumoside B (hydro-oleuropein)	$C_{25}H_{34}O_{13}$	542.19995	7.81	541.1921	541.1902	-3.55	405.1400 [hydrated elenolic acid glucoside-H]$^-$/[M-H-hydroxytyrosol-$H_2O]^-$, 361.1299 $[M-H-Glc-H_2O]^-$, 329.1042 $[M-H-Glc-H_2O-CH_3OH]^-$, 225.0781 [elenolic acid-H-$H_2O]^-$, 193.0516 [elenolic acid-H-H_2O-$CH_3OH]^-$, 181.0878 [elenolic acid-H-H_2O-$CO_2]^-$, 149.0622 [elenolic acid-H-H_2O-CO_2-$CH_3OH]^-$, 121.0666 [elenolic acid-H-H_2O-CO_2-CH_3OH-CO]$^-$	[1,29,35]
**10**	Oleuroside hexoside (oleuricine B)	$C_{31}H_{42}O_{18}$	702.23712	7.86	701.2293	701.2299	0.86	539.1731 $[M-H-Glc]^-$ ($C_{25}H_{31}O_{13}$), 377.1247 [M-H-diglucoside]$^-$ ($C_{19}H_{21}O_8$), 345.0987 [M-H-diglucoside-$CH_3OH]^-$, 307.0829 ($C_{15}H_{15}O_7$), 275.0923 ($C_{15}H_{15}O_5$), 223.0614, 179.0566, 149.0234, 139.0403, 113.0240	[1,2,4-6,20]
**11**	Oleuropeinic acid	$C_{25}H_{30}O_{15}$	570.15848	7.87	569.1507	569.1521	2.55	537.1666 $[M-H-CH_3OH]^-$, 407.1408 $[M-H-Glc]^-$, 403.1276 ($C_{17}H_{23}O_{11}$), 305.1045, 223.0635, 179.0566, 151.0417, 123.0454, 89.0253	[29]

NO.	Identification	Molecular formula	Exact mass	RT (min)	[M-H]$^-$ (Theo.) *m/z*	[M-H]$^-$ (Exp.) *m/z*	Error * (ppm)	MS/MS fragments (*m/z*) negative mode	References of negative mode
**12**	Methoxy-oleuropein	$C_{26}H_{34}O_{14}$	570.19486	7.94	569.1870	569.1862	-1.47	537.1603 [M-H-CH_3OH] / ($C_{25}H_{29}O_{13}$), 407.1343 ($C_{20}H_{23}O_9$) / [M-H-Glc]$^-$, 403.1241 [M-H-CH_3OH-$C_8H_6O_2$]$^-$ / ($C_{17}H_{23}O_{11}$), 375.1115 [M-H-Glc-CH_3OH]$^-$ ($C_{19}H_{19}O_8$), 357.0983 [M-H-Glc-CH_3OH-H_2O]$^-$, 337.0929 [M-H-Glc-C_4H_6O]$^-$, 305.1016 [M-H-Glc-C_4H_6O-CH_3OH]$^-$, 223.0620 ($C_{11}H_{11}O_5$), 165.0558, 151.0408, 149.0259, 139.0408, 127.0407, 113.0251, 111.0090, 95.0508, 89.0252	[1,2,8], [14]
**13**	Oleuropein	$C_{25}H_{32}O_{13}$	540.1843	8.07	539.1765	539.1779	2.68	507.1474 [M-H-CH_3OH]$^-$, 469.1309 [M-H-C_4H_6O]$^-$, 437.1299 [M-H-C_4H_6O-CH_2O]$^-$, 403.1206 [elenolic acid glucoside-H]$^-$/($C_{17}H_{23}O_{11}$), 377.1201 [M-H-Glc]$^-$ ($C_{19}H_{21}O_8$), 371.0970 [elenolic acid glucoside-H-CH_3OH]$^-$, 359.1166 [M-H-Glc-H_2O]$^-$, 345.0947 [M-H-Glc-CH_3OH]$^-$/($C_{18}H_{17}O_7$), 327.0858 [M-H-Glc-CH_3OH-H_2O]$^-$ ($C_{18}H_{15}O_6$), 307.0797 [M-H-Glc-C_4H_6O]$^-$ ($C_{15}H_{15}O_7$), 275.0856 [M-H-Glc-C_4H_6O-CH_3OH]$^-$ /($C_{15}H_{15}O_5$), 223.0595 [elenolic acid glucoside-H-Glc-H_2O]$^-$, 197.0812, 179.0568 [elenolic acid glucoside-H-Glc-H_2O-CO_2]$^-$, 165.0554, 153.0551 [hydroxytyrosol-H]$^-$, 149.0237 [elenolic acid glucoside-H-Glc-H_2O-CO_2-CH_3]$^-$, 139.0349, 123.0454 [hydroxytyrosol-H-CH_2O_2]$^-$/($C_7H_7O_2$), ($C_7H_7O_2$)/[hydroxytyrosol-H-CH_2O_2]$^-$, 119.0343, 101.0237, 89.0236	[4,11,30], [1-3,14,16,20,21]
**14**	Lucidumoside C	$C_{27}H_{36}O_{14}$	584.21051	8.77	583.2027	583.2042	2.53	537.1575 [M-H-OCH_3-CH_3]$^-$, 421.1475 [M-H-hexosyl residue]$^-$, 461.1567, 403.1212 [M-H-hexosyl residue-H_2O]$^-$, 375.1083 [M-H-hexosyl residue-CO]$^-$, 371.0977, 357.0976 [M-H-hexosyl residue-CO-H_2O]$^-$, 351.1074 [M-hexosyl residue-186.05]$^-$, 319.1129, 273.0749, 223.0600, 197.0829, 179.0571, 151.0397, 123.0452	[1-4,29,36]
**15**	Fraxamoside	$C_{25}H_{30}O_{13}$	538.16865	8.47	537.1608	537.1599	-1.71	403.1240 [M-H-$C_8H_6O_2$]$^-$ ($C_{17}H_{23}O_{11}$), 375.1114 [M-H-$C_8H_6O_2$-CO]$^-$ / ($C_{19}H_{19}O_8$), 273.0761, 223.0613 [M-H-$C_8H_6O_2$-Glc-H_2O]$^-$ / ($C_{11}H_{11}O_5$), 179.0572 [M-H-$C_8H_6O_2$-Glc-H_2O-CO_2]$^-$, 151.0400, 123.0456	[1,2,8]
**16**	Jaspolyoside	$C_{42}H_{54}O_{23}$	926.3056	9.09	925.2978	925.3011	3.58	893.2820 [M-H-CH_3OH]$^-$ / ($C_{41}H_{49}O_{22}$), 763.2521 [M-H-Glc]$^-$ / ($C_{36}H_{43}O_{18}$), 745.2246 [M-H-Glc-H_2O]$^-$ / ($C_{36}H_{41}O_{17}$), 693.1996 [M-H-Glc-C_4H_6O]$^-$ / ($C_{32}H_{37}O_{17}$), 539.1768 ($C_{25}H_{31}O_{13}$) (loss of elenolic acid glucoside moiety), 521.1670 [oleuropein-H-H_2O]$^-$ / ($C_{25}H_{29}O_{12}$), 403.1246 [elenolic acid glucoside-H]$^-$, 377.1238 [oleuropein-H-Glc]$^-$ ($C_{19}H_{21}O_8$), 345.0968 [oleuropein-H-Glc-CH_3OH]$^-$, 307.0829 [oleuropein-H-Glc-C_4H_6O]$^-$	[1,2,4,8,29], [37,38]

NO.	Identification	Molecular formula	Exact mass	RT (min)	$[M-H]^-$ (Theo.) *m/z*	$[M-H]^-$ (Exp.) *m/z*	Error * (ppm)	MS/MS fragments (*m/z*) negative mode	References of negative mode
								($C_{15}H_{15}O_7$), 275.0925 ($C_{15}H_{15}O_5$), 223.0624, 153.0560, 149.0246, 139.0422, 89.0255	
**17**	Hydroxy-oleuropein aglycone	$C_{19}H_{22}O_9$	394.12639	9.16	393.1186	393.1182	-0.92	361.0937 $[M-H-CH_3OH]^-$, 343.0860 $[M-H-CH_3OH-H_2O]^-$, 323.0661 [M-H-C_4H_6O]- / ($C_{15}H_{15}O_8$), 291.0876 $[M-H-Glc-C_4H_6O-CH_3OH]^-$, 223.0661, 151.0413, 149.0256, 123.0463, 113.0252, 111.0096, 101.0242, 95.0508	[1,8,26,39]
**18**	Oleuropein aglycone (DHPEA-EA) isomer 1	$C_{19}H_{22}O_8$	378.13147	9.92	377.1236	377.1236	-0.12	345.0934 $[M-H-CH_3OH]^-$ / ($C_{18}H_{17}O_7$), 327.0879 $[M-H-CH_3OH-H_2O]^-$, 307.0807 $[M-H-C_4H_6O]^-$ / ($C_{15}H_{15}O_7$), 275.0916 ($C_{15}H_{15}O_5$), 165.0536, 153.0619 (cleaved hydroxytyrosol), 149.0246, 139.0399, 123.0466 $[hydroxytyrosol-H-CH_2O]^-$	[1,14,21,30]
**19**	Demethyl oleuropein aglycone	$C_{18}H_{20}O_8$	364.11582	10.10	363.1080	363.1092	3.21	345.0919 $[M-H-H_2O]^-$, 331.0781 $[M-H-H_2O-CH_2]^-$, 313.0691 $[M-H-2H_2O-CH_2]^-$, 294.9074 $[M-H-3H_2O-CH_2]^-$, 250.9140 $[M-H-3H_2O-CH_2-CO_2]^-$, 183.0658 ($C_9H_{11}O_4$), 151.0407, 123.0461, 105.0343	[2,8,29,34,40]
**20**	Demethyl oleuropein	$C_{24}H_{30}O_{13}$	526.16865	3.92	525.1608	525.1619	2.05	481.1704 $[M-H-CO_2]^-$ / ($C_{23}H_{29}O_{11}$), 389.1084 ($C_{16}H_{21}O_{11}$), 363.1087 $[M-H-Glc]^-$, 345.1072 $[M-H-Glc-H_2O]^-$, 319.1188 [demethyl oleuropein aglycone-H-CO_2]$^-$, 301.1092 [demethyl oleuropein aglycone-H-CO_2-H_2O]$^-$, 293.0680 $[M-H-Glc-C_4H_6O]^-$, 275.0934 [demethyl oleuropein aglycone-H-CO_2-C_2H_4O]$^-$, 249.0770 [demethyl oleuropein aglycone-H-CO_2-C_4H_6O]$^-$, 209.0455 [demethyl oleuropein aglycone-H-CO_2-C_4H_6O-C_3H_4]$^-$, 195.0663 [demethyl oleuropein aglycone-H-CO_2-H_2O-C_7H_6O]$^-$ / ($C_{10}H_{11}O_4$), 183.0664, 165.0560 [demethyl oleuropein aglycone-H-CO_2 C_4H_6O-C_3H_4-CO_2]$^-$, 139.0039, 121.0655 [demethyl oleuropein aglycone-H-CO_2-C_4H_6O-C_3H_4-2CO_2]$^-$, 119.0357, 113.0244, 89.0246	[1,12,29,34,41]
**21**	Oleuropein Derivative 2	$C_{35}H_{46}O_{15}$	706.28368	10.39	705.2759	705.2759	0.13	603.2446 [oleuropein-H-2CH_3OH]$^-$, 539.1747 [oleuropein-H]$^-$ / ($C_{25}H_{31}O_{13}$), 521.1627 ($C_{25}H_{29}O$), 377.1249 [oleuropein-H-Glc]$^-$ / ($C_{19}H_{21}O_8$), 307.0810 [oleuropein-H-Glc-C_4H_6O]$^-$ / ($C_{15}H_{15}O_7$), 275.0934 ($C_{14}H_{11}O_6$), 223.0614, 149.0250	[4,8]
**22**	Oleuropein aglycone (DHPEA-EA) isomer 2	$C_{19}H_{22}O_8$	378.13147	11.57	377.1236	377.1226	-2.77	345.0978 $[M-H-CH_3OH]^-$, 327.0879 $[M-H-CH_3OH-H_2O]^-$, 307.0812 $[M-H-C_4H_6O]^-$, 275.0916 ($C_{15}H_{15}O_5$), 223.0608, 197.0829 (decarboxylation), 165.0559, 153.0551 (cleaved hydroxytyrosol),	[2,8,20,40], [16,22,26,37]

NO.	Identification	Molecular formula	Exact mass	RT (min)	$[M-H]^-$ (Theo.) *m/z*	$[M-H]^-$ (Exp.) *m/z*	Error * (ppm)	MS/MS fragments (*m/z*) negative mode	References of negative mode
								149.0237, 139.0348, 127.0397, 123.0449 [hydroxytyrosol-H-CH_2O]$^-$, 113.0243, 111.0083, 101.0241, 95.0495	
**23**	Dimethyl oleuropein aglycone	$C_{21}H_{26}O_8$	406.16277	14.59	405.1549	405.1557	1.87	373.1343 [M-H-CH_3OH]$^-$, 269.1001 [M-H-126.05]$^-$, 237.0771 [M-H-hydroxytyrosol-H_2O-CH_3OH]$^-$, 225.1134 [M-H-hydroxytyrosol-H_2O-CO_2]$^-$, 179.0704, 153.0615 (cleaved of hydroxytyrosol), 147.004, 123.0445 [hydroxytyrosol-H-CH_2O]$^-$, 119.0510	[1]
c. ligstroside-type secoiridoid derivatives									
**1**	Demethyl ligstroside	$C_{24}H_{30}O_{12}$	510.17373	4.55	509.1659	509.1681	4.31	465.1731 [M-H-CO_2]$^-$, 463.1792 [M-H-CH_2O_2]$^-$, 389.1076 [M-H-dehydrated tyrosol]$^-$, 347.1151 [M-H-Glc]$^-$, 329.0978 [M-H-Glc-H_2O]$^-$, 285.1146 [M-H-Glc-H_2O-CO_2]$^-$, 277.0739 [M-H-Glc-C_4H_6O]$^-$, 233.0805 [M-H-Glc-C_4H_6O-CO_2]$^-$, 165.0581 [M-H-glc-$CH_2CHPhOH$-H_2O-CO_2]$^-$, 121.0660 [M-H-glc-$CH_2CHPhOH$-H_2O-2CO_2]$^-$	[13,27,40]
**2**	Ligstrosidic acid	$C_{25}H_{30}O_{14}$	554.16356	7.69	553.1557	553.1537	-3.68	521.1240 [M-H-CH_3OH]$^-$, 477.1328, 451.1382, 433.0938 (loss of dehydrated tyrosol residue; C_8H_8O) to form $C_{17}H_{21}O_{13}$, 391.1017 [M-H-Glc]$^-$, 373.0920 [M-H-Glc-H_2O]$^-$, 313.0657 [M-H-Glc-H_2O-2CH_2O]$^-$, 289.0688 [M-H-Glc-H_2O-2C_2H_2O]$^-$, 209.0433 [M-H-tyrosol-Glc-CHO_2]$^-$, 167.0357 [M-H-tyrosol-Glc-CHO_2-C_2H_2O]$^-$, 151.0364	[42,43]
**3**	Ligstroside-3'-glucoside	$C_{31}H_{42}O_{17}$	686.24221	7.81	685.2344	685.2354	1.42	523.1781 [M-H-Glc]$^-$, 361.1295 [M-H-Glc-Glc]$^-$ /[ligstroside aglycone-H]$^-$, 291.0896 [M-H-2Glc-C_4H_6O]$^-$, 259.0983 [M-H-2Glc-C_4H_6O-CH_3OH]$^-$, 223.0613 [elenolic acid-H-H_2O]$^-$, 137.0615 (cleavage of tyrosol)	[26]
**4**	Ligstroside (4-HPE-EA-glucoside)	$C_{25}H_{32}O_{12}$	524.18938	8.98	523.1816	523.1806	-1.83	453.1376 [M-H-C_4H_6O]$^-$, 403.1284, 361.1287 [M-H-Glc]$^-$, 291.0876 [M-H-Glc-C_4H_6O]$^-$, 259.0980 [M-H-Glc-C_4H_6O-CH_3OH]$^-$, 223.0634 [elenolic acid-H-H_2O]$^-$ /($C_{11}H_{11}O_5$), 179.0627 [elenolic acid-H-H_2O-CO_2]$^-$, 137.0611 [tyrosol-H]$^-$, 127.0406, 119.0353, 111.0089, 101.0248, 89.0245	[2,3,5,14,21,40,41]
**5**	Jaspolyanoside	$C_{42}H_{54}O_{22}$	910.31068	9.80	909.3029	909.3030	0.20	747.2607 [M-H-Glc]$^-$/($C_{36}H_{43}O_{17}$), 677.2013 [M-H-Glc-C_4H_6O]$^-$/($C_{32}H_{37}O_{16}$), 523.178 [ligstroside-H]$^-$ /($C_{25}H_{31}O_{12}$), 361.133 [ligstroside-H-Glc]$^-$ /($C_{19}H_{21}O_7$), 291.090 [ligstroside-H-Glc-C_4H_6O]$^-$/($C_{15}H_{15}O_6$),	[1,8,26,37,38]

NO.	Identification	Molecular formula	Exact mass	RT (min)	[M-H]$^-$ (Theo.) *m/z*	[M-H]$^-$ (Exp.) *m/z*	Error * (ppm)	MS/MS fragments (*m/z*) negative mode	References of negative mode
								259.1082 [M-H-Glc-C_4H_6O-CH_3OH]$^-$, 223.0667 [elenolic acid-H-H_2O]$^-$ /($C_{11}H_{11}O_5$)	
**6**	Ligstroside aglycone (HPE-EA) / (HPEA-EA)	$C_{19}H_{22}O_7$	362.13656	11.40	361.1287	361.1290	0.75	329.1067, 291.0885 [M-H-C_4H_6O]$^-$, 259.0959 [M-H-C_4H_6O-CH_3OH]$^-$, 241.0813, 202.9141, 171.0275, 137.0566, 127.0415, 101.0238	[1,4,5,16,22,40]
d. Nuzhenide-type secoiridoids									
**1**	Neo-nüzhenide (hydroxylated of nuzhenide)	$C_{31}H_{42}O_{18}$	702.23712	6.9	701.2293	701.2290	-0.42	539.1769 [M-H-Glc]$^-$, 507.1507 [M-H-Glc-CH_3OH]$^-$, 469.1196 [M-H-Glc-C_4H_6O]$^-$, 437.1403, 377.1166, 315.1047 (fragmnet of hydroxytyrosol hexoside; $C_{14}H_{19}O_8$), 153.0559 [hydroxytyrosol-H]$^-$	[43]
**2**	Nüzhenide (nuzhenide)	$C_{31}H_{42}O_{17}$	686.24221	7.08	685.2344	685.2329	-2.16	523.1807 [M-H-Glc]$^-$, 453.1324 [M-H-Glc-C_4H_6O]$^-$, 421.1477 [hydroxyelenolic acid glucoside-H]$^-$ /($C_{17}H_{25}O_{12}$), 403.1242 [hydroxyelenolic acid glucoside-H-H_2O]$^-$/($C_{17}H_{23}O_{11}$), 299.1247 [M-H-dehydrated elenolic acid glucoside]$^-$, 223.0575 [elenolic acid glucoside-H-Glc-H_2O]$^-$, 179.0542 [elenolic acid glucoside-H-Glc-H_2O-CO_2]$^-$, 149.0237 [elenolic acid glucoside-H-Glc-H_2O-CO_2-2CH_3]$^-$	[1,4,14,18,26,44]
e. Other secoiridoids									
**1**	Acyclodihydro-elenolic acid hexoside	$C_{17}H_{28}O_{11}$	408.16317	0.99	407.1553	407.1572	4.57	389.1469 [M-H-H_2O]$^-$/($C_{17}H_{25}O_{10}$), 377.1473 [M-H-CH_2O]$^-$/($C_{16}H_{25}O_{10}$), 213.0779 [M-H-hexosyl moiety-CH_3OH]$^-$, 183.0652 [M-H-hexosyl moiety-CH_3OH-CH_2O]$^-$ ($C_{10}H_{15}O_3$), 164.9864, 151.0775 ($C_9H_{11}O_2$), 139.0795 [M-H-hexosyl moiety-CH_3OH-CH_2O-CO_2]$^-$, 126.9967, 119.0384, 115.0396	[1,2,5,35,45]
**2**	Nuzhenal A	$C_{10}H_{14}O_5$	214.08413	1.73	213.0763	213.0769	2.59	195.0618 [M-H-H_2O]$^-$, 183.0653 [M-H-CH_2O]$^-$, 165.0548 [M-H-CH_2O-H_2O]$^-$, 150.0312 [M-H-CH_2O-H_2O-CH_3]$^-$, 151.0765, 122.0385 [M-H-CH_2O-H_2O-CH_3-CO]$^-$, 121.0666, 107.0833 [M-H-CH_2O-H_2O-2CH_3-CO]$^-$, 84.9907	[43]
**3**	Ethyl-hydroxy-propionylcyclo-hexyl) acetic acid hexoside	$C_{19}H_{32}O_9$	404.20464	2.43	403.1968	403.1980	2.87	371.0842 [M-H-CH_3OH]$^-$, 340.9065 [M-H-CH_3OH-CH_2O]$^-$, 241.1414 [M-H-Glc]$^-$, 223.1328 [M-H-Glc-H_2O]$^-$	[1,28]
**6- Lignans, glycosides, and derivatives**									
**1**	Cycloolivil glucoside	$C_{26}H_{34}O_{12}$	538.20503	5.18	537.1972	537.1977	0.91	375.1444 [M-H-Glc]$^-$, 360.1283 [M-H-Glc-CH_3]$^-$, 345.1340 [M-H-Glc-CH_2O]$^-$, 327.1250 [M-H-Glc-CH_2O-H_2O]$^-$, 195.0664, 179.0714 ($C_{10}H_{11}O_3$), 164.0479, 146.0366	[2,26]

**NO.**	**Identification**	**Molecular formula**	**Exact mass**	**RT (min)**	**$[M-H]^-$ (Theo.) *m/z***	**$[M-H]^-$ (Exp.) *m/z***	**Error * (ppm)**	**MS/MS fragments (*m/z*) negative mode**	**References of negative mode**
**2**	Olivil glycoside	$C_{26}H_{34}O_{12}$	538.20503	5.33	537.1972	537.1973	0.18	375.1450 $[M-H-Glc]^-$, 360.12806 $[M-H-Glc-CH_3]^-$, 345.1310 $[M-H-Glc-CH_2O]^-$, 327.1216 $[M-H-Glc-CH_2O-H_2O]^-$, 195.0671, 179.0719, 164.0473, 146.0369	[2,26]
**3**	Eleutheroside E (syringaresinol diglucoside)	$C_{34}H_{46}O_{18}$	742.26842	5.77	741.2606	741.2606	0.01	579.2085 $[M-H-Glc]^-$/($C_{20}H_{21}O_7$), 417.1555 $[M-H-2Glc]^-$/[syringaresinol aglycone-H]$^-$, 181.0514 [syringaldehyde-H]$^-$/[syringaresinol aglycone-H-$C_{14}H_{17}O_4$]$^-$/($C_9H_9O_4$)	[46]
**4**	Hydroxypinoresinol-hexopyranoside isomer 1	$C_{26}H_{32}O_{12}$	536.18938	6.24	535.1816	535.1830	2.70	373.1283 $[M-H-Glc]^-$/($C_{20}H_{21}O_7$), 355.1169 [Hydroxypinoresinol-H-H_2O]$^-$/$[M-H-Glc-H_2O]^-$, 343.1190 $[M-H-Glc-CH_2O]^-$, 325.1108 $[M-H-Glc-CH_2O-H_2O]^-$, 313.1108 $[M-H-Glc-2CH_2O]^-$, 295.0997 $[M-H-Glc-2CH_2O-H_2O]^-$, 193.0485, 181.0518 $[M-H-Glc-CH_2O-C_{10}H_{10}O_2]^-$	[2,5,8,26,33]
**5**	Olivil/ Cycloolivil	$C_{20}H_{24}O_7$	376.15221	6.71	375.1444	375.1443	-0.21	360.1246 $[M-H-CH_3]^-$, 345.1353 $[M-H-CH_2O]^-$, 327.1226 $[M-H-CH_2O-H_2O]^-$, 312.1005 $[M-H-CH_2O-H_2O-CH_3]^-$, 297.1129 $[M-H-2CH_2O]^-$, 207.0675 ($C_{11}H_{11}O_4$), 195.0665 ($C_{10}H_{11}O_4$), 191.0715, 179.0718 ($C_{10}H_{11}O_3$), 164.0480, 146.0383, 122.0374	[2,8,26,37]
**6**	Pinoresinol hexoside	$C_{26}H_{32}O_{11}$	520.19447	7.14	519.1866	519.1863	-0.58	357.1344 $[M-H-Glc]^-$, 339.1254 $[M-H-Glc-H_2O]^-$, 327.1256 $[M-H-Glc-CH_2O]^-$, 309.1131 $[M-H-Glc-CH_2O-H_2O]^-$, 151.0409, 136.0173 (typical fragments of a 4-hydroxy-3-methoxy-benzophenone)	[33,47]
**7**	Hydroxypinoresinol -*O*-hexopyranoside isomer 2	$C_{26}H_{32}O_{12}$	536.18938	7.21	535.1816	535.1830	2.70	499.2156, 373.1295 $[M-H-Glc]^-$, 355.1179 $[M-H-Glc-H_2O]^-$, 343.1189 $[M-H-Glc-CH_2O]^-$, 325.1097 $[M-H-Glc-CH_2O-H_2O]^-$, 295.0979 $[M-H-Glc-2CH_2O-H_2O]^-$, 193.0496, 181.0513 $[M-H-Glc-CH_2O-C_{10}H_{10}O_2]^-$	[2,5,8,26]
**8**	Fraxiresinol-*O*-glucoside	$C_{27}H_{34}O_{13}$	566.19995	7.26	565.1921	565.1914	-1.27	403.1408 $[M-H-Glc]^-$, 385.1287 $[M-H-Glc-H_2O]^-$, 373.1292 $[M-H-Glc-CH_2O]^-$, 355.1190 $[M-H-Glc-CH_2O-H_2O]^-$,343.1196 $[M-H-Glc-2CH_2O]^-$, 325.1087 $[M-H-Glc-2CH_2O-H_2O]^-$, 310.0829 $[M-H-Glc-2CH_2O-H_2O-CH_3]^-$, 280.0846, 166.0265	[26,27,33]
**9**	Syringaresinol-*O*-glucoside	$C_{28}H_{36}O_{13}$	580.2156	7.40	579.2078	579.2050	-4.78	543.2078 $[M-H-2H_2O]^-$, 533.1650 $[M-H-C_2H_5OH]^-$, 525.1975 $[M-H-3H_2O]^-$, 417.1551 $[M-H-Glc]^-$, 402.1285 $[M-H-Glc-CH_3]^-$, 387.1094 $[M-H-Glc-2CH_3]^-$, 377.1402, 353.0964, 341.1017, 337.1092, 181.0496 [syringaldehyde-H]$^-$/($C_9H_9O_4$), 166.0283 [syringaldehyde-H-CH_3]$^-$	[27]
**10**	Acetoxy-pinoresinol-hexoside	$C_{28}H_{34}O_{13}$	578.19995	7.59	577.1921	577.1913	-1.42	415.1401 $[M-H-Glc]^-$ / ($C_{22}H_{23}O_8$), 373.1298 $[M-H-Glc-C_2H_2O]^-$, 325.1068 $[M-H-Glc-C_2H_2O-CH_2O-H_2O]^-$, 295.0993 $[M-H-Glc-C_2H_2O-2CH_2O-H_2O]^-$, 235.0618 $[M-H-Glc-CO_2-C_2H_5OH-3CH_2O]^-$, 151.0414	[8,26]

NO.	Identification	Molecular formula	Exact mass	RT (min)	$[M-H]^-$ (Theo.) *m/z*	$[M-H]^-$ (Exp.) *m/z*	Error * (ppm)	MS/MS fragments (*m/z*) negative mode	References of negative mode
**11**	Hydroxy-syringaresinol	$C_{22}H_{26}O_9$	434.15769	8.032	433.1499	433.1510	2.56	418.1307 $[M-H-CH_3]^-$, 403.1474 $[M-H-2CH_3]^-$, 385.1253 $[M-H-2CH_3-H_2O]^-$, 373.1327 $[M-H-2CH_2O]^-$, 358.1039 $[M-H-2CH_2O-CH_3]^-$, 343.0890 $[M-H-2CH_2O-2CH_3]^-$, 181.0500 [syringaldehyde-H]$^-$ / ($C_9H_9O_4$), 166.0292 [syringaldehyde-H-CH_3]$^-$, 138.0380 [syringaldehyde-H-CH_3-CO]$^-$	[48]
**12**	Hydroxy-pinoresinol	$C_{20}H_{22}O_7$	374.13656	8.17	373.1287	373.1297	2.68	355.1269 $[M-H-H_2O]^-$, 343.1220 $[M-H-CH_2O]^-$, 328.0940 $[M-H-CH_2O-CH_3]^-$, 313.1132 $[M-H-2CH_2O]^-$, 298.0863 $[M-H-2CH_2O-CH_3]^-$, 284.1079 $[M-H-CHO]^-$, 269.0829 $[M-H-CHO-CH_3]^-$, 193.0505, 181.0524	[1,16,26]
**13**	Pinoresinol	$C_{20}H_{22}O_6$	358.14164	8.67	357.1338	357.1347	2.58	342.1104 $[M-H-CH_3]^-$, 339.1226 $[M-H-H_2O]^-$, 327.1243 $[M-H-CH_2O]^-$, 324.0976 $[M-H-H_2O-CH_3]^-$, 312.0984 $[M-H-CH_2O-CH_3]^-$, 309.0723 $[M-H-H_2O-2CH_3]^-$, 297.0792 $[M-H-CH_2O-2CH_3]^-$, 281.0857 $[M-H-H_2O-2CH_3-CO]^-$, 228.9319, 188.9401, 185.0787, 175.0446	[1,16,29,32,49,50]
**14**	Syringaresinol	$C_{22}H_{26}O_8$	418.16277	9.6043	417.1549	417.1554	1.09	402.1362 $[M-H-CH_3]^-$, 387.1084 $[M-H-2CH_3]^-$, 371.1572 $[M-H-H_2O-CO]^-$, 181.0501 [syringaldehyde-H]$^-$, 166.0248 [syringaldehyde-H-CH_3]$^-$	[1,51,52]
**15**	Buddlenol D	$C_{33}H_{40}O_{13}$	644.2469	9.9661	643.2391	643.2404	2.14	595.2184 $[M-H-CH_2O-H_2O]^-$, 417.1544 [syringaresinol-H]$^-$, 387.1413 [syringaresinol-H-CH_2O]$^-$, 225.0780 $[M-H-C_{22}H_{26}O_8]^-$/($C_{11}H_{13}O_5$), 195.0675 $[M-H-C_{22}H_{26}O_8-CH_2O]^-$, 180.0421 [syringaresinol-H-CH_3]$^-$, 165.0294 [syringaresinol-H-2CH_3]$^-$, 151.0488 [syringaresinol-H-CH_3-CHO]$^-$, 137.0200 [syringaresinol-H-2CH_3-CO]$^-$	[48]
**16**	1-Acetoxy-pinoresinol	$C_{22}H_{24}O_8$	416.14712	10.21	415.1393	415.1400	1.73	400.1208 $[M-H-CH_3]^-$, 377.1239, 373.1263 $[M-H-C_2H_2O]^-$ / [Hydroxypinoresinol-H]$^-$, 356.1254 $[M-H-COOCH_3]^-$, 325.1082 $[M-H-C_2H_2O-CH_2O-H_2O]^-$, 295.0977 $[M-H-C_2H_2O-2CH_2O-H_2O]^-$, 280.0744 $[M-H-C_2H_2O-2CH_2O-H_2O-CH_3]^-$, 265.0495 $[M-H-C_2H_2O-2CH_2O-H_2O-2CH_3]^-$, 253.0722, 235.0609, 207.0662, 205.0500, 193.0502, 181.0508, 177.0146, 161.0613, 151.0406, 136.0167, 123.0467	[1,16,26]
					**7- Hydroxycoumarins and derivatives**				

NO.	Identification	Molecular formula	Exact mass	RT (min)	$[M-H]^-$ (Theo.) *m/z*	$[M-H]^-$ (Exp.) *m/z*	Error * (ppm)	MS/MS fragments (*m/z*) negative mode	References of negative mode
1	Esculin/aesculin (esculetin glucoside)	$C_{15}H_{16}O_9$	340.07944	2.35	339.0716	339.0727	3.10	177.0197 $[M-H-Glc]^-$, 149.0252 $[M-H-Glc-CO]^-$, 133.0305 $[M-H-Glc-CO_2]^-$ / ($C_8H_5O_2$), 105.0351 $[M-H-Glc-CO-CO_2]^-$, 89.0397 $[M-H-Glc-2CO_2]^-$, 77.0407 $[M-H-Glc-2CO-CO_2]^-$	[2,14,26,30]
2	Esculetin/ aesculetin (dihydroxy-coumarin)	$C_9H_6O_4$	178.02661	6.22	177.0188	177.0186	-1.05	149.0239 $[M-H-CO]^-$, 133.0288 $[M-H-CO_2]^-$, 105.0338 $[M-H-CO-CO_2]^-$, 89.0394 $[M-H-2CO_2]^-$, 77.0388 $[M-H-2CO-CO_2]^-$, 67.0191	[2,14,26]
3	Scopoletin (6-methyl esculetin)	$C_{10}H_8O_4$	192.04226	6.54	191.0344	191.0349	2.43	176.0118 $[M-H-CH_3]^-$, 148.0155 $[M-H-CH_3-CO]^-$, 120.0206 $[M-H-CH_3-2CO]^-$, 104.0265 $[M-H-CH_3-CO-CO_2]^-$	[2,14,26]
4	Umbelliferone (hydroxycoumarin)	$C_9H_6O_3$	162.0317	6.61	161.0239	161.0245	3.91	133.0288 $[M-H-CO]^-$, 123.0377, 117.0348 $[M-H-CO_2]^-$, 105.0344 $[M-H-2CO]^-$, 89.0401 $[M-H-CO_2-CO]^-$, 77.0397 $[M-H-3CO]^-$	[53]
**8- Flavonoid derivatives**									
a. Flavanones									
1	Naringenin hexoside	$C_{21}H_{22}O_{10}$	434.1213	7.51	433.1135	433.1140	1.21	271.0610 $[M-H-hexosyl]^-$, 151.0035 $[M-H-hexosyl-C_8H_8O]^-$, 119.0494 $[M-H-hexosyl-C_7H_4O_4]^-$, 107.0113 $[M-H-hexosyl-C_8H_8O-CO_2]^-$, 93.0359 $[M-H-hexosyl-C_9H_6O_4]^-$, 83.0130	[2,14]
2	Naringenin	$C_{15}H_{12}O_5$	272.06848	9.56	271.0607	271.0616	3.45	253.0535 $[M-H-H_2O]^-$, 243.0676 $[M-H-CO]^-$, 229.0517 $[M-H-C_2H_2O]^-$, 227.0723 $[M-H-CO_2]^-$, 177.0200 $[M-H-C_6H_6O]^-$, 151.0036 $[M-H-C_8H_8O]^-$, 119.0481 $[M-H-C_7H_4O_4]^-$, 107.0141 $[M-H-C_8H_8O-CO_2]^-$, 93.0354 $[M-H-C_9H_6O_4]^-$, 83.0143	[1,2,14,54]
3	Dihydroxy flavanone (pinocembrin)	$C_{15}H_{12}O_4$	256.07356	13.13	255.0657	255.0666	3.39	227.0689 $[M-H-CO]^-$, 213.0563 $[M-H-C_2H_2O]^-$, 211.0797 $[M-H-CO_2]^-$, 171.0457 $[M-H-2C_2H_2O]$-, 151.0053, 145.0650 $[M-H-C_2H_2O-C_3O_2]^-$, 107.0143 $[M-H-C_2H_2O-C_7H_6O]^-$/$[M-H-C_8H_8O-CO_2]^-$, 83.0143	[7,49]
b. Flavanonols									
1	Taxifolin-3-hexoside	$C_{21}H_{22}O_{12}$	466.11113	4.22	465.1033	465.1055	4.72	303.0520 $[M-H-Glc]^-$, 285.0382 ($C_{15}H_9O_6$) $[M-H-Glc-H_2O]^-$, 275.0547 $[M-H-Glc-CO]^-$, 259.0643 $[M-H-Glc-CO_2]^-$, 217.0538 $[M-H-Glc-CO_2-C_2H_2O]^-$, 125.0264 ($C_6H_5O_3$)	[30]
2	Taxifolin (dihydroquercetin)	$C_{15}H_{12}O_7$	304.05831	6.03	303.0505	303.0515	3.31	285.0422 $[M-H-H_2O]^-$, 275.0579 $[M-H-CO]^-$, 259.0666 $[M-H-CO_2]^-$, 217.0514 $[M-H-CO_2-C_2H_2O]^-$, 125.0251 ($C_6H_5O_3$), 83.0148	[1,2,8,14,19,30,55]

NO.	Identification	Molecular formula	Exact mass	RT (min)	[M-H]$^-$ (Theo.) *m/z*	[M-H]$^-$ (Exp.) *m/z*	Error * (ppm)	MS/MS fragments (*m/z*) negative mode	References of negative mode
**3**	Dihydrokaempfer-ol	$C_{15}H_{12}O_6$	288.06339	6.79	287.0556	287.0559	1.25	269.0465 [M-H-H_2O]$^-$, 259.0614 [M-H-CO]$^-$, 243.0670 [M-H-CO_2]$^-$, 215.0724 [M-H-2CO_2]$^-$, 201.0565 [M-H-CO_2-C_2H_2O]$^-$, 177.0565 [M-H-2CO_2-C_3H_2]$^-$, 151.0040, 125.0243 [M-H-CO-$C_8H_6O_2$]$^-$, 83.0144	[56,57]
				c. Flavones					
**1**	Luteolin-7-*O*-hexoside	$C_{21}H_{20}O_{11}$	448.10056	6.75	447.0927	447.0926	-0.31	285.0411 [M-H-Glc]$^-$, 255.0294 [M-H-Glc-CH_2O]$^-$, 227.0360 [M-H-Glc-CH_2O-CO]$^-$	[1-3,13,14,22,32]
**2**	Apigenin -*O*-rutinoside (Apigenin-*O*-hexosyl rhamnoside / Isorhoifolin)	$C_{27}H_{30}O_{14}$	578.16356	7.02	577.1557	577.1571	2.37	415.0879 [M-H-Rham]$^-$, 269.0469 [aglycone-H]$^-$/[M-H-glucorhamnoside]$^-$	[5,30] [1,2,21]
**3**	Apigenin -*O*-glucoside	$C_{21}H_{20}O_{10}$	432.10565	7.48	431.0978	431.0970	-1.91	311.0630, 269.0449 [M-H-Glc]$^-$, 268.0369, 225.0459 [M-H-Glc-CO_2]$^-$/($C_{14}H_9O_3$), 201.0513 ($C_{12}H_9O_3$), 151.0018	[2,3,5,8,11,13,16,30,32]
**4**	Chrysoeriol-*O*-glucoside (Methyl luteolin glucoside)	$C_{22}H_{22}O_{11}$	462.11622	7.71	461.1084	461.1101	3.66	446.0807 [M-H-CH_3]$^-$, 299.0567 [M-H-Glc]$^-$, 298.0494	[1,2,20,26]
**5**	Apigenin	$C_{15}H_{10}O_5$	270.05283	10.18	269.0450	269.0458	2.97	241.0512 [M-H-CO]$^-$, 225.0564 [M-H-CO2]$^-$ / ($C_{14}H_9O_3$), 201.0595 ($C_{12}H_9O_3$), 181.0647, 151.0044, 149.0236 ($C_8H_5O_3$), 121.0307, 117.0346, 107.0173,83.0128, 65.0022	[1,8,11-13,18,32]
**6**	Luteolin	$C_{15}H_{10}O_6$	286.04774	10.94	285.0399	285.0399	-0.05	257.0466 [M-H-CO]$^-$, 255.0253 [M-H-CH_2O]$^-$, 243.0298 [M-H-CO-CH_2]$^-$/($C_{13}H_7O_5$), 241.0522 ($C_{14}H_9O_4$), 229.0495 [M-H-2CO]$^-$, 199.0381 ($C_{12}H_7O_3$), 175.0413 ($C_{10}H_7O_3$), 151.0037, 133.0284, 107.0132	[1,3,11,13,14,29,32]
				d. Flavonols					
**1**	Rutin (quercetin-*O*-rutinoside)	$C_{27}H_{30}O_{16}$	610.15339	5.97	609.1456	609.1457	0.22	301.0344 ($C_{15}H_9O_7$) [M-H-Rham-Glc]$^-$, 300.0271	[1-3,8,15,16,32,58]
**2**	Quercetin glucoside (isoquercetrin)	$C_{21}H_{20}O_{12}$	464.09548	6.29	463.0877	463.0865	-2.49	301.0364 [M-H-Glc]$^-$, 300.0278 ($C_{15}H_8O_7$), 271.0245 [300.02-CHO]$^-$, 255.0318 [300.02-COOH]$^-$, 243.0393 [300.02-CHO-CO]$^-$	[1,2,8,30,32]
**3**	kaempferol-*O*-hexoside	$C_{21}H_{20}O_{11}$	448.10056	6.87	447.0927	447.0928	0.13	285.0407 [M-H-Glc]$^-$, 255.0316 [M-H-Glc-CH_2O]$^-$, 227.0356	[8]
**4**	Quercetin	$C_{15}H_{10}O_7$	302.04265	12.61	301.0348	301.0342	-2.09	273.0400 [M-H-CO]$^-$, 179.0001 [M-H-$C_7H_6O_2$]$^-$ /($C_8H_3O_5$), 151.0040 [M-H-$C_7H_6O_2$-CO]$^-$/($C_7H_3O_4$)	[1,8,14,21,30,32]

NO.	Identification	Molecular formula	Exact mass	RT (min)	$[M-H]^-$ (Theo.) *m/z*	$[M-H]^-$ (Exp.) *m/z*	Error * (ppm)	MS/MS fragments (*m/z*) negative mode	References of negative mode
**9- Terpenes (triterpenes)**									
**1**	Dihydroxy-oxo-oleanenoic acid	$C_{30}H_{46}O_5$	486.33453	10.89	485.3267	485.3288	4.33	439.3216 $[M-H-H_2O-CO]^-$	[29]
**2**	Asiatic acid	$C_{30}H_{48}O_5$	488.35018	12.54	487.3424	487.3419	-0.92	469.3287 $[M-H-H_2O]^-$	[43,59,60]
**3**	Hydroxy-oxo-oleanenoic acid	$C_{30}H_{46}O_4$	470.33961	14.73	469.3318	469.3314	-0.82	423.3253, 409.3338, 384.8595	[29]
**4**	2-α-Hydroxyursolic acid (corosolic acid)	$C_{30}H_{48}O_4$	472.35526	15.48	471.3474	471.3482	1.62	423.3291, 393.3070, 307.2444, 266.8802	[30,43,60]
**5**	Maslinic acid	$C_{30}H_{48}O_4$	472.35526	17.02	471.3474	471.3488	2.99	453.3334 $[M-H-H_2O]^-$, 407.3367 $[M-H-HCOOH]^-$	[30,49], [1,5,8,13,61]
**6**	Maslinic acid methyl ester	$C_{31}H_{50}O_4$	486.37091	19.15	485.3631	485.3643	2.45	453.3372 $[M-H-CH_3OH]^-$, 441.3395 $[M-H-C_2H_4O]^-$, 407.3418 $[M-H-CH_3OH-CH_2O_2]^-$	[5]
**7**	Oleanolic acid / Ursolic acid	$C_{30}H_{48}O_3$	456.36035	21.83	455.3525	455.3536	2.37	455.3536, 407.3302	[26,30,61,62]
**8**	Oleanolic acid / Ursolic acid	$C_{30}H_{48}O_3$	456.36035	22.21	455.3525	455.3505	-4.44	407.3302	[26,30,61,62]
**10- Fatty acids**									
a. Saturated fatty acid (SFA)									
**1**	Palmitic acid (Hexadecanoic acid)	$C_{16}H_{32}O_2$	256.24023	22.99	255.2324	255.2335	4.13	256.2386, 237.2300 $[M-H-H_2O]^-$	[13,63-70]
b. Monounsaturated fatty acids (MUSFA)									
**1**	2,3-dinor-8-iso-Prostaglandin F1 alpha	$C_{18}H_{32}O_5$	328.22498	7.00	327.2172	327.2162	-2.90	291.1964 $[M-H-2H_2O]^-$, 229.1433, 211.1330 (loss of H_2O from *m/z* 229.14), 171.1027	[28]
**2**	9,10,18Trihydroxyocta decenoic acid (9,10,18-TriHOME)	$C_{18}H_{34}O_5$	330.24063	7.60	329.2328	329.2331	0.91	311.2227 $[M-H-H_2O]^-$, 293.2126 $[M-H-2H_2O]^-$, 229.1452, 211.1343, 183.1395, 171.1032	[71]
**3**	Oleic Acid (*cis*-9-octadecenoic acid)	$C_{18}H_{34}O_2$	282.25588	23.53	281.2481	281.2481	0.16	282.2520, 280.9967, 280.34984	[1,65,66,68,70]
c. Polyunsaturated fatty acids (PUSFA)									

NO.	Identification	Molecular formula	Exact mass	RT (min)	$[M-H]^-$ (Theo.) *m/z*	$[M-H]^-$ (Exp.) *m/z*	Error * (ppm)	MS/MS fragments (*m/z*) negative mode	References of negative mode
**1**	Hydroxy-octadecatrienoic acid (hydroxylinolenic acid)	$C_{18}H_{30}O_3$	294.2195	11.81	293.2117	293.2122	1.81	275.2002 $[M-H-H_2O]^-$, 235.1697, 171.1039	[27]
**2**	Hydroxy-octadecadienoic acid	$C_{18}H_{32}O_3$	296.23515	13.48	295.2273	295.2278	1.63	277.2175 $[M-H-H_2O]^-$, 195.1388, 171.1034	[27]
**11- Other compounds**									
**1**	Vanillin glucoside (glucovanillin / vanilloside)	$C_{14}H_{18}O_8$	314.10017	2.67	313.0923	313.0933	3.03	151.0401 $[M-H-Glc]^-$, 123.0445 $[M-H-Glc-CO]^-$	[27]
**2**	Vanillin (hydroxymethoxy benzaldehyde)	$C_8H_8O_3$	152.04735	4.72	151.0395	151.0399	2.63	123.0453 $[M-H-CO]^-$, 122.0371 $[M-H-CHO]^-$, 121.0305 $[M-H-CH_2O]^-$, 108.0206 $[M-H-COCH_3]^-$, 105.0321 $[M-H-CH_2O_2]^-$	[2,3,14,32]
**3**	Syringaldehyde (hydroxybenzald-ehyde)	$C_9H_{10}O_4$	182.05791	6.22	181.0501	181.0505	2.29	166.0288 $[M-H-CH_3]^-$, 151.0037 $[M-H-2CH_3]^-$, 137.0254 $[M-H-CH_3-CHO]^-$, 123.0096 $[M-H-2CH_3-CO]^-$	[27,72,73]
**4**	Sinapaldehyde	$C_{11}H_{12}O_4$	208.07356	7.98	207.0657	207.0653	-2.10	192.0413 $[M-H-CH_3]^-$, 177.0185 $[M-H-2CH_3]^-$, 149.0241 $[M-H-2CH_3-CO]^-$, 121.0281 $[M-H-2CH_3-2CO]^-$	[27]
**5**	2-Phenylethyl -primeveroside -glucopyranoside, 2-phenylethyl 6-*O*--xylopyranosyl)	$C_{19}H_{28}O_{10}$	416.16825	5.92	415.1604	415.1608	0.82	311.0974 $[sugar-H]^-$, 251.0755 $[M-H-C_{10}H_{12}O_2]^-$ /($C_9H_{15}O_8$), 221.0654 $[M-H-C_{10}H_{12}O_2-CH_2O]^-$, 191.0587, 149.0454 ($C_5H_9O_5$)/$[M-H-C_{14}H_{18}O_5]^-$, 89.0249 $[M-H-C_{14}H_{18}O_5-2CH_2O]^-$	[1,6,28]
**6**	Malic acid (dicarboxylic acid)	$C_4H_6O_5$	134.02153	1.05	133.0137	133.0141	2.94	115.0036 $[M-H-H_2O]^-$, 89.0244 $[M-H-CO_2]^-$, 87.0085 $[M-H-CH_2O_2]^-$, 71.0140 $[M-H-CO_2-H_2O]^-$	[1,2,8,29]
**7**	Quinic acid (cyclohexane carboxylic acid)	$C_7H_{12}O_6$	192.06339	1.08	191.0556	191.0557	0.71	173.0470 $[M-H-H_2O]^-$, 127.0410 $[M-H-H_2O-CH_2O_2]^-$, 111.0092, 93.0344 $[M-H-H_2O-CH_2O_2-2OH]^-$, 87.0085, 85.0297, 67.0189	[1,2,29,74]

* Error (difference between the experimental mass and the theoretical mass of the compound (ppm)); Rt (retention time); Theo. (theoretical); Exp (experimental); FA (formic acid); Glc (glucosyl); Hex (hexosyl), Rham (rhamnosyl); Pent (pentosyl).

Supplementary Figures

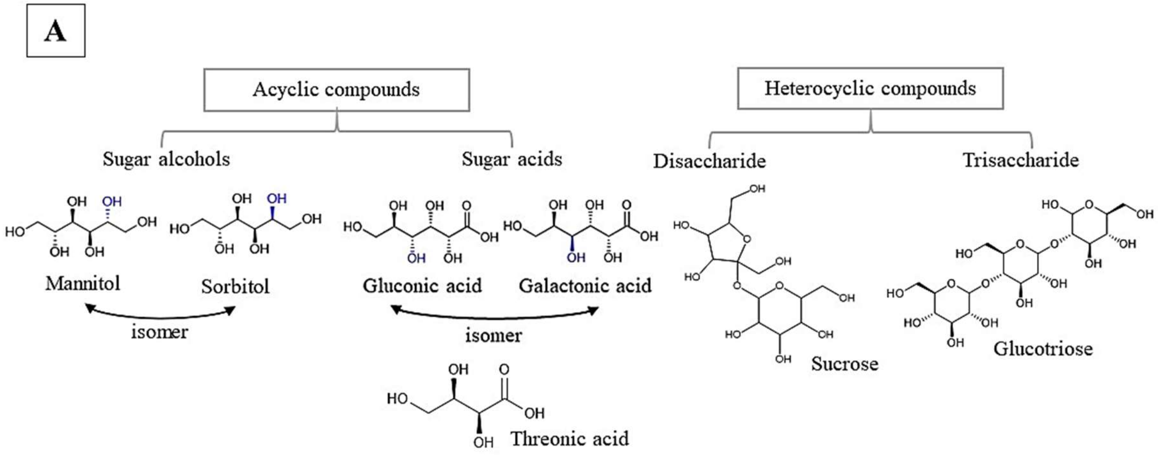

B

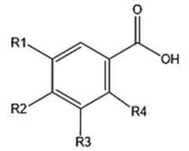

Compound name	R1	R2	R3	R4
**ρ-Hydroxybenzoic acid**	H	OH	H	H
**Salicylic acid**	H	H	H	OH
**3,4-Dihydroxybenzoic acid hexoside pentoside**	H	*O*-Hex.	*O*-Pent.	H
**Gallic acid**	OH	OH	OH	H
**Protocatechuic acid**	H	*O*-Glc.	OH	H
**Protocatechuic acid glucoside (DHBA glucoside)**	H	*O*-Glc.	OH	H
**Vanillic acid**	OCH_3	OH	H	H
**Vanillic acid glucoside**	OCH_3	*O*- Glc.	H	H

Figure S1. Chemical structures of tentatively identified compounds from olive stem extract; sugars and derivatives (A), phenolic acids (B).

A

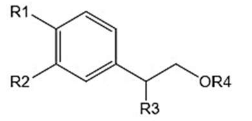

Compound name	R1	R2	R3	R4
**Tyrosol glucoside (salidroside)**	OH	H	H	Glc.
**Hydroxytyrosol (HTyr) (3,4-DHPEA)**	OH	OH	H	H
**Hydroxytyrosol glucoside (3,4-DHPEA glucoside)**	OH	OH	H	Glc.
**Hydroxytyrosol diglucoside**	*O*-Glc.	OH	H	Glc.
**3,4-Dihydroxyphenyl glycol**	OH	OH	OH	H

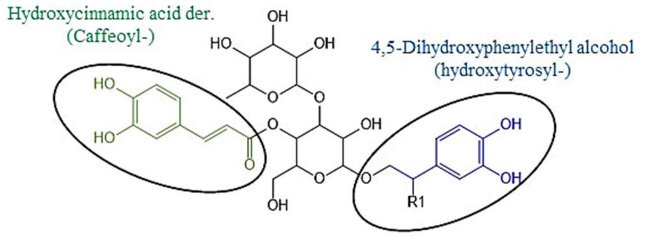

Compound name	R1
**Verbascoside (acteoside)**	H
***β*-Hydroxyverbascoside (*β*-hydroxyacteoside)**	OH

B

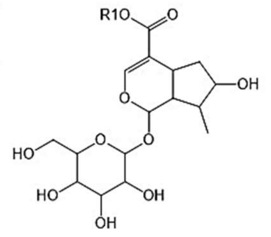

Compound name	R1
**Loganin (loganoside)**	CH_3
**Loganic acid**	H

Figure S2. Chemical structures of tentatively identified compounds from Olive stem extract; phenylethanoid and derivatives (A), and iridoid glycosides (B).

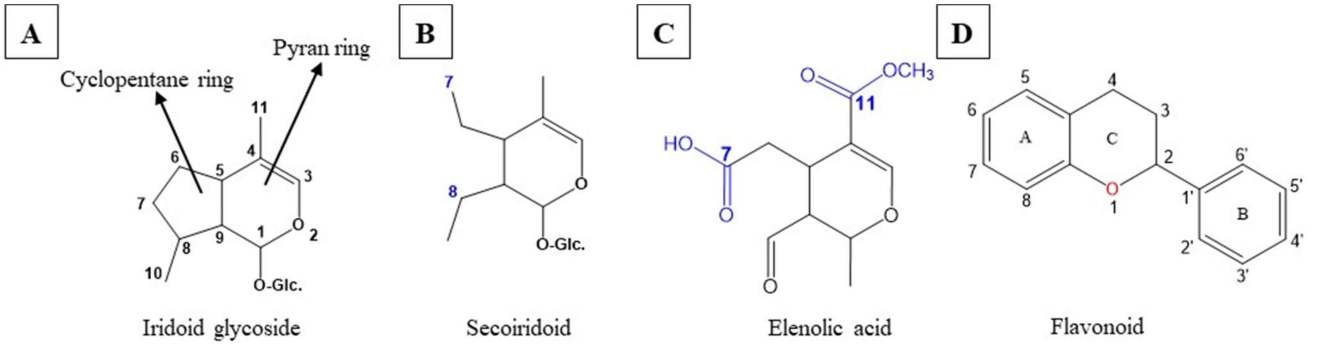

Figure S3. The general structure shows the numbering of certain positions of different classes detected in *Olea europaea*: iridoid glycoside (A), secoiridoid (B), elenolic acid (C), and flavonoid (D).

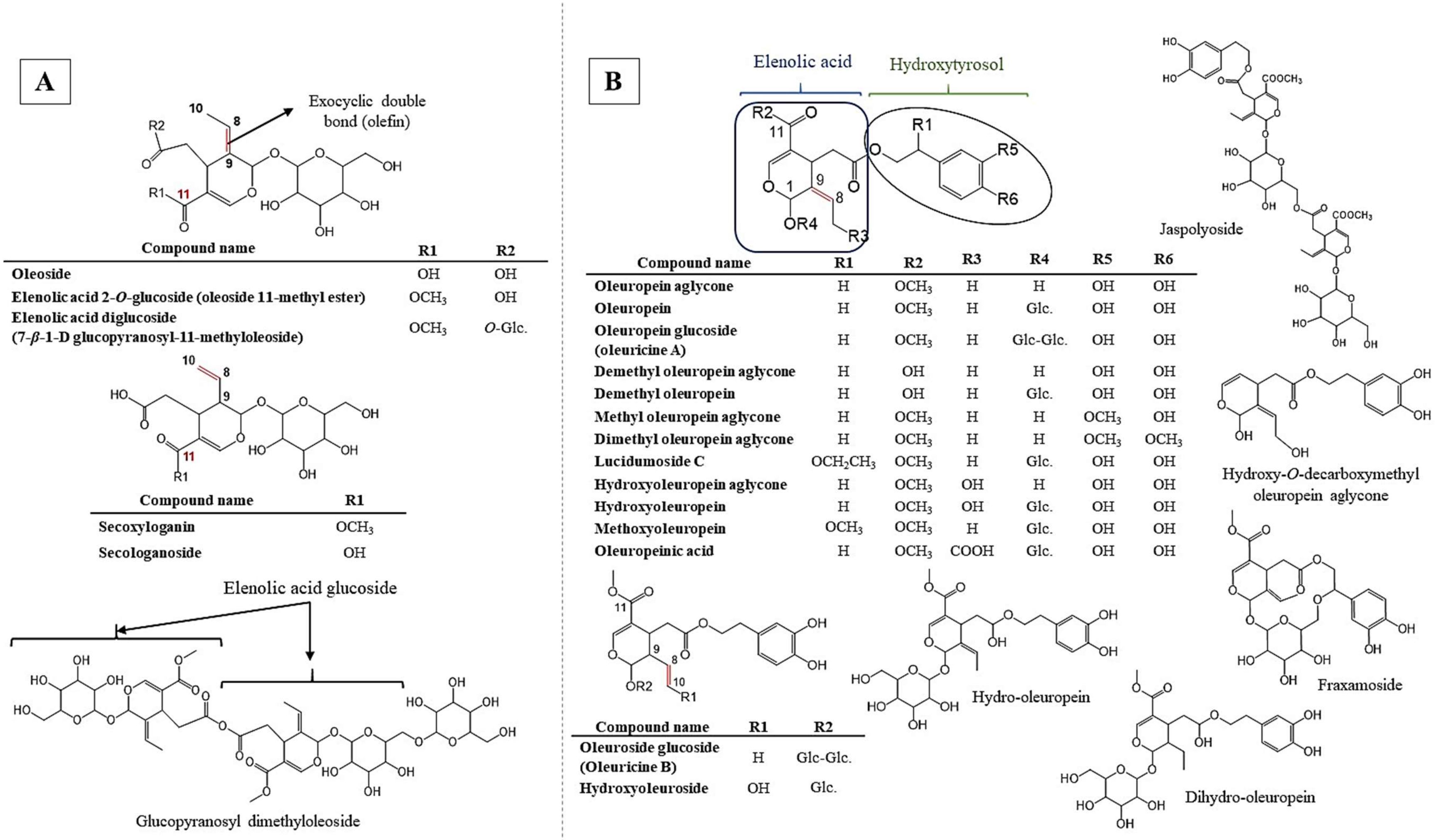

Compound name	R1	R2
**Oleoside**	OH	OH
**Elenolic acid 2-*O*-glucoside (oleoside 11-methyl ester)**	OCH_3	OH
**Elenolic acid diglucoside (7-*β*-1-D glucopyranosyl-11-methyloleoside)**	OCH_3	*O*-Glc.

Compound name	R1
**Secoxyloganin**	OCH_3
**Secologanoside**	OH

Compound name	R1	R2	R3	R4	R5	R6
**Oleuropein aglycone**	H	OCH_3	H	H	OH	OH
**Oleuropein**	H	OCH_3	H	Glc.	OH	OH
**Oleuropein glucoside (oleuricine A)**	H	OCH_3	H	Glc-Glc.	OH	OH
**Demethyl oleuropein aglycone**	H	OH	H	H	OH	OH
**Demethyl oleuropein**	H	OH	H	Glc.	OH	OH
**Methyl oleuropein aglycone**	H	OCH_3	H	H	OCH_3	OH
**Dimethyl oleuropein aglycone**	H	OCH_3	H	H	OCH_3	OCH_3
**Lucidumoside C**	OCH_2CH_3	OCH_3	H	Glc.	OH	OH
**Hydroxyoleuropein aglycone**	H	OCH_3	OH	H	OH	OH
**Hydroxyoleuropein**	H	OCH_3	OH	Glc.	OH	OH
**Methoxyoleuropein**	OCH_3	OCH_3	H	Glc.	OH	OH
**Oleuropeinic acid**	H	OCH_3	COOH	Glc.	OH	OH

Compound name	R1	R2
**Oleuroside glucoside (Oleuricine B)**	H	Glc-Glc.
**Hydroxyoleuroside**	OH	Glc.

Figure S4. Chemical structures of tentatively identified compounds from OSE belonging to secoiridoid compounds; oleoside and secologanoside (A), oleuropein-type secoiridoids (B), and their derivatives.

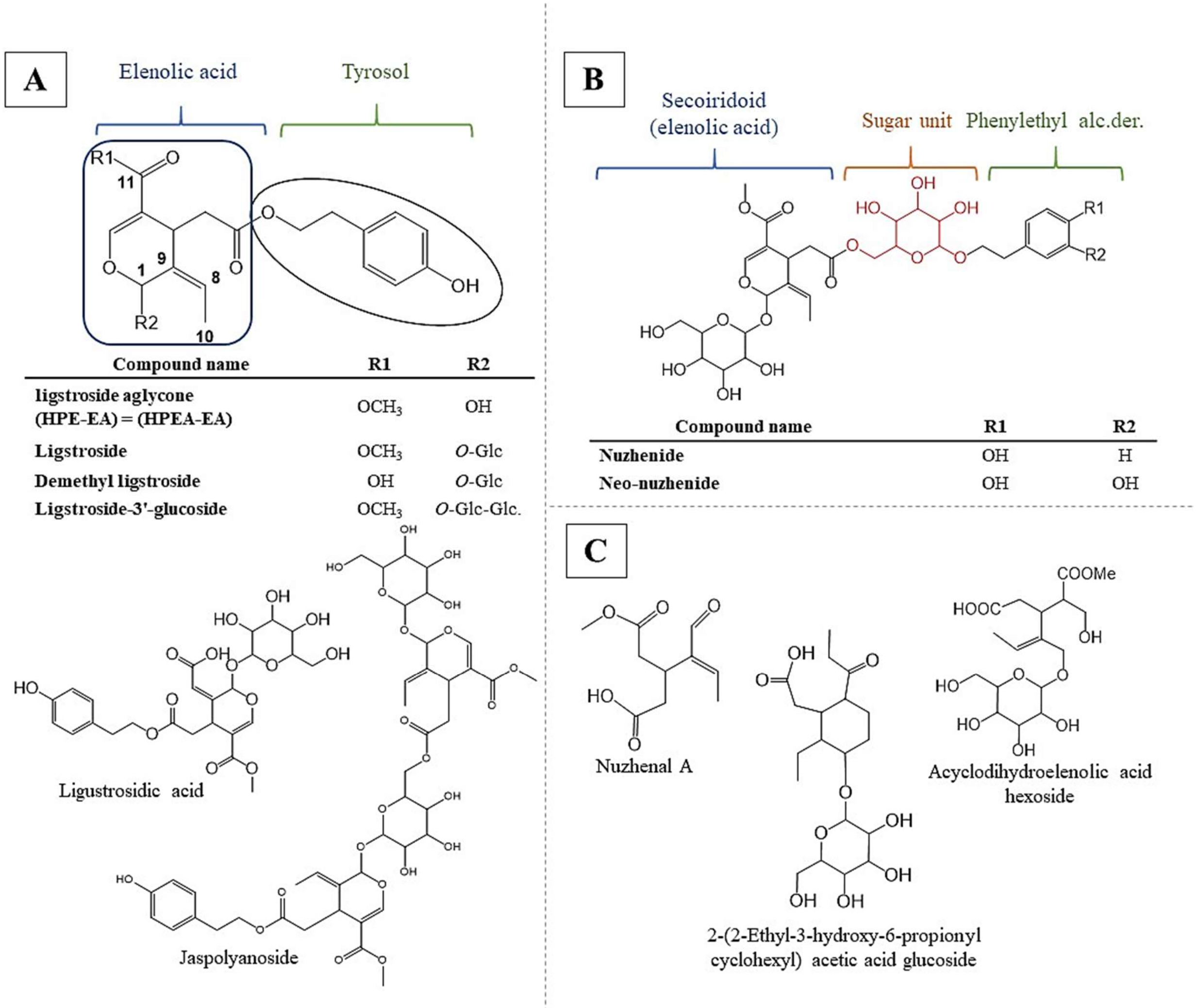

Figure S5. Chemical structures of tentatively identified compounds from OSE belonging to secoiridoid compounds; Ligstroside-type secoiridoids (A), Nuzhenide-type secoiridoids (B), and Other secoiridoids (C).

A

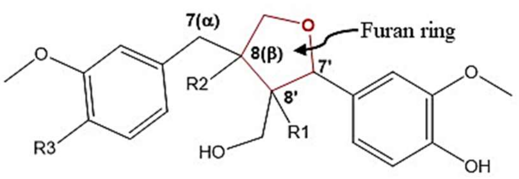

Compound name	R1	R2	R3
(−)-Olivil	H	OH	OH
(−)-Olivil 4-*O*-D glucopyranoside	H	OH	*O*-Glc

B

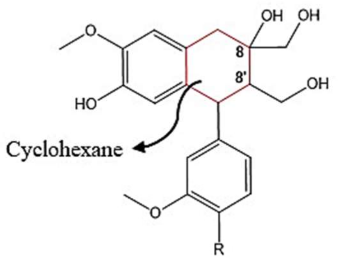

Compound name	R1
Cycloolivil glycoside	*O*-Glc
Cycloolivil	OH

C

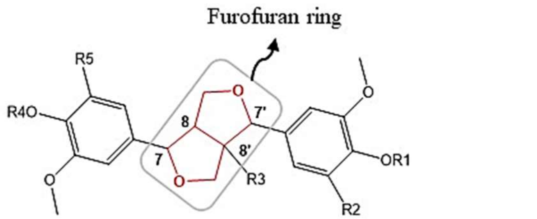

Compound name	R1	R2	R3	R4	R5
(+)-Pinoresinol	H	H	H	H	H
Pinoresinol glucoside	Glc	H	H	H	H
Hydroxypinoresinol	H	H	OH	H	H
1-Hydroxypinoresinol 4'-*β*-D glucopyranoside	Glc	H	OH	H	H
1-Acetoxypinoresinol	H	H	Ac	H	H
Acetoxypinoresinol 4'-*β*-D glucoside	Glc	H	Ac	H	H
Syringaresinol	H	OCH_3	H	H	OCH_3
Syringaresinol *O*-*β*-D glucoside	H	OCH_3	H	Glc	OCH_3
Hydroxysyringaresinol	H	OCH_3	OH	H	OCH_3
Fraxiresinol 1-*O*-*β*-D-glucoside	H	OCH_3	*O*-Glc	H	H
Eleutheroside E	Glc	OCH_3	H	Glc	OCH_3

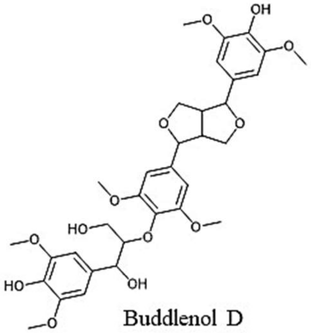

Buddlenol D

Figure S6. Chemical structures of tentatively identified compounds from OSE belonging to lignan compounds; olivil-based lignan (A), cyclolivil-based lignan (B), and pinoresinol-based lignan (C).

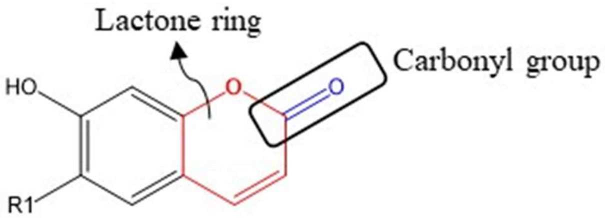

Compound name	R1
**Umbelliferone**	H
**Esculetin (aesculetin)**	OH
**Esculin/ aesculin (esculetin 6-*O*-glucoside)**	*O*-Glc.
**Scopoletin (6-methyl esculetin)**	OCH_3

Figure S7. Chemical structures of tentatively identified compounds from OSE belonging to hydroxycoumarins and derivatives.

A

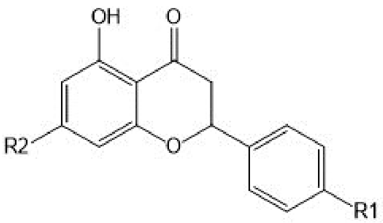

Compound name	R1	R2
**Naringenin**	OH	OH
**Naringenin hexoside**	OH	*O*-Hex.
**Dihydroxy flavanone**	H	OH

B

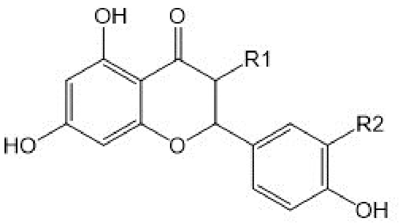

Compound name	R1	R2
**Dihydrokaempferol**	OH	H
**Taxifolin**	OH	OH
**Taxifolin 3-*O*-glucoside**	*O*-Glc.	OH

C

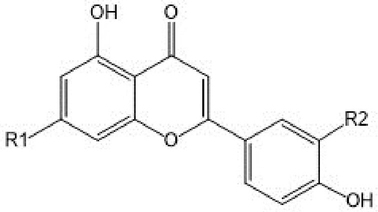

Compound name	R1	R4
**Luteolin**	OH	OH
**Chrysoeriol-7-*O*-glucoside**	*O*-Glc.	OCH_3
**Luteolin-7-*O*-hexoside**	*O*-hex.	OH
**Apigenin**	OH	H
**Apigenin 7-*O*-glucoside**	*O*-Glc.	H
**Apigenin 7-*O*-rutinoside (Isorhoifolin)**	*O*-Rham-Glc.	H

D

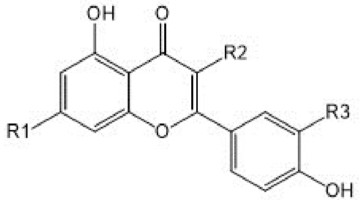

Compound name	R1	R2	R3
**Quercetin**	OH	OH	OH
**Isoquercitrin (quercetin 3-*O*-glucoside)**	OH	*O*-Glc.	OH
**Rutin (quercetin-3-*O*-rutinoside)**	OH	*O*-Rut.	OH
**Kaempferol-7-*O*-glucoside**	*O*-Glc.	OH	H

Figure S8. Chemical structures of tentatively identified compounds from OSE belonging to flavonoid compounds; flavanones (A), flavanonols (B), flavones (C), and flavonols (D).

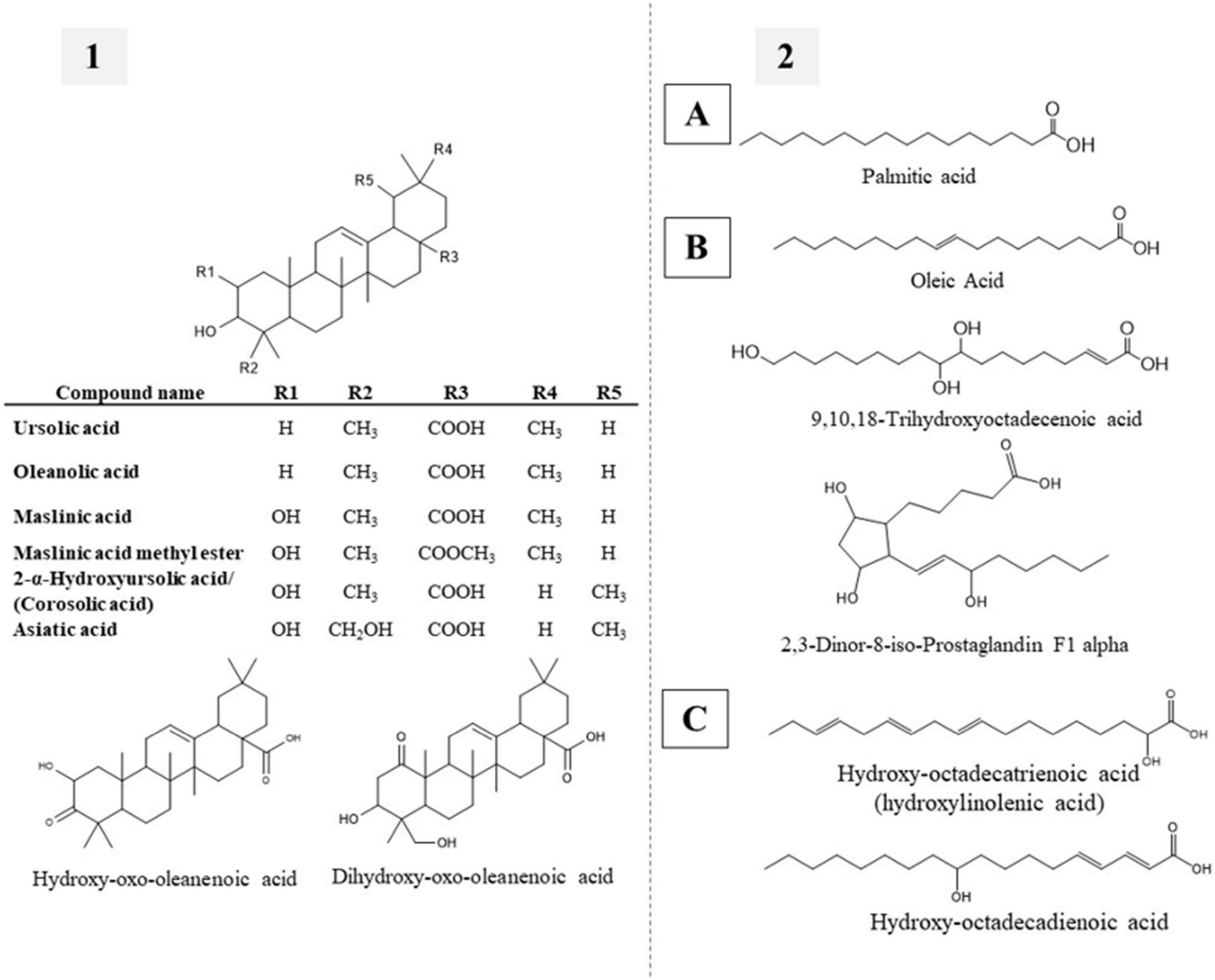

Compound name	R1	R2	R3	R4	R5
Ursolic acid	H	CH_3	COOH	CH_3	H
Oleanolic acid	H	CH_3	COOH	CH_3	H
Maslinic acid	OH	CH_3	COOH	CH_3	H
Maslinic acid methyl ester	OH	CH_3	$COOCH_3$	CH_3	H
2-α-Hydroxyursolic acid/ (Corosolic acid)	OH	CH_3	COOH	H	CH_3
Asiatic acid	OH	CH_2OH	COOH	H	CH_3

Figure S9. Chemical structures of tentatively identified compounds from OSE belonging to triterpenoids (1) and fatty acids (2) involving; saturated fatty acid (SFA) (A), monounsaturated fatty acids (MUFA) (B), and polyunsaturated fatty acids (PUFA) (C).

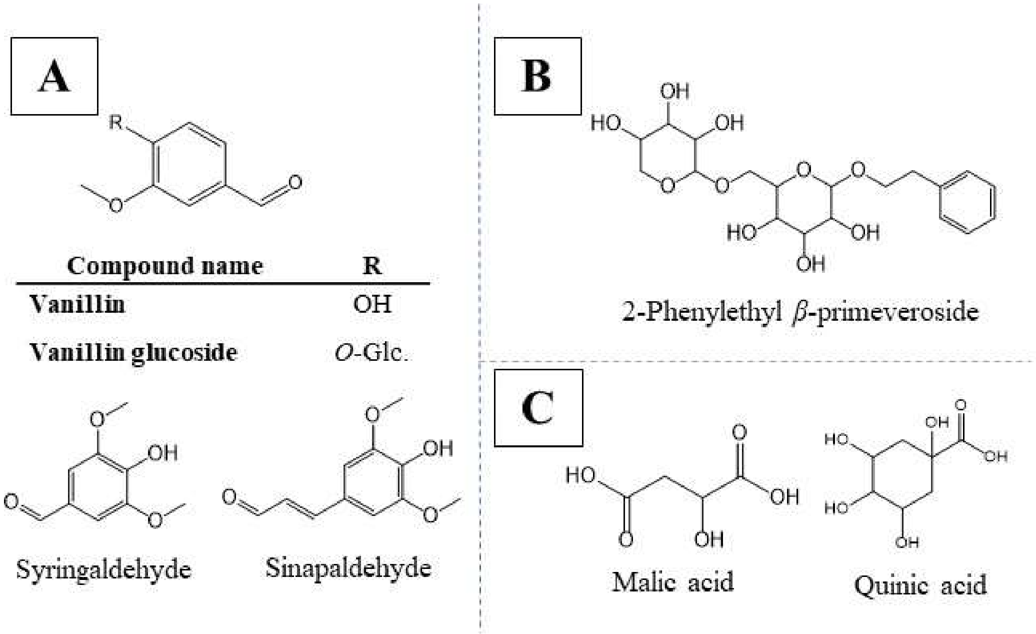

Figure S10. Chemical structures of tentatively identified compounds from OSE belonging to other compounds; aldehydic compounds (A), phenylethyl diglycosidic compound (B), and other acidic compounds (C).

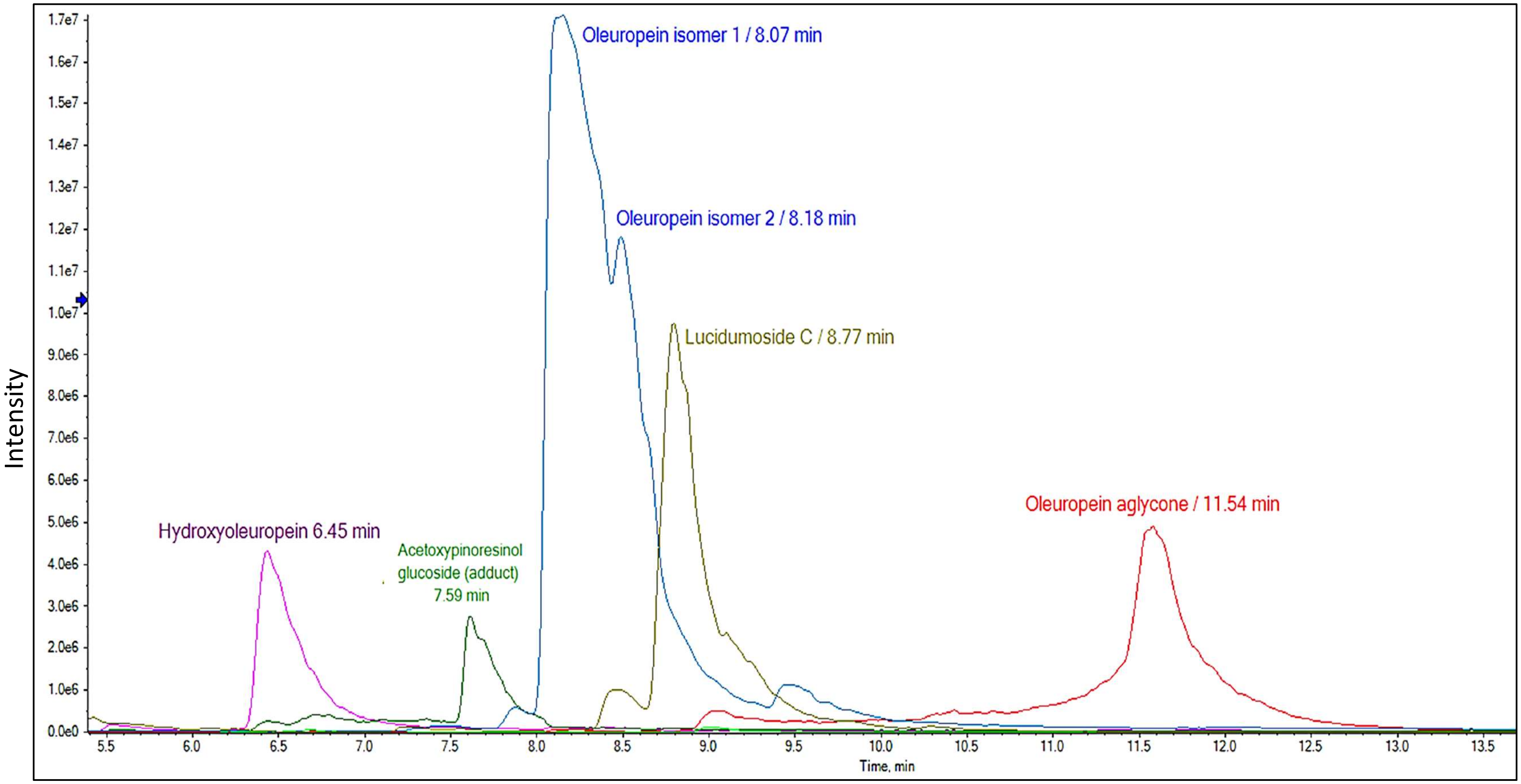

Figure S11. The five major compounds selected based on peak area from the extracted ion chromatogram (EIC) of ethanolic extract of olive stems using UPLC-HR-QTOF-MS detection in the negative ionization mode: oleuropein, lucidumoside C, hydroxyoleuropein, oleuropein aglycone, and acetoxypinoresinol hexoside.

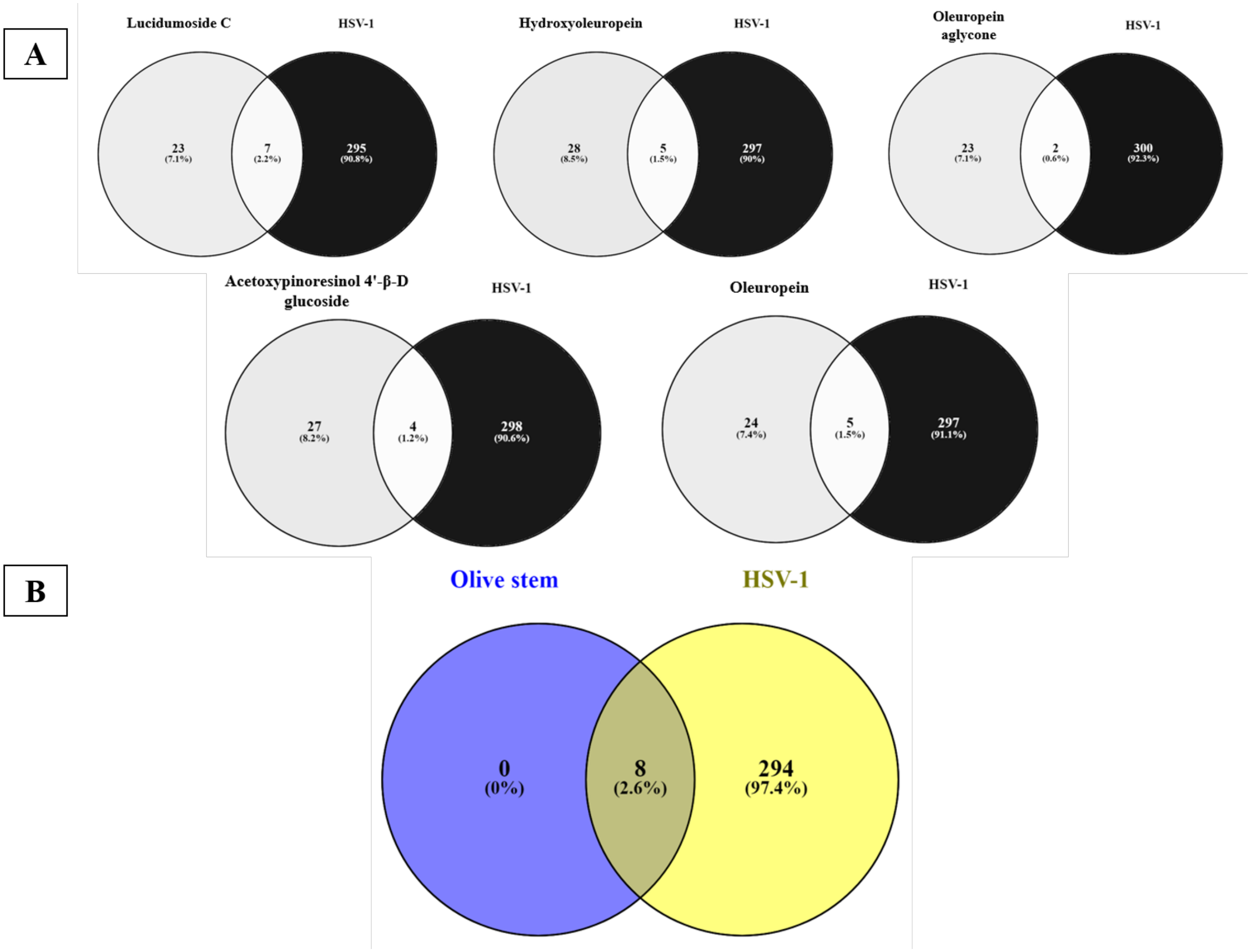

Figure S12. Venn diagram of the bioactive compounds of olive stem and herpes simplex virus type 1 (HSV-1) targets. A: A solid white diagram indicates targets of each bioactive compound separately with grey color while the black color indicates targets of HSV-1; B: A colored diagram showing blue color reveals targets of olive stem while the targets of HSV-1 are colored yellow.

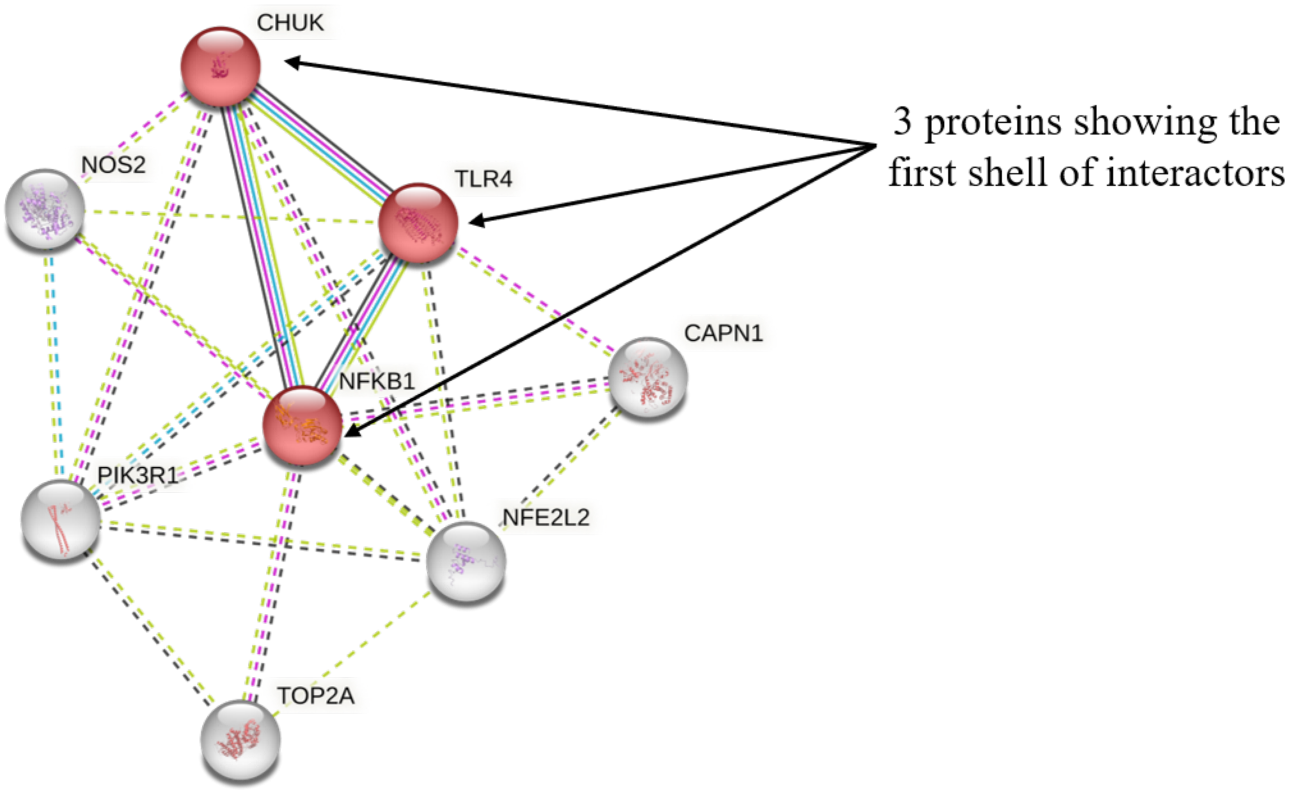

Figure S13. The Protein-Protein Interaction network created by the STRING database showed the interaction between different targets related to HSV-1.

[1] Abbattista, R., Ventura, G., Calvano, C. D., Cataldi, T. R. I. & Losito, I. Bioactive Compounds in Waste By-Products from Olive Oil Production: Applications and Structural Characterization by Mass Spectrometry Techniques. *Foods* **10**, 1236, doi:10.3390/foods10061236 (2021).

[2] Ammar, S., Contreras, M. D. M., Gargouri, B., Segura-Carretero, A. & Bouaziz, M. RP-HPLC-DAD-ESI-QTOF-MS based metabolic profiling of the potential Olea europaea by-product "wood" and its comparison with leaf counterpart. *Phytochem Anal* **28**, 217-229, doi:10.1002/pca.2664 (2017).

[3] Quirantes-Pine, R. *et al.* HPLC-ESI-QTOF-MS as a powerful analytical tool for characterising phenolic compounds in olive-leaf extracts. *Phytochem Anal* **24**, 213-223, doi:10.1002/pca.2401 (2013).

[4] Toumi, K., Swiatek, L., Boguszewska, A., Skalicka-Wozniak, K. & Bouaziz, M. Comprehensive Metabolite Profiling of Chemlali Olive Tree Root Extracts Using LC-ESI-QTOF-MS/MS, Their Cytotoxicity, and Antiviral Assessment. *Molecules* **28**, 4829, doi:10.3390/molecules28124829 (2023).

[5] Vergine, M. *et al.* Phenolic characterization of olive genotypes potentially resistant to Xylella. *Journal of Plant Interactions* **17**, 462-474, doi:10.1080/17429145.2022.2049381 (2022).

[6] Difonzo, G. *et al.* Metabolomics Approach to Characterize Green Olive Leaf Extracts Classified Based on Variety and Season. *Plants (Basel)* **11**, 3321, doi:10.3390/plants11233321 (2022).

[7] Huang, G. *et al.* Isolation and Identification of Chemical Constituents from Zhideke Granules by Ultra-Performance Liquid Chromatography Coupled with Mass Spectrometry. *J Anal Methods Chem* **2020**, 8889607, doi:10.1155/2020/8889607 (2020).

[8] Michel, T. *et al.* UHPLC-DAD-FLD and UHPLC-HRMS/MS based metabolic profiling and characterization of different Olea europaea organs of Koroneiki and Chetoui varieties. *Phytochemistry letters* **11**, 424-439 (2015).

[9] Gomez-Gonzalez, S., Ruiz-Jimenez, J., Priego-Capote, F. & Luque de Castro, M. D. Qualitative and quantitative sugar profiling in olive fruits, leaves, and stems by gas chromatography-tandem mass spectrometry (GC-MS/MS) after ultrasound-assisted leaching. *J. Agric. Food Chem.* **58**, 12292-12299, doi:10.1021/jf102350s (2010).

[10] Wodner, M., Lavee, S. & Epstein, E. Identification and seasonal changes of glucose, fructose and mannitol in relation to oil accumulation during fruit development in Olea europaea (L.). *Scientia Horticulturae* **36**, 47-54, doi:10.1016/0304-4238(88)90006-4 (1988).

[11] Zhao, H., Avena-Bustillos, R. J. & Wang, S. C. Extraction, Purification and In Vitro Antioxidant Activity Evaluation of Phenolic Compounds in California Olive Pomace. *Foods* **11**, 174, doi:10.3390/foods11020174 (2022).

[12] Nunes, A. *et al.* Potential therapeutic of olive oil industry by-products in skin health: a review. *Int. J. Food Sci. Technol.* **57**, 173-187, doi:10.1111/ijfs.15384 (2021).

[13] Skaltsounis, A.-L., Argyropoulou, A., Aligiannis, N. & Xynos, N. in *Olive and Olive Oil Bioactive Constituents* 333-356 (Elsevier, 2015).

[14] Tóth, G. *et al.* Phenolic profiling of various olive bark-types and leaves: HPLC–ESI/MS study. *Industrial Crops and Products* **67**, 432-438, doi:10.1016/j.indcrop.2015.01.077 (2015).

[15] Brahmi, F., Mechri, B., Flamini, G., Dhibi, M. & Hammami, M. Antioxidant activities of the volatile oils and methanol extracts from olive stems. *Acta Physiologiae Plantarum* **35**, 1061-1070, doi:10.1007/s11738-012-1144-2 (2012).

[16] Peralbo-Molina, A., Priego-Capote, F. & Luque de Castro, M. D. Tentative identification of phenolic compounds in olive pomace extracts using liquid chromatography-tandem mass spectrometry with a quadrupole-quadrupole-time-of-flight mass detector. *J. Agric. Food Chem.* **60**, 11542-11550, doi:10.1021/jf302896m (2012).

[17] Rashed, S. A., Saad, T. I. & El-Darier, S. M. Potential aptitude of four olive cultivars as anticancer and antioxidant agents: oleuropein content. *Rendiconti Lincei. Scienze Fisiche e Naturali* **33**, 195-203, doi:10.1007/s12210-022-01056-x (2022).

[18] Ryan, D., Antolovich, M., Prenzler, P., Robards, K. & Lavee, S. Biotransformations of phenolic compounds in Olea europaea L. *Scientia Horticulturae* **92**, 147-176, doi:10.1016/s0304-4238(01)00287-4 (2002).

[19] FooDB. *FooDB: The Food Database*, <https://foodb.ca> (2023).

[20] Chatzikonstantinou, A. V. *et al.* Production of hydroxytyrosol rich extract from Olea europaea leaf with enhanced biological activity using immobilized enzyme reactors. *Environ. Sci. Pollut. Res. Int.* **29**, 29624-29637, doi:10.1007/s11356-021-17081-6 (2022).

[21] Silvan, J. M. *et al.* Olive-Leaf Extracts Modulate Inflammation and Oxidative Stress Associated with Human H. pylori Infection. *Antioxidants (Basel)* **10**, 2030, doi:10.3390/antiox10122030 (2021).

[22] Melliou, E., Zweigenbaum, J. A. & Mitchell, A. E. Ultrahigh-pressure liquid chromatography triple-quadrupole tandem mass spectrometry quantitation of polyphenols and secoiridoids in california-style black ripe olives and dry salt-cured olives. *J. Agric. Food Chem.* **63**, 2400-2405, doi:10.1021/jf506367e (2015).

[23] Papageorgiou, C. S. *et al.* High-Yield Production of a Rich-in-Hydroxytyrosol Extract from Olive (Olea europaea) Leaves. *Antioxidants (Basel)* **11**, 1042, doi:10.3390/antiox11061042 (2022).

[24] Liao, X., Hu, F. & Chen, Z. Identification and Quantitation of the Bioactive Components in Osmanthus fragrans Fruits by HPLC-ESI-MS/MS. *J. Agric. Food Chem.* **66**, 359-367, doi:10.1021/acs.jafc.7b05560 (2018).

[25] Guo, N. *et al.* An LC-MS/MS method for the determination of salidroside and its metabolite p-tyrosol in rat liver tissues. *Pharm. Biol.* **52**, 637-645, doi:10.3109/13880209.2013.863946 (2014).

[26] Hashmi, M. A., Khan, A., Hanif, M., Farooq, U. & Perveen, S. Traditional Uses, Phytochemistry, and Pharmacology of Olea europaea (Olive). *Evid. Based Complement. Alternat. Med.* **2015**, 541591, doi:10.1155/2015/541591 (2015).

[27] Sanz, M. *et al.* LC-DAD/ESI-MS/MS study of phenolic compounds in ash (Fraxinus excelsior L. and F. americana L.) heartwood. Effect of toasting intensity at cooperage. *J. Mass Spectrom.* **47**, 905-918, doi:10.1002/jms.3040 (2012).

[28] Fu, S. *et al.* Qualitative screening of phenolic compounds in olive leaf extracts by hyphenated liquid chromatography and preliminary evaluation of cytotoxic activity against human breast cancer cells. *Anal. Bioanal. Chem.* **397**, 643-654, doi:10.1007/s00216-010-3604-0 (2010).

[29] Kabbash, E. M., Abdel-Shakour, Z. T., El-Ahmady, S. H., Wink, M. & Ayoub, I. M. Comparative metabolic profiling of olive leaf extracts from twelve different cultivars collected in both fruiting and flowering seasons. *Sci. Rep.* **13**, 612, doi:10.1038/s41598-022-27119-5 (2023).

[30] Zhang, C. *et al.* Comparative Evaluation of the Phytochemical Profiles and Antioxidant Potentials of Olive Leaves from 32 Cultivars Grown in China. *Molecules* **27**, 1292, doi:10.3390/molecules27041292 (2022).

[31] Boruga, V. M. & Vlaicu, B. E. Olive leaf extracts and bioactive phenolic compounds related to population health. *Journal of Agroalimentary Processes & Technologies* **27** (2021).

[32] Dauber, C. *et al.* Characterization and incorporation of extracts from olive leaves obtained through maceration and supercritical extraction in Canola oil: Oxidative stability evaluation. *Lwt* **160**, 113274, doi:10.1016/j.lwt.2022.113274 (2022).

[33] Liao, X., Hong, Y. & Chen, Z. Identification and quantification of the bioactive components in Osmanthus fragrans roots by HPLC-MS/MS. *J Pharm Anal* **11**, 299-307, doi:10.1016/j.jpha.2020.06.010 (2021).

[34] Taamalli, A. *et al.* Characterisation of phenolic compounds by HPLC-TOF/IT/MS in buds and open flowers of 'Chemlali' olive cultivar. *Phytochem Anal* **24**, 504-512, doi:10.1002/pca.2450 (2013).

[35] Rubio-Senent, F. *et al.* Isolation and identification of minor secoiridoids and phenolic components from thermally treated olive oil by-products. *Food Chem.* **187**, 166-173, doi:10.1016/j.foodchem.2015.04.022 (2015).

[36] Ben-Amor, I. *et al.* Phytochemical Characterization of Olea europea Leaf Extracts and Assessment of Their Anti-Microbial and Anti-HSV-1 Activity. *Viruses* **13**, 1085, doi:10.3390/v13061085 (2021).

[37] Salido, S., Perez-Bonilla, M., Adams, R. P. & Altarejos, J. Phenolic Components and Antioxidant Activity of Wood Extracts from 10 Main Spanish Olive Cultivars. *J. Agric. Food Chem.* **63**, 6493-6500, doi:10.1021/acs.jafc.5b02979 (2015).

[38] Pérez-Bonilla, M. *et al.* Isolation of antioxidative secoiridoids from olive wood (Olea europaea L.) guided by on-line HPLC–DAD–radical scavenging detection. *Food Chem.* **124**, 36-41, doi:10.1016/j.foodchem.2010.05.099 (2011).

[39] Ghorbel, A. *et al.* Extraction yield optimization of Oleaster (Olea europaea var. sylvestris) fruits using response surface methodology, LC/MS profiling and evaluation of its effects on antioxidant activity

and autophagy in HFF cells. *Journal of Food Measurement and Characterization* **15**, 4946-4959, doi:10.1007/s11694-021-01058-7 (2021).

[40] Abbattista, R., Losito, I., Calvano, C. D. & Cataldi, T. R. I. Exploring the isomeric precursors of olive oil major secoiridoids: An insight into olive leaves and drupes by liquid-chromatography and fourier-transform tandem mass spectrometry. *Foods* **10**, 2050, doi:10.3390/foods10092050 (2021).

[41] Dermeche, S., Nadour, M., Larroche, C., Moulti-Mati, F. & Michaud, P. Olive mill wastes: Biochemical characterizations and valorization strategies. *Process Biochemistry* **48**, 1532-1552, doi:10.1016/j.procbio.2013.07.010 (2013).

[42] Antunes, B. d. F. *et al.* Evaluation of physicochemical, bioactive composition and profile of fatty acids in leaves of different olive cultivars. *Revista Ceres* **68**, 511-520, doi:10.1590/0034-737x202168060002 (2021).

[43] Li, H. *et al.* Application of UHPLC-ESI-Q-TOF-MS to Identify Multiple Constituents in Processed Products of the Herbal Medicine Ligustri Lucidi Fructus. *Molecules* **22**, 689, doi:10.3390/molecules22050689 (2017).

[44] Silva, S. *et al.* Secoiridoids in olive seed: characterization of nüzhenide and 11-methyl oleosides by liquid chromatography with diode array and mass spectrometry. *Grasas y Aceites* **61**, 157-164, doi:10.3989/gya.087309 (2010).

[45] Mohammad-Beigi, H. *et al.* Oleuropein derivatives from olive fruit extracts reduce alpha-synuclein fibrillation and oligomer toxicity. *J. Biol. Chem.* **294**, 4215-4232, doi:10.1074/jbc.RA118.005723 (2019).

[46] Ma, B. *et al.* Simultaneous determination of Eleutheroside B and Eleutheroside E in rat plasma by high performance liquid chromatography-electrospray ionization mass spectrometry and its application in a pharmacokinetic study. *J. Chromatogr. B Analyt. Technol. Biomed. Life Sci.* **917-918**, 84-92, doi:10.1016/j.jchromb.2012.12.041 (2013).

[47] Brenes, M. *et al.* Pinoresinol and 1-acetoxypinoresinol, two new phenolic compounds identified in olive oil. *Journal of the American Oil Chemists' Society* **77**, 715-720, doi:10.1007/s11746-000-0115-4 (2000).

[48] Hanhineva, K. *et al.* Identification of novel lignans in the whole grain rye bran by non-targeted LC–MS metabolite profiling. *Metabolomics* **8**, 399-409, doi:10.1007/s11306-011-0325-0 (2011).

[49] Geana, E. I., Ciucure, C. T., Apetrei, I. M., Clodoveo, M. L. & Apetrei, C. Discrimination of Olive Oil and Extra-Virgin Olive Oil from Other Vegetable Oils by Targeted and Untargeted HRMS Profiling of Phenolic and Triterpenic Compounds Combined with Chemometrics. *Int. J. Mol. Sci.* **24**, 5292, doi:10.3390/ijms24065292 (2023).

[50] Antonia Nunes, M. *et al.* Olive pomace as a valuable source of bioactive compounds: A study regarding its lipid- and water-soluble components. *Sci. Total Environ.* **644**, 229-236, doi:10.1016/j.scitotenv.2018.06.350 (2018).

[51] Lopez-Salas, L. *et al.* Monitoring the Phenolic and Terpenic Profile of Olives, Olive Oils and By-Products throughout the Production Process. *Foods* **13**, 1555, doi:10.3390/foods13101555 (2024).

[52] Abaza, L., Taamalli, A., Nsir, H. & Zarrouk, M. Olive Tree (Olea europeae L.) Leaves: Importance and Advances in the Analysis of Phenolic Compounds. *Antioxidants (Basel)* **4**, 682-698, doi:10.3390/antiox4040682 (2015).

[53] Zhou, L., Li, J. & Yan, C. Simultaneous determination of three flavonoids and one coumarin by LC-MS/MS: Application to a comparative pharmacokinetic study in normal and arthritic rats after oral administration of Daphne genkwa extract. *Biomed. Chromatogr.* **32**, e4233, doi:10.1002/bmc.4233 (2018).

[54] Zeng, X. *et al.* UFLC-Q-TOF-MS/MS-Based Screening and Identification of Flavonoids and Derived Metabolites in Human Urine after Oral Administration of Exocarpium Citri Grandis Extract. *Molecules* **23**, 895, doi:10.3390/molecules23040895 (2018).

[55] Japon-Lujan, R. & Luque de Castro, M. D. Small branches of olive tree: a source of biophenols complementary to olive leaves. *J. Agric. Food Chem.* **55**, 4584-4588, doi:10.1021/jf070215t (2007).

[56] Olmo-Garcia, L. *et al.* Unravelling the Distribution of Secondary Metabolites in Olea europaea L.: Exhaustive Characterization of Eight Olive-Tree Derived Matrices by Complementary Platforms (LC-ESI/APCI-MS and GC-APCI-MS). *Molecules* **23**, 2419, doi:10.3390/molecules23102419 (2018).

[57] Iaria, D. L., Chiappetta, A. & Muzzalupo, I. A De novo Transcriptomic Approach to Identify Flavonoids and Anthocyanins "Switch-Off" in Olive (Olea europaea L.) Drupes at Different Stages of Maturation. *Front Plant Sci* **6**, 1246, doi:10.3389/fpls.2015.01246 (2015).

[58] Bensehaila, S., Ilias, F., Saadi, F. & Zaouadi, N. Phenolic compounds and antimicrobial activity of olive (Olea europaea L.) leaves. *Asian Journal of Dairy and Food Research* **41**, 237-241, doi:10.18805/ajdfr.DR-240 (2022).

[59] Rufino-Palomares, E. E. *et al.* Nutraceutical Role of Polyphenols and Triterpenes Present in the Extracts of Fruits and Leaves of Olea europaea as Antioxidants, Anti-Infectives and Anticancer Agents on Healthy Growth. *Molecules* **27**, 2341, doi:10.3390/molecules27072341 (2022).

[60] Stiti, N. & Hartmann, M. A. Nonsterol Triterpenoids as Major Constituents of Olea europaea. *J Lipids* **2012**, 476595, doi:10.1155/2012/476595 (2012).

[61] Xie, P. *et al.* Phenolic Compounds and Triterpenes in Different Olive Tissues and Olive Oil By-Products, and Cytotoxicity on Human Colorectal Cancer Cells: The Case of Frantoio, Moraiolo and Leccino Cultivars (Olea europaea L.). *Foods* **10**, 2823, doi:10.3390/foods10112823 (2021).

[62] Stiti, N., Triki, S. & Hartmann, M. A. Formation of triterpenoids throughout Olea europaea fruit ontogeny. *Lipids* **42**, 55-67, doi:10.1007/s11745-006-3002-8 (2007).

[63] Yangui, T., Chakroun, H., Dhouib, A. & Bouaziz, M. Biological Properties and Chemical Composition of Essential Oils from Fresh and Shade Dried Olive Leaves of Olea europaea L. Chemlali Cultivar. *Journal of Essential Oil Bearing Plants* **24**, 1389-1401, doi:10.1080/0972060x.2022.2026254 (2022).

[64] Jurisic Grubesic, R. *et al.* Antioxidant Capacity of Free Volatile Compounds from Olea europaea L. cv. Oblica Leaves Depending on the Vegetation Stage. *Antioxidants (Basel)* **10**, 1832, doi:10.3390/antiox10111832 (2021).

[65] Jimenez-Lopez, C. *et al.* Bioactive Compounds and Quality of Extra Virgin Olive Oil. *Foods* **9**, 1014, doi:10.3390/foods9081014 (2020).

[66] Guo, Z. *et al.* Chemical composition and nutritional function of olive (Olea europaea L.): a review. *Phytochemistry Reviews* **17**, 1091-1110, doi:10.1007/s11101-017-9526-0 (2017).

[67] Boukhebti, H., Chaker, A. N., Lograda, T. & Ramdani, M. Chemical and antimicrobial properties of essential oils of Olea europeaL. *Int J Pharmacol Toxicol* **5**, 42-46 (2015).

[68] Martínez, M., Fuentes, M., Franco, N., Sánchez, J. & de Miguel, C. Fatty Acid Profiles of Virgin Olive Oils from the Five Olive-Growing Zones of Extremadura (Spain). *Journal of the American Oil Chemists' Society* **91**, 1921-1929, doi:10.1007/s11746-014-2528-9 (2014).

[69] Ghanbari, R., Anwar, F., Alkharfy, K. M., Gilani, A. H. & Saari, N. Valuable nutrients and functional bioactives in different parts of olive (Olea europaea L.)-a review. *Int. J. Mol. Sci.* **13**, 3291-3340, doi:10.3390/ijms13033291 (2012).

[70] Ollivier, D., Artaud, J., Pinatel, C., Durbec, J. P. & Guerere, M. Triacylglycerol and fatty acid compositions of French virgin olive oils. Characterization by chemometrics. *J. Agric. Food Chem.* **51**, 5723-5731, doi:10.1021/jf034365p (2003).

[71] Alnusaire, T. S. *et al.* An In Vitro and In Silico Study of the Enhanced Antiproliferative and Pro-Oxidant Potential of Olea europaea L. cv. Arbosana Leaf Extract via Elastic Nanovesicles (Spanlastics). *Antioxidants (Basel)* **10**, 1860, doi:10.3390/antiox10121860 (2021).

[72] Jaqueline Maalouly, J. A. Phenolic Compounds from Diluted Acid Hydrolysates of Olive Stones: Effect of Overliming. *Advances in Crop Science and Technology* **01**, doi:10.4172/2329-8863.1000103 (2013).

[73] Conde, E. *et al.* Antioxidant activity of the phenolic compounds released by hydrothermal treatments of olive tree pruning. *Food Chem.* **114**, 806-812, doi:10.1016/j.foodchem.2008.10.017 (2009).

[74] Spinola, V., Llorent-Martinez, E. J., Gouveia, S. & Castilho, P. C. Myrica faya: a new source of antioxidant phytochemicals. *J. Agric. Food Chem.* **62**, 9722-9735, doi:10.1021/jf503540s (2014).
